# Chiropractic Care Is Associated with Frequency-Specific Reorganisation of Triple-Network Brain Dynamics: A Source-Localised EEG Study

**DOI:** 10.3390/brainsci16080818

**Published:** 2026-07-31

**Authors:** Usman Ghani, Imran Amjad, Imran Khan Niazi, Nitika Kumari, Kelly Holt, Moeez Ashfaque, Amit N. Pujari, Ernest Nlandu Kamavuako, Bernadette Murphy, Heidi Haavik

**Affiliations:** 1 Centre for Chiropractic Research, New Zealand College of Chiropractic, Auckland 1060, New Zealand; usman.ghani@nzchiro.co.nz (U.G.); imran.amjad@nzchiro.co.nz (I.A.); nitika.kumari@nzchiro.co.nz (N.K.); kelly.holt@nzchiro.co.nz (K.H.); heidi.haavik@nzchiro.co.nz (H.H.); 2 Faculty of Rehabilitation and Allied Health Sciences, Riphah International University, Islamabad 46000, Pakistan; 3 Centre for Sensory–Motor Interactions, Department of Health Science and Technology, Aalborg University, 9220 Aalborg, Denmark; 4 Faculty of Health & Environmental Sciences, Health & Rehabilitation Research Institute, Auckland University of Technology, Auckland 1010, New Zealand; 5 School of Physics, Engineering and Computer Science, University of Hertfordshire, Hatfield AL10 9AB, UK; m.ashfaque@herts.ac.uk (M.A.); a.pujari@herts.ac.uk (A.N.P.); 6 School of Engineering, University of Aberdeen, Aberdeen AB24 3FX, UK; 7 Centre for Robotics Research, Department of Informatics, King’s College London, London WC2G 4BG, UK; ernest.kamavuako@kcl.ac.uk; 8 Faculty of Health Sciences, Ontario Tech University, Oshawa, ON L1G 0C5, Canada

**Keywords:** chronic low back pain, chiropractic spinal manipulation, EEG source localisation, phase lag index, triple network model, default mode network, salience network, cortical oscillations, functional connectivity, central sensitization

## Abstract

**Highlights:**

**What are the main findings?**

- Chiropractic care was associated with distinct changes in connectivity within the default mode, salience, and executive networks.
- The exploratory analysis found resting-state connectivity changes that were still present four weeks later, alongside preliminary changes in the somatosensory evoked recordings.

**What are the implications of the main findings?**

- These findings identify brain-network coordination as a promising avenue for understanding responses to chiropractic care.
- They provide a foundation for future studies linking neural changes with pain and broader health outcomes.

**Abstract:**

Background/Objectives: Large-scale dysfunction in the default mode (DMN), salience (SN), and central executive (CEN) networks characterises conditions affecting affective, cognitive, and autonomic regulation. Chronic low back pain (CLBP) shows hyperactive salience signalling, disrupted DMN processing, and impaired executive regulation. Chiropractic adjustments modulate cortical excitability, yet whether these extend to oscillatory reorganisation is unknown. This exploratory secondary analysis aimed to determine whether chiropractic care is associated with frequency- and network-specific changes in source-localised EEG connectivity within and between the DMN, SN, and CEN in adults with CLBP. Methods: Seventy-six adults with CLBP were randomised to chiropractic plus usual care (n = 38) or usual care alone (n = 38). EEG was recorded during resting-state and SEP paradigms at baseline, post-intervention, and four weeks. Source activity was reconstructed with sLORETA and PLI computed across theta, alpha, beta, and gamma bands. For each condition, Fisher-z network-mean PLI was analysed in separate network-by-band linear mixed-effects models with Group, Session, and their interaction as fixed effects and a random intercept for a unique participant identifier; ROI-level changes were examined with cluster-based permutation tests, with inference at the connected-component level. Results: Resting-state alpha-band connectivity within the default mode network was higher in the chiropractic group both immediately after care and at four-week follow-up (both q<0.001), alongside additional frequency- and network-specific effects. Region-level analyses identified a distributed alpha-band network spanning most triple-network regions, in which resting-state connectivity increased in the chiropractic group after care ($p_{FWE}=0.003$; Holm-adjusted p=0.042). For the somatosensory evoked recordings, no network-level interaction survived multiple-comparison correction; the distributed connectivity changes identified in exploratory region-level analyses are therefore considered preliminary. Conclusions: Chiropractic adjustments are associated with frequency-specific changes in resting-state connectivity within the DMN, SN, and CEN. These findings are hypothesis-generating and require confirmation in adequately powered, pre-registered trials before they can be interpreted as a neurophysiological basis for improvements in pain, mood, fatigue, sleep, or quality of life.

## 1. Introduction

Chiropractic care commonly involves high-velocity low-amplitude (HVLA) spinal adjustments (often referred to as spinal manipulation in the research literature). From a neurophysiological perspective, these adjustments represent a mechanical stimulus applied to dysfunctional vertebral motion segments, providing rapid proprioceptive input from paraspinal tissues and their associated mechanoreceptors [1]. Experimental studies using techniques such as transcranial magnetic stimulation, somatosensory evoked potentials (SEPs), and neuroimaging have demonstrated that spinal adjustments can modulate cortical excitability, alter sensorimotor integration, and influence motor control through projections to spinal, cerebellar, and cortical structures involved in movement control and sensory integration [2,3,4,5,6,7,8,9,10]. Together, these findings provide evidence that spinal adjustments of dysfunctional segments can influence central neural processing [11] and may therefore have downstream effects on large-scale brain network dynamics [10,12].

Recent electroencephalography (EEG) investigations have begun to characterise the effects of chiropractic care on brain network connectivity [10,13]. A pilot study in stroke survivors found that spinal manipulation increased alpha-band functional connectivity within the default mode network (DMN) [10]. In a randomised controlled trial involving individuals with chronic low back pain (CLBP), four weeks of chiropractic care was associated with significant changes in resting-state EEG spectral power, alterations in somatosensory-evoked potentials, and improvements in multiple clinical outcomes, including pain, sleep, mood, and quality of life [12]. Source-localised analyses from that study also revealed increased alpha activity within the default mode network following chiropractic care, suggesting that spinal manipulation may influence large-scale brain network dynamics [12]. These findings highlighted the importance of determining whether the observed changes extend beyond the default mode network to other large-scale brain networks involved in salience detection and executive control.

One plausible pathway through which spinal adjustments may influence cortical network dynamics involves the relay of paraspinal mechanoreceptor and muscle spindle afferent signals through the dorsal horn and thalamus, with thalamocortical projections subsequently modulating cortical oscillatory activity and large-scale network organisation [1]. A reduction in nociceptive drive through decreased peripheral sensitisation or local inflammation represents an alternative pathway that may also contribute to the observed central changes. These mechanistic accounts are not mutually exclusive and cannot be disambiguated by the present data.

Chronic pain conditions are increasingly understood as involving dysfunction of large-scale brain networks that regulate self-referential processing, salience detection, and executive control [14]. Neuroimaging studies have consistently identified altered connectivity patterns across three key intrinsic brain networks: the DMN, salience network (SN), and central executive network (CEN) [14,15,16,17]. Together, these networks form part of the “triple network model,” which has been proposed as a central organising framework for understanding the neural basis of cognitive and affective dysfunction across a range of neurological and psychiatric conditions, including chronic pain [14,18,19,20].

Within this framework, the DMN, which includes the medial prefrontal cortex, posterior cingulate cortex, and precuneus, is associated with internally directed cognition and self-referential processing [21]. Altered DMN connectivity has been widely reported in chronic pain populations and is thought to contribute to persistent pain-related rumination and maladaptive self-referential processing [15,16,22,23,24]. The SN, anchored in the anterior insula and dorsal anterior cingulate cortex, plays a critical role in detecting behaviourally relevant stimuli and allocating attentional resources [25]. In chronic pain populations, altered salience network connectivity has been reported and is thought to contribute to abnormal prioritisation of pain-related signals [15,17,24,26,27,28,29]. The CEN, comprising the dorsolateral prefrontal cortex and lateral posterior parietal cortex, supports higher-order executive processes such as cognitive control, working memory, and goal-directed behaviour [30]. Alterations in connectivity within this network have been reported in chronic pain populations [14,23].

Importantly, emerging evidence suggests that both chronic pain and a range of neurological and psychiatric conditions are associated not only with within-network alterations, but also with disrupted functional interactions between these large-scale networks. In particular, aberrant coupling between the DMN, SN, and CEN has been linked to impaired network switching, reduced cognitive flexibility, and the persistence of maladaptive perceptual and affective states [19,21,25]. For example, altered SN-mediated switching between internally focused DMN activity and externally oriented CEN engagement has been proposed as a key mechanism underlying sustained pain-related attention and difficulty disengaging from pain [14,22]. Such between-network dysregulation has been reported across multiple conditions, suggesting a shared systems-level mechanism underlying persistent dysfunction across conditions.

The present study therefore used data from that randomised controlled trial to conduct an exploratory secondary analysis examining the immediate and longer-term effects of chiropractic spinal adjustments on EEG source-level functional connectivity within and between the DMN, SN, and CEN in adults with chronic low back pain. Functional connectivity was assessed using the phase lag index (PLI), which was chosen because it reduces sensitivity to zero-lag volume conduction and common-source effects [31,32]. Source-localised connectivity was analysed across standard frequency bands during both resting-state and somatosensory-evoked conditions at baseline, immediately following the initial intervention, and after four weeks of care. The primary aim of this study was to address an exploratory research question motivated by prior observations in this cohort [12]: whether chiropractic spinal adjustments are associated with frequency- and network-specific alterations in connectivity within and between the DMN, SN, and CEN. Given the secondary and exploratory nature of this analysis, findings should be considered hypothesis-generating rather than confirmatory.

## 2. Methods

### 2.1. Study Design and Participants

This exploratory secondary analysis used data from a parallel group randomised controlled trial (RCT) described in our previously published study [12]. The experimental protocol (participant recruitment, randomisation, and intervention procedures) follows that described therein. In total, 76 adults aged 18–60 years with non-specific CLBP (pain persisting for at least three months within the past year, between lower rib margins and buttock crease; see [33] for standardised pain definitions) were recruited through community advertisement. Inclusion criteria were an age of 18 to 60 years, non-specific CLBP as defined above, sufficient English to complete the study assessments, and the capacity to provide written informed consent. Exclusion criteria included pain >7/10 on a visual analogue scale (VAS), prior spinal surgery, metabolic, inflammatory, or neoplastic disease, contraindications to assessment, and inability to attend study sessions. These criteria selected a clinically homogeneous CLBP sample and reduced the influence of comorbid neurological or systemic disease on the EEG measures.

Following baseline assessments and consent, participants were randomly assigned (block randomisation: n = 38 per group) to receive either four weeks of chiropractic intervention (usual care plus spinal adjustments) or a usual care control (medically recommended CLBP care alone). The study protocol was approved by the King’s College London Research Ethics Panel and the New Zealand College of Chiropractic Research Committee. All participants provided written informed consent. The triple-network connectivity analysis reported here was an exploratory secondary analysis, not pre-specified in the original trial protocol [12]. It was motivated by observations in the parent study and should be interpreted as hypothesis-generating. Neither the analysis nor its hypotheses were pre-registered. The analysis was motivated by the parent study, in which source-localised EEG revealed increased alpha-band connectivity within the DMN following chiropractic care [12], consistent with an earlier pilot in stroke survivors [10]. Because the DMN does not operate in isolation but interacts dynamically with the SN and CEN within the triple-network model of chronic pain, we reasoned that a DMN-restricted analysis could misrepresent a change that was in fact part of a broader, coordinated network reorganisation. We therefore chose the triple-network model as the interpretive framework: it defines a small, theory-driven set of networks (DMN, SN, and CEN) with established relevance to chronic pain, which extends the analysis beyond the DMN while limiting the number of connectivity comparisons and reducing the arbitrary region selection that can inflate false-positive findings in exploratory connectivity work. In this way, it served both to situate the earlier DMN observation within the wider network system and as a form of interpretive control on the analysis.

The number of participants contributing data to each analysis is summarised in Table 1. Of the 76 randomised participants, 29 chiropractic and 29 to 32 control participants contributed to the network-level analyses, depending on the frequency band. Participants were not analysed where the recording was incomplete, where artefact rejection left insufficient data, or where the derived band-specific connectivity matrix was unavailable. No participant was excluded for reasons related to group allocation.

**Table 1:** Participant flow and the analysed EEG sample by group and frequency band. The analysed sample differed slightly across bands because the derived band-specific connectivity matrices were not available for every participant. The same analysed sample sizes applied to the resting-state and SEP network-level analyses; therefore the values are shown once by frequency band rather than repeated by recording condition. Satterthwaite degrees of freedom in Table 2 and Table 3 additionally reflect the repeated participant-session responses and fitted variance components; they are not counts of independent participants.

Stage	Chiropractic	Control
Randomised	38	38
Analysed, alpha band	29	32
Analysed, theta, beta, and gamma bands	29	29
Not analysed (incomplete recording, excessive artefact, or unavailable band-specific connectivity matrix)	9	6 to 9

**Table 2:** Resting-state model-estimated chiropractic-minus-control contrasts at Post and Post4W that met the FDR threshold of q<0.05. Estimates and SEs are on the Fisher-z scale. The estimate, SE, df, t-statistic, and contrast *q*-value come from the same random-intercept LMM. Fractional df values are Satterthwaite denominator degrees of freedom based on the repeated participant-session responses and fitted variance components in the corresponding LMM; they are not counts of independent participants. $d_{\mathrm{LMM}}$ is the contrast divided by the residual SD from that model and is descriptive. Interaction q is the FDR-corrected Type III Group-by-Session test for the corresponding network-by-band model.

Band	Net.	Session	Estimate	SE	df	t	*q*	$d_{\mathrm{LMM}}$	Int. *q*
Theta	DMN	Post	−0.024	0.003	164.8	−6.96	<0.001	−1.93	<0.001
Theta	DMN	Post4W	−0.025	0.003	164.8	−7.27	<0.001	−2.01	<0.001
Theta	SN	Post	+0.028	0.005	163.4	5.58	<0.001	+1.56	<0.001
Theta	SN	Post4W	+0.021	0.005	163.4	4.14	<0.001	+1.16	<0.001
Theta	CEN	Post	+0.050	0.004	157.8	12.93	<0.001	+3.75	<0.001
Theta	CEN	Post4W	+0.035	0.004	157.8	9.10	<0.001	+2.64	<0.001
Alpha	DMN	Post	+0.106	0.003	173.6	31.64	<0.001	+8.54	<0.001
Alpha	DMN	Post4W	+0.088	0.003	173.6	26.15	<0.001	+7.06	<0.001
Alpha	SN	Post	−0.030	0.005	170.6	−5.97	<0.001	−1.65	<0.001
Alpha	SN	Post4W	−0.013	0.005	170.6	−2.52	0.027	−0.69	<0.001
Alpha	CEN	Post	−0.027	0.004	177.0	−7.28	<0.001	−1.88	<0.001
Alpha	CEN	Post4W	−0.021	0.004	177.0	−5.59	<0.001	−1.44	<0.001
Beta	DMN	Post	−0.061	0.018	149.3	−3.33	0.003	−1.01	0.012
Beta	DMN	Post4W	−0.049	0.018	149.3	−2.66	0.020	−0.81	0.012
Beta	CEN	Post	+0.061	0.022	150.4	2.79	0.014	+0.84	0.153
Gamma	DMN	Post	+0.148	0.026	167.8	5.59	<0.001	+1.49	0.004
Gamma	DMN	Post4W	+0.155	0.026	167.8	5.87	<0.001	+1.56	0.004

**Table 3:** Group-by-Session interaction tests (Type III *F*-tests) for the condition-specific SEP network-mean PLI models. Numerator and Satterthwaite denominator degrees of freedom are shown; *q* is the Benjamini–Hochberg FDR-adjusted *p*-value within the family of 12 interaction tests, and Holm *p* is the corresponding Holm-adjusted value. Complete between-group and within-group session contrasts appear in Supplementary Tables S2 and S4.

Band	Net.	NumDF	DenDF	F	*p*	*q*	Holm	Sig.
Theta	DMN	2	177.0	2.92	0.057	0.170	0.509	ns
Theta	SN	2	118.0	3.29	0.041	0.163	0.407	ns
Theta	CEN	2	177.0	1.79	0.171	0.292	1.000	ns
Alpha	DMN	2	177.0	3.58	0.030	0.163	0.328	ns
Alpha	SN	2	177.0	0.18	0.835	0.835	1.000	ns
Alpha	CEN	2	177.0	4.87	0.009	0.105	0.105	ns
Beta	DMN	2	118.0	0.43	0.652	0.711	1.000	ns
Beta	SN	2	118.0	1.30	0.276	0.368	1.000	ns
Beta	CEN	2	118.0	2.36	0.099	0.238	0.793	ns
Gamma	DMN	2	118.0	2.00	0.140	0.280	0.979	ns
Gamma	SN	2	118.0	0.74	0.482	0.578	1.000	ns
Gamma	CEN	2	118.0	1.62	0.203	0.304	1.000	ns

### 2.2. Chiropractic Care (Intervention)

An experienced, registered chiropractor administered chiropractic care, which included high-velocity, low-amplitude (HVLA) manual adjustments targeted to spinal or pelvic joints identified as restricted or dysfunctional based on clinical indicators (restricted intersegmental movement, tenderness to palpation, asymmetrical muscle tension, abnormal joint play/end-feel) [34,35]. These clinical indicators are commonly used in chiropractic practice, although inter-rater reliability was not assessed in the present study. Participants received chiropractic care three times a week for four weeks, with each visit lasting ~15 min. EEG was recorded immediately before and after the first intervention session and again after the 4-week intervention period. Participants continued any treatments prescribed by their usual healthcare providers during the study period; additional non-chiropractic treatments were not standardised or tracked. Blinding of the treating chiropractor was not possible due to the nature of the intervention. A single chiropractor delivered all interventions, which standardised treatment delivery but may limit generalizability.

### 2.3. Usual Care (Control)

Usual care referred to any care recommended or prescribed by non-chiropractic health providers for CLBP, including self-management advice, pharmacologic pain management, physical therapy, or referral to a pain clinic. In addition to usual care, participants underwent the same spinal and pelvic dysfunction assessment and positioning procedures as the chiropractic care group during the initial control session, but HVLA thrusts were withheld. This initial control session was designed to control for non-specific effects of manual contact, positioning, and movement associated with preparing for an adjustment. During these setup procedures, the chiropractor was careful not to thrust on the spine or to apply end-range tension to a vertebral segment. The duration of the initial control session was similar to that of the chiropractic care visits. No further intervention sessions occurred after this initial control session, although participants returned for follow-up assessment sessions at the same time points as the chiropractic group. Concurrent treatments during the study period, including medication use, physiotherapy, exercise, and changes in sleep, were not standardised or systematically recorded in either group. This information was therefore not available for the present analysis. We address the implications of this in the limitations.

### 2.4. Blinding

Blinding of participants and outcome assessors was implemented. Participants were not informed of their group allocation. All data were coded and analysed by blinded investigators. Due to the intervention’s nature, chiropractors could not be blinded; however, both groups were queried about their perceived treatment group assignment after the intervention to assess blinding efficacy [12].

### 2.5. EEG Acquisition and Preprocessing

Electroencephalography (EEG) was recorded using a 64-channel Brainwave EEG cap coupled with a REFA amplifier (TMSi, Oldenzaal, The Netherlands) at a sampling rate of 2048 Hz. Recordings were obtained from 64 scalp sites using the 10–20 electrode system [36], with the ground electrode at AFz and both mastoids (M1 and M2) as reference electrodes. Electrode impedance was maintained below 10 kΩ throughout data acquisition.

#### 2.5.1. Resting-State EEG Recording

Participants completed a single 5 min eyes-open resting-state recording while fixating on a central cross. They were seated comfortably in a chair and instructed to minimise movement and blinking during the recording.

#### 2.5.2. Somatosensory Evoked Potentials (SEPs)

The median nerve was stimulated using electrical pulses delivered by an electrical stimulator (Digitimer DS7AH, Welwyn Garden City, UK) to evoke SEPs, as in our previous studies [2,37]. Stimulation electrodes (Neuroline 700, AMBU A/S, Ballerup, Denmark) were placed at the left wrist. Motor threshold was defined as the minimum current intensity eliciting a visible thumb twitch. Before and after chiropractic care or control, 1000 electrical pulses were delivered to the median nerve. The stimulation pulse was monophasic, with a width of 0.2 ms and a frequency of 2.3 Hz.

#### 2.5.3. EEG Preprocessing

Raw EEG data were pre-processed offline using EEGLAB (version 14.1.1) [38] and ERPLAB (version 6.1.4) [39] running on MATLAB (2015b) (The MathWorks, Inc., Natick, MA, USA). Of the 64 recorded channels, 62 scalp electrodes were re-referenced to a common average reference, excluding the two mastoids. The PREP pipeline (version 0.55.1) [40] was used to remove and interpolate bad channels and to remove line noise.

For SEP preprocessing, stimulus-locked epochs were extracted and baseline-corrected using the pre-stimulus period. Bad epochs were visually removed before running independent component analysis (ICA). Custom-written ICA code was then used to remove bad components from the SEP and continuous EEG data. The SEP data obtained after ICA were subjected to the ERPLAB moving-window threshold-based artefact-detection algorithm [39]. A 20 ms window width and a 5 ms step were defined with a threshold of ±100 µV. Epochs in which the signal exceeded ±100 µV on any channel were rejected.

For resting-state EEG preprocessing, continuous data underwent the same PREP re-referencing, bad-channel interpolation, and line-noise removal steps used for the SEP data, followed by the same custom-written ICA implementation. This preprocessing pipeline produced the cleaned data used for subsequent analysis.

Bad channels were identified and interpolated automatically by the PREP pipeline. After visual rejection of bad SEP epochs, custom-written ICA code was applied to SEP and continuous EEG data to remove artefactual components. The available preprocessing documentation does not record the component-rejection criterion, so it cannot be reported retrospectively. For SEP data, post-ICA artefact rejection used the ERPLAB moving-window threshold of ±100 µV with a 20 ms window and 5 ms step. The same preprocessing implementation and documented thresholds were applied to every recording in both groups and at every session. Epoch retention was quantified from the cleaned recordings. For the SEP condition, of the 1000 stimulus-locked epochs per recording, the chiropractic group retained 919.1±88.2 (Pre), 900.7±136.6 (Post), and 928.0±67.0 (Post4W) epochs, and the control group retained 927.8±71.0, 943.0±61.1, and 937.5±76.9 epochs (mean ± SD), corresponding to between 5.7% and 8.7% of epochs rejected. For the resting-state condition, the chiropractic group retained 540.7±129.6, 522.0±117.4, and 513.3±121.8 epochs of 0.5 s, and the control group retained 531.6±80.9, 487.0±105.6, and 483.8±117.4 epochs. These retained resting-state epochs corresponded to approximately 4.0 to 4.5 min of analysable EEG after artefact rejection, with between 8.5% and 12.8% of epochs rejected. The proportion of epochs rejected did not differ between groups at any session (SEP, all p≥0.15; resting state, all p≥0.14; Welch’s t-tests) or across sessions within either group (all F≤0.89, all p≥0.41; one-way ANOVA). Differential data quality is therefore unlikely to account for the group differences reported in the network-level Results. These summaries describe all recordings that completed preprocessing (chiropractic n=29 to 32; control n=32 to 34); the network-level contrasts were run on the participants for whom band-specific connectivity matrices were available, as set out in Table 1. A sensitivity comparison restricted to participants with complete data at all three sessions led to the same conclusion (all between-group p≥0.10). The preprocessing derivatives available for this secondary analysis did not include per-recording counts of interpolated channels or ICA components removed. These quantities were therefore unavailable for analysis.

### 2.6. Source Localisation and Triple Network

**Figure 1:**
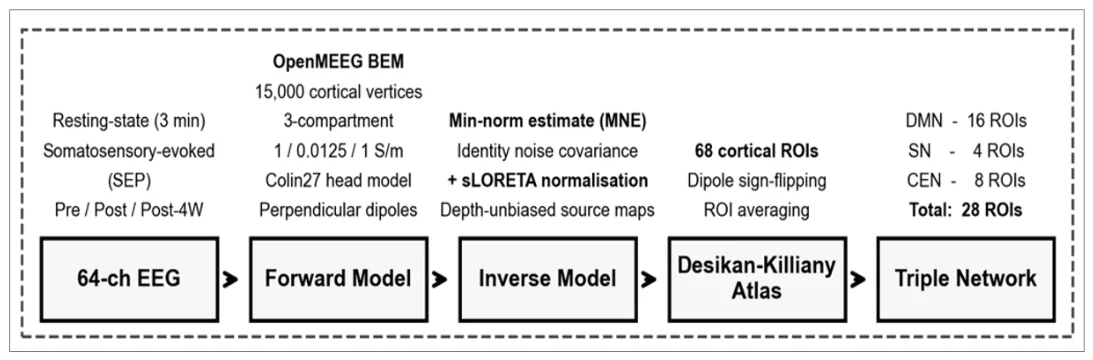
Source localization and triple network extraction pipeline.

Source-level EEG analyses were conducted in Brainstorm [41] using a two-stage source reconstruction pipeline using MATLAB R2022b. For the forward model, a symmetric boundary element method, OpenMEEG [42], was applied to a default generic head model [43] with 15,000 cortical vertices and a three-compartment geometry. Tissue conductivities were set to scalp = 1, skull = 0.0125, and brain = 1 S/m, consistent with established BEM parameterisation for EEG [44]. Electrode locations were defined according to the 10–20 system using the Colin27 ASA generic coordinates, and dipole orientations were constrained to be perpendicular to the cortical surface, reflecting the geometry of pyramidal neuron postsynaptic potentials. The source-localisation and triple-network extraction pipeline is summarised in Figure 1.

**Figure 2:**
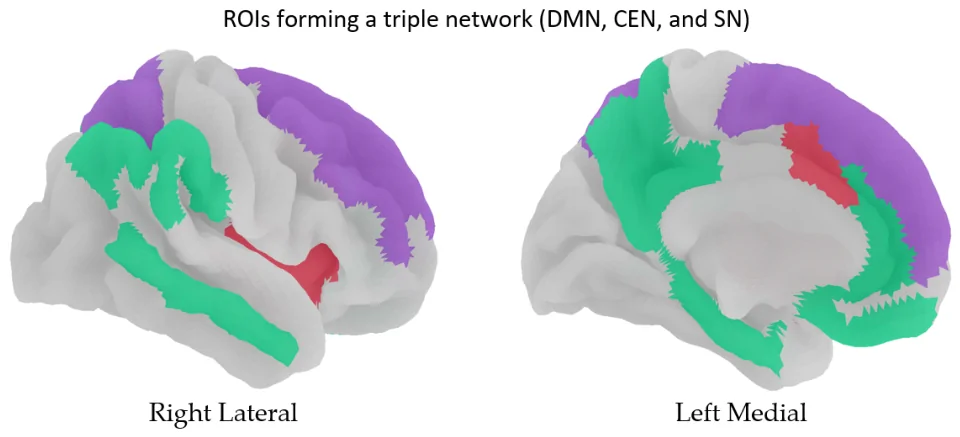
Brain regions forming the triple network. Purple: CEN, Green: DMN, Red: Salience.

Source time series were extracted by parcellating the reconstructed cortical surface according to the Desikan–Killiany atlas [45], yielding 68 cortical regions of interest (ROIs). Parcellation was fully atlas-based and automated, so it did not involve manual region drawing and did not require an inter-rater reliability assessment. Before averaging, dipole sign-flipping was applied within each ROI to prevent cancellation of activity arising from opposing dipole orientations. ROIs were then assigned to the default mode network (DMN), salience network (SN), and central executive network (CEN) following the established triple-network framework [14,19]. Network-mean time series were derived by averaging across all ROIs within each network; region-level time series were retained separately for subsequent ROI-to-ROI connectivity analyses. Selected ROIs are presented in Figure 2 with abbreviations and details in Table 4.

### 2.7. Functional Connectivity Analysis

Functional connectivity between sources/regions was quantified using the phase lag index (PLI), an estimator that reduces sensitivity to zero-lag volume conduction artefacts and is suitable for assessing phase–lagged interactions [31,32]. Narrowband signals were extracted using 4th-order Butterworth filters for theta, alpha, beta, and gamma bands [46]. PLI was computed pairwise between all ROIs, both within and between networks, at all time points (pre-, post-, and 4-week follow-up). For the resting-state condition, PLI was estimated from the continuous 5-minute recording. For the SEP condition, the retained stimulus-locked epochs of each recording were averaged before connectivity estimation, yielding one evoked sensor average per participant and session. That average was projected to source space, band-pass filtered, and PLI was computed across the resulting 512-sample interval, which spans −100 to +149 ms relative to stimulus onset at the 2048 Hz sampling rate. SEP connectivity therefore characterises the phase-lag structure of the averaged evoked response rather than trial-to-trial phase coupling of ongoing activity, and the interval contains fewer than two theta cycles and approximately two to three low-alpha cycles. SEP findings are accordingly reported as preliminary throughout, and the theta-band SEP results in particular should not be interpreted as evidence of ongoing oscillatory coupling.

The SEP connectivity matrices were derived from the condition-specific stimulus-locked sensor recordings. Sensor-to-source projection used the archived common source operator; the phase lag index was then computed per band across the full stimulus-locked interval, and the network- and region-level analyses reported below were applied to these condition-specific SEP matrices. A participant-level comparison of the resting-state and SEP network-mean values confirmed that the two conditions are numerically distinct (see Section 4 and the Supplementary Material).

## 3. Statistical Analysis

### 3.1. Network-Level Analysis

For the network-level models, participant was the independent experimental unit and participant-session was the repeated observation. Within each recording condition, frequency band, and network, each participant contributed one network-mean PLI value at Pre, Post, and Post4W. ROI-pair values were averaged before modelling; ROI pairs, epochs, and time windows were not entered as separate observations. A group-prefixed participant identifier ensured that identifiers shared across groups were treated as different participants.

Network-mean PLI was Fisher-z transformed before analysis. Separate linear mixed-effects models were fitted for the 12 network-by-band combinations within each recording condition. Each model included fixed effects of Group, Session, and their interaction, with a random intercept for participant. Models were fitted by restricted maximum likelihood using nloptwrap. A Session random slope was not identifiable with only three observations per participant, so the random-intercept specification was used consistently. Satterthwaite denominator degrees of freedom and *p*-values were obtained with lmerTest. The fractional degrees of freedom therefore reflect the repeated participant-session responses and fitted variance components, not additional independent participants. Analysed sample sizes are reported in Table 1.

The Group-by-Session interaction was tested with a Type III F-test. Planned model-estimated contrasts compared groups at each session and sessions within each group. Estimates, standard errors, t-statistics, degrees of freedom, and *p*-values were taken directly from the fitted LMM. The descriptive effect size $d_{\mathrm{LMM}}$ was calculated as the Fisher-z contrast divided by the residual standard deviation from the same model and was not used to calculate the t-statistic. Figures show back-transformed model-estimated marginal means or raw PLI for interpretability.

Within each recording condition, Benjamini–Hochberg FDR correction [47] was applied separately to the 12 interaction tests, 36 between-group session contrasts, and 72 within-group session contrasts, with q<0.05 as the significance threshold. Bonferroni correction was applied to the same families as a conservative sensitivity check. Complete interaction and contrast results are reported in Supplementary Tables S1–S7.

Model assumptions were assessed using raw participant-level plots, residual-versus-fitted plots, residual Q-Q plots, standardized residuals, and participant-level Cook’s distances. Models were refitted with the seven optimizer configurations implemented by allFit. No observations were removed or modified in the primary analysis. Sensitivity analyses used cell-wise 3.5-scaled-MAD winsorization and leave-one-participant-out refits of the alpha-DMN models; details are reported in Supplementary Section S8 and Figures S1–S7. Because this secondary analysis used all eligible data from the parent trial, its sample size was not determined by a prospective network-level power calculation; Supplementary Section S9 provides an approximate sensitivity benchmark.

Network-level analyses were conducted in R 4.5.2 using lme4 2.0.1, lmerTest 3.2.1, and emmeans 2.0.3.

### 3.2. Region-Level Analysis

Region-level group differences were tested across the 378 links of the 28-node triple-network matrix using a general linear model in GraphVar 2.03a, with group as the factor of interest and participant modelled as a within-subject variable. The resting-state tests had 1 and 56 degrees of freedom, and the condition-specific SEP tests had 1 and 59 degrees of freedom. GraphVar calculated an empirical *p*-value for each link by comparing its observed non-directional *F* statistic with the link-specific null distribution from 5000 non-directional permutations. Because each group test had one numerator degree of freedom, $\mathrm{sign}\left(B\right)\sqrt{F}$ was used to show effect direction in the tables. Links with p<0.05 formed connected components, and each component’s node count was compared with the permutation distribution of the maximum node count to obtain $p_{FWE}$ [48]. GraphVar’s output therefore provides two distinct quantities in this report: a link-level permutation *p*-value, used to form and describe components, and a node-count component $p_{FWE}$, used to assess the component as a whole. The *p*-values shown in the tables are the link-level values; they determine which links enter a component and are distinct from the corrected component *p*-values. Connectivity matrices were not thresholded before analysis, and the network-level LMMs provide the primary basis for inference.

The same 12 band-by-contrast comparisons (four bands by three session contrasts) were considered for each recording condition. Within each recording condition, node-count $p_{FWE}$ values were Holm-corrected across all extracted components. To examine whether conclusions depended on node count, edge extent, defined as the number of suprathreshold links in a component, was calculated from the same saved GraphVar permutation masks and compared with the permutation distribution of the maximum component extent. This edge-extent analysis was run symmetrically in both recording conditions and Holm-corrected within its own resting-state or SEP family, but it is reported only in the Supplementary Material (Supplementary Tables S13 and S21); the node-count statistic is the sole basis for component-level inference in the main text. Complete membership for the displayed components is reported in the Supplementary Material. For SEP components with a workspace node-count $p_{FWE}<0.05$, eight links were selected by smallest permutation *p*-value, then larger effect size, then ROI-pair name.

**Figure 3:**
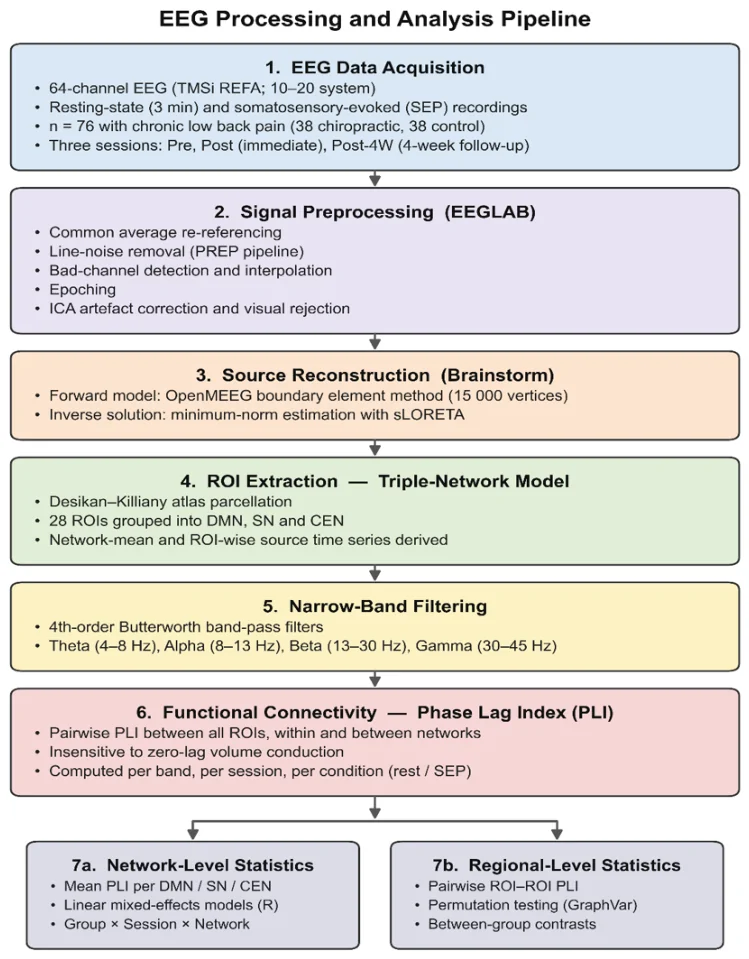
Full EEG pre-processing and analysis pipeline.

Figure 3 shows the complete preprocessing and analysis pipeline used in this study.

## 4. Results

### 4.1. Network Level Analysis

#### 4.1.1. Resting-State EEG Data

All 12 resting-state Fisher-z random-intercept LMMs were non-singular. The Group-by-Session interaction was FDR-significant in the theta DMN, SN, and CEN (all q<0.001); alpha DMN, SN, and CEN (all q<0.001); beta DMN (q=0.012); and gamma DMN (q=0.004).

In the theta band, model-estimated chiropractic-minus-control contrasts were negative in the DMN (Post: t(164.8)=−6.96; Post4W: t(164.8)=−7.27), positive in the SN (Post: t(163.4)=5.58; Post4W: t(163.4)=4.14), and positive in the CEN (Post: t(157.8)=12.93; Post4W: t(157.8)=9.10; all q<0.001).

In the alpha band, DMN connectivity was higher in the chiropractic group at Post (t(173.6)=31.64) and Post4W (t(173.6)=26.15; both q<0.001). SN connectivity was lower at Post (t(170.6)=−5.97, q<0.001) and Post4W (t(170.6)=−2.52, q=0.027), and CEN connectivity was lower at Post (t(177.0)=−7.28) and Post4W (t(177.0)=−5.59; both q<0.001).

**Figure 4:**
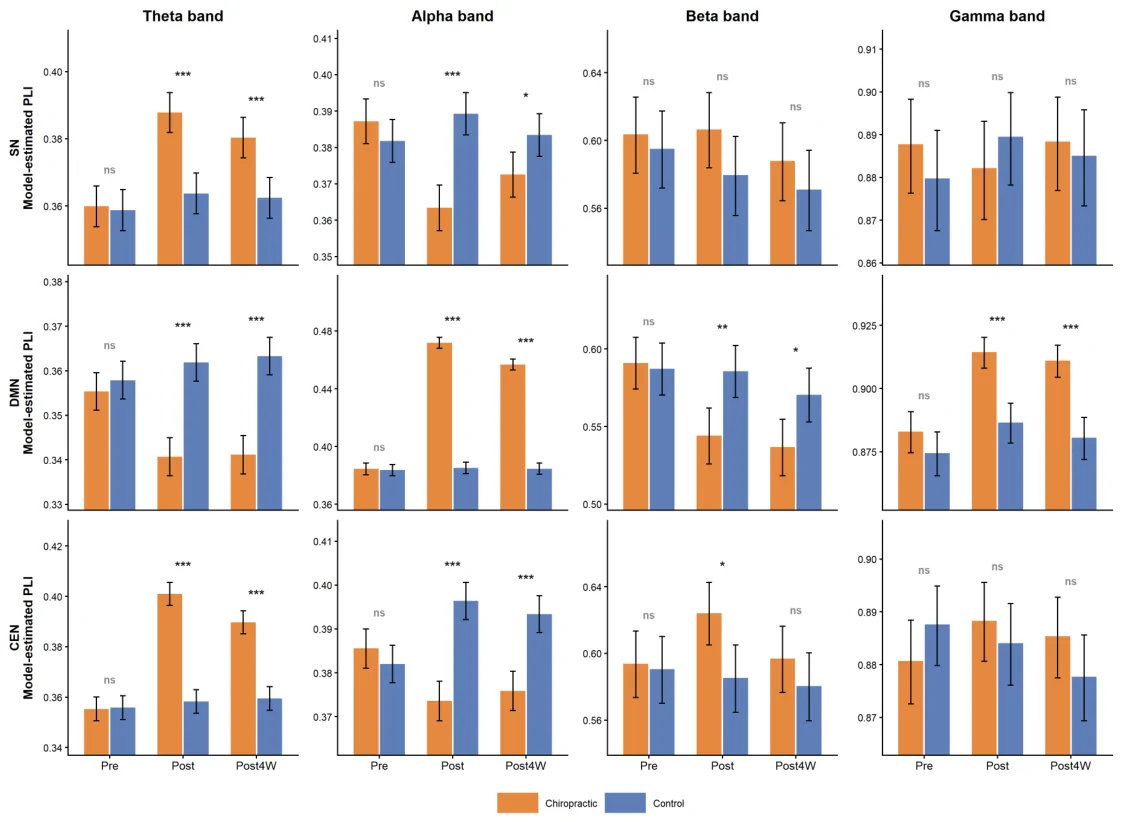
Resting-state back-transformed model-estimated marginal PLI means and 95% confidence intervals from the Fisher-z LMMs. Asterisks denote FDR-corrected model-estimated group contrasts at each session (* q<0.05, ** q<0.01, *** q<0.001; ns = not significant). Raw participant-level values are shown in Supplementary Figures S1 and S3.

In the beta band, DMN connectivity was lower at Post (t(149.3)=−3.33, q=0.003) and Post4W (t(149.3)=−2.66, q=0.020). A positive beta-CEN contrast was present at Post (t(150.4)=2.79, q=0.014), but the corresponding omnibus interaction was not significant (q=0.153); this session-specific contrast is therefore not interpreted as evidence of a differential longitudinal trajectory. Gamma-DMN connectivity was higher at Post (t(167.8)=5.59) and Post4W (t(167.8)=5.87; both q<0.001). Table 2 reports all post-treatment contrasts with q<0.05 and the corresponding interaction *q*-value. Model-estimated marginal means for all four bands are shown in Figure 4. Raw participant-level distributions and complete results are shown in Supplementary Figures S1 and S3 and Tables S1 and S7.

#### 4.1.2. Somatosensory Evoked Potentials (SEPs)

A participant-level comparison confirmed that the resting-state and SEP network-mean values are distinct: of 2115 paired participant-by-cell values none was identical across conditions, and cell-wise correlations ranged from −0.38 to 0.44 (Supplementary Figures S8 and S9). In five of the 12 SEP models (theta DMN and CEN; alpha DMN, SN, and CEN) the participant variance was estimated at zero, giving a boundary-singular fit whose Satterthwaite denominator degrees of freedom equal the residual degrees of freedom; the Group-by-Session test is non-significant under either denominator (largest interaction, alpha CEN: p=0.009 at both df=177 and df=118). The remaining seven models were non-singular.

**Figure 5:**
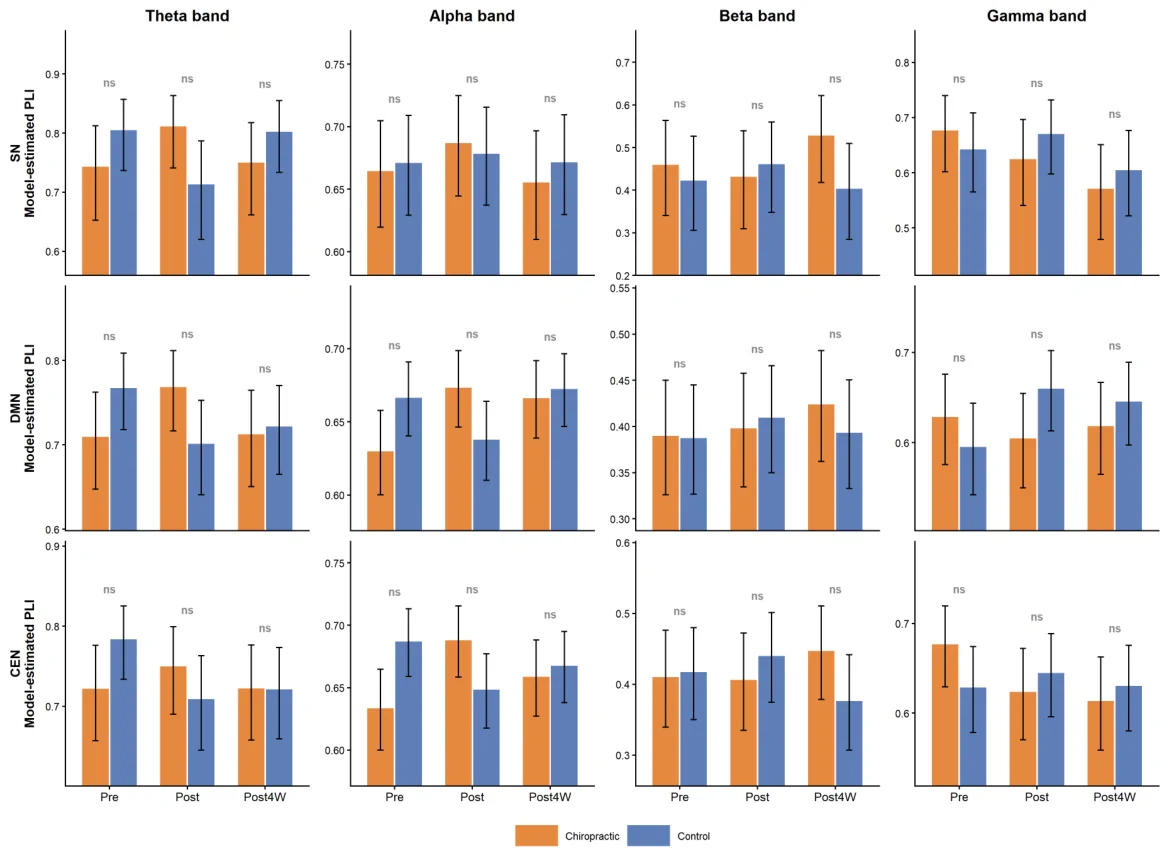
Condition-specific SEP back-transformed model-estimated marginal PLI means and 95% confidence intervals from the Fisher-z LMMs. Raw participant-level values are shown in Supplementary Figure S2.

Table 3 reports the 12 Group-by-Session interaction tests; complete between-group and within-group contrasts and raw participant-level distributions are given in Supplementary Tables S2 and S4 and Supplementary Figure S2. Model-estimated marginal means are shown in Figure 5.

In the Bonferroni sensitivity analysis, 14 of 17 FDR-significant resting-state between-group contrasts and 19 of 21 within-group contrasts remained significant; contrasts that did not meet the Bonferroni threshold are identified in Supplementary Section S8 and are treated as less robust.

### 4.2. Region-Level Analysis (Between-Group)

The region-level analysis was exploratory. The *p*-value shown for each ROI pair is its link-level GraphVar permutation *p*-value, obtained by comparing the observed non-directional *F* statistic with the null distribution for that link across 5000 permutations. Links with p<0.05 entered a connected component; these values are distinct from the corrected component *p*-values. Statistical support was evaluated for complete components using the node-count $p_{FWE}$ and its condition-specific Holm correction, which is the sole basis for component-level inference below; an equivalent post-hoc edge-extent analysis was run symmetrically in both recording conditions and is reported in the Supplementary Material only (Supplementary Tables S13 and S21). Because the supported components span 27 or 28 of the 28 nodes of the triple-network matrix, they indicate a distributed difference across the matrix as a whole and do not localise that difference to particular regions or links. Results from both recording conditions are summarised together after the link and component displays.

#### 4.2.1. Resting-State EEG Data

Pre vs. Post (Chiropractic–Control): Alpha Band

Between-group analyses evaluating pre- to post-intervention changes revealed several changes in alpha-band PLI connectivity within and across major functional networks. These links formed the only resting-state component that retained support after condition-specific correction. Regional-level PLI analyses identified increased connectivity in the chiropractic group compared to controls. The links contributing to this component included connections between the right superior parietal cortex (R.SP) and right inferior parietal cortex (R.IP; *t* = 2.98, *p* = 0.0008), the left superior frontal gyrus (L.SF) and left posterior cingulate gyrus (L.PCG; *t* = 2.77, *p* = 0.0048), and the left superior parietal cortex (L.SP) and right parahippocampal gyrus (R.PHIP; *t* = 2.43, *p* = 0.0054).

**Figure 6:**
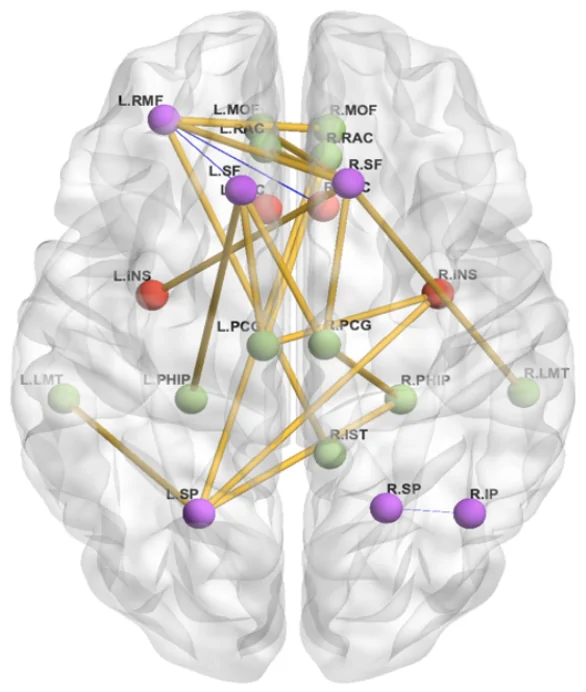
Regional-level alpha-band functional connectivity changes during resting-state EEG for the chiropractic group compared with the control group (Pre vs. Post). Nodes represent regions of interest (ROIs) from the default mode network (green), central executive network (purple), and salience network (red). Edges show the selected component links, and edge thickness reflects the strength of the change in connectivity. SP—superior parietal cortex; IP—inferior parietal cortex; SF—superior frontal gyrus; PCG—posterior cingulate gyrus; PHIP—parahippocampal gyrus; RMF—rostral middle frontal cortex; CAC—caudal anterior cingulate cortex; RAC—rostral anterior cingulate cortex; MOF—medial orbitofrontal cortex; INS—insula; IST—isthmus cingulate; PREC—precuneus.

Additional increases were observed between the right posterior cingulate gyrus (R.PCG) and right parahippocampal gyrus (R.PHIP; *t* = 2.22, *p* = 0.0074), the left rostral middle frontal cortex (L.RMF) and the left caudal anterior cingulate (L.CAC; *t* = 1.96, *p* = 0.0086), and the left superior frontal gyrus (L.SF) and the right posterior cingulate gyrus (R.PCG; *t* = 1.98, *p* = 0.0088). Connectivity enhancements were also seen between the right superior frontal gyrus (R.SF) and the left medial orbitofrontal cortex (L.MOF; *t* = 1.75, *p* = 0.0096), as well as between the right superior frontal gyrus (R.SF) and the left rostral anterior cingulate (L.RAC; *t* = 1.54, *p* = 0.0096). These findings indicate increased alpha-band connectivity involving frontal, parietal, cingulate, and parahippocampal regions in the chiropractic group compared with controls. These changes are summarised in Table 5 and visualised in Figure 6.

Pre vs. Post-4W (Chiropractic–Control): Alpha Band

For the comparison between pre-intervention and 4-week follow-up (Pre vs. Post-4W), regional-level PLI analyses identified an alpha-band component in the chiropractic group compared with the control group. The largest component did not retain support after correction ($p_{FWE}=0.4459$). The links contributing to this component included increased connectivity between the left posterior cingulate gyrus (L.PCG) and right isthmus cingulate (R.IST; *t* = 1.99, *p* = 0.0186), as well as between the right precuneus (R.PREC) and both the left posterior cingulate gyrus (L.PCG; *t* = 1.74, *p* = 0.0048) and right posterior cingulate gyrus (R.PCG; *t* = 1.66, *p* = 0.0032).

**Figure 7:**
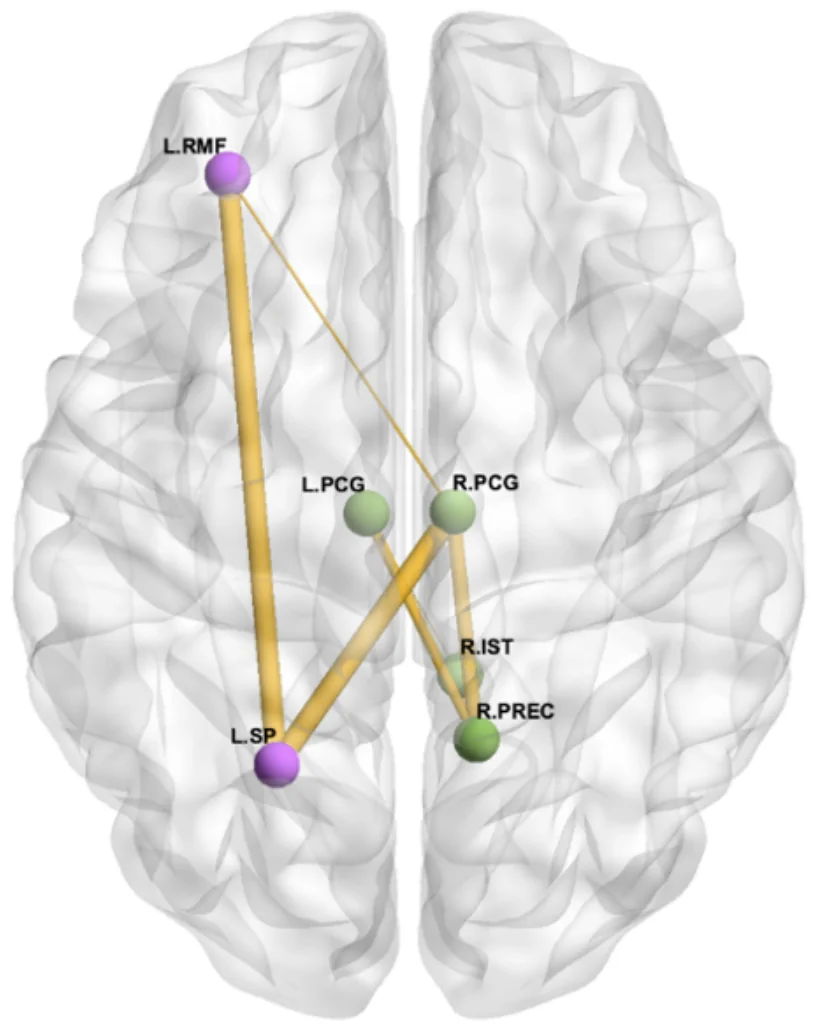
Regional-level alpha-band functional connectivity changes during resting-state EEG for the chiropractic group compared with the control group (Pre vs. Post-4W). Nodes represent regions of interest (ROIs) from the default mode network (green), central executive network (purple), and salience network (red). Edges show the selected component links. All edges reflect increased connectivity in the chiropractic group, with the strongest effects involving the left superior parietal cortex, right posterior cingulate gyrus, and left rostral middle frontal cortex. Edge thickness reflects the strength of the change in connectivity. SP—superior parietal cortex; PCG—posterior cingulate gyrus; IST—isthmus cingulate; PREC—precuneus; RMF—rostral middle frontal cortex. L—left; R—right.

Additional changes were observed between the left rostral middle frontal cortex (L.RMF) and right posterior cingulate gyrus (R.PCG; *t* = 1.61, *p* = 0.0166), the left superior parietal cortex (L.SP) and right posterior cingulate gyrus (R.PCG; *t* = 2.12, *p* = 0.0026), as well as between the left superior parietal cortex (L.SP) and left rostral middle frontal cortex (L.RMF; *t* = 2.32, *p* = 0.0064). These findings are presented in Table 6 and Figure 7.

Post vs. Post-4W (Chiropractic–Control): Alpha Band

Regional-level PLI analyses identified an alpha-band component from post-intervention to four weeks of follow-up in the chiropractic group compared with the control group. The largest component did not retain support after correction ($p_{FWE}=0.6023$). The links contributing to this component included increased synchrony between the right isthmus cingulate (R.IST) and left isthmus cingulate (L.IST; *t* = 2.44, *p* = 0.0178), right precuneus (R.PREC) and right posterior cingulate gyrus (R.PCG; *t* = 2.5, *p* = 0.015), and right superior parietal cortex (R.SP) and right inferior parietal cortex (R.IP; *t* = 2.89, *p* = 0.0022).

**Figure 8:**
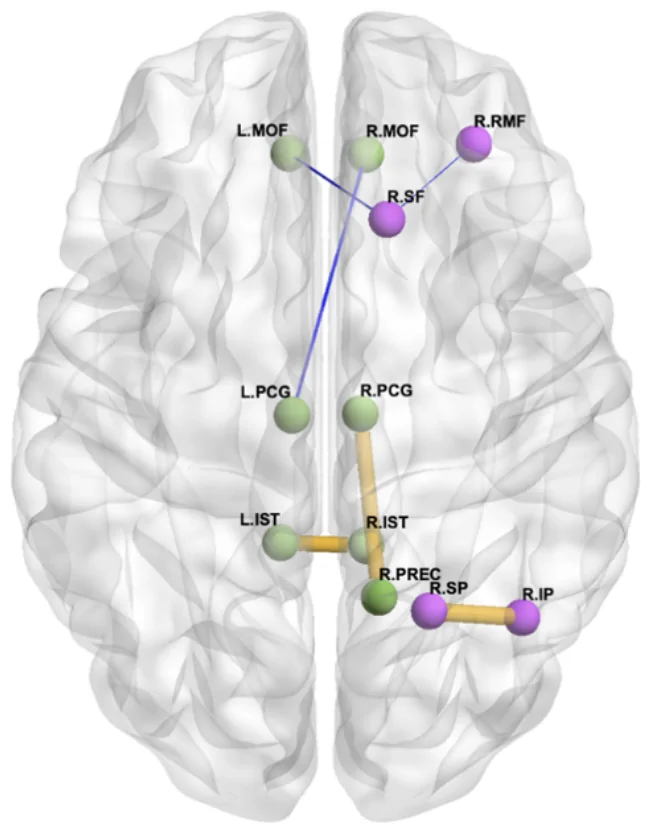
Regional-level alpha-band functional connectivity changes during resting-state EEG for the chiropractic group compared with the control group (Post vs. post-4W). Nodes represent regions of interest (ROIs) from the default mode network (green), central executive network (purple), and salience network (red). Edges show the selected component links. The figure includes both increased connectivity (e.g., R.SP—R.IP; R.IST—L.IST) and decreased connectivity (e.g., R.SF—R.RMF; L.PCG—R.MOF). IST—isthmus cingulate; SP—superior parietal cortex; IP—inferior parietal cortex; PREC— precuneus; PCG—posterior cingulate gyrus; SF—superior frontal gyrus; MOF—medial orbitofrontal cortex; RMF— rostral middle frontal cortex. L—left; R—right.

Conversely, decreased connectivity was observed between the left posterior cingulate gyrus (L.PCG) and right medial orbitofrontal cortex (R.MOF; *t* = −1.74, *p* = 0.012), right superior frontal gyrus (R.SF) and left medial orbitofrontal cortex (L.MOF; *t* = −1.98, *p* = 0.0052), and right superior frontal gyrus (R.SF) and right rostral middle frontal cortex (R.RMF; *t* = −2.12, *p* = 0.001). These findings are presented in Table 7 and Figure 8.

Pre vs. Post (Chiropractic–Control): Beta Band

Regional-level PLI analyses identified a beta-band component in the chiropractic group compared to the control group from pre- to post-intervention. The largest component did not retain support after correction ($p_{FWE}=0.4909$). The links contributing to this component included reduced connectivity between the right medial orbitofrontal cortex (R.MOF) and right caudal middle frontal cortex (R.CMF; *t* = −2.12, *p* = 0.0106), the right parahippocampal gyrus (R.PHIP) and left caudal middle frontal cortex (L.CMF; *t* = −2.09, *p* = 0.0106), and the right medial orbitofrontal cortex (R.MOF) and left caudal middle frontal cortex (L.CMF; *t* = −1.98, *p* = 0.0122).

**Figure 9:**
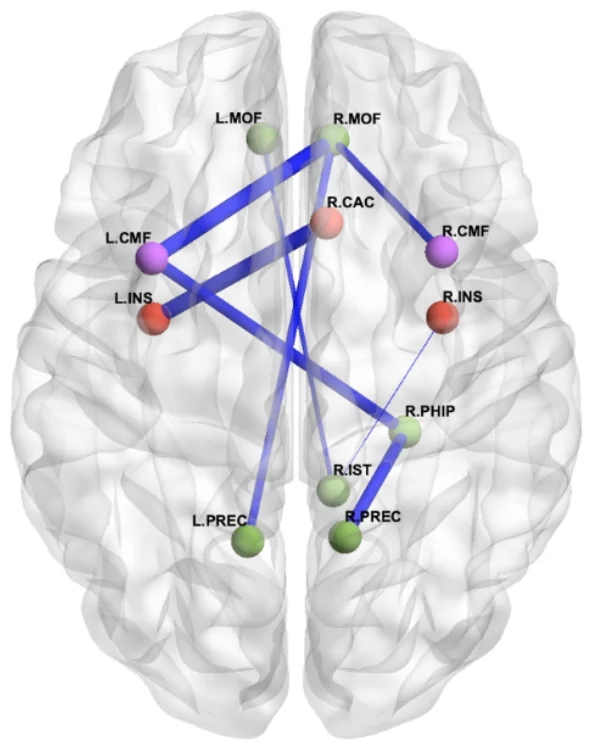
Regional-level beta-band functional connectivity changes during resting-state EEG for the chiropractic group compared with the control group (Pre vs. Post). Nodes represent regions of interest (ROIs) from the default mode network (green), central executive network (purple), and salience network (red). Edges show the selected component links. Edge thickness reflects the strength of the change in connectivity. MOF—medial orbitofrontal cortex; CMF—caudal middle frontal cortex; PHIP—parahippocampal gyrus; INS—insula; CAC—caudal anterior cingulate cortex; PREC—precuneus; IST—isthmus cingulate. L—left; R—right.

Additional decreases were observed between the left insula (L.INS) and right caudal anterior cingulate (R.CAC; *t* = −2.00, *p* = 0.0128), right precuneus (R.PREC) and right parahippocampal gyrus (R.PHIP; *t* = −1.85, *p* = 0.0138), right isthmus cingulate (R.IST) and right insula (R.INS; *t* = −1.89, *p* = 0.0156), left medial orbitofrontal cortex (L.MOF) and right isthmus cingulate (R.IST; t = −1.24, *p* = 0.016), and left precuneus (L.PREC) and right medial orbitofrontal cortex (R.MOF; *t* = −1.32, *p* = 0.0184). These findings are presented in Table 8 and Figure 9.

Pre vs. Post-4W (Chiropractic–Control): Beta Band

**Figure 10:**
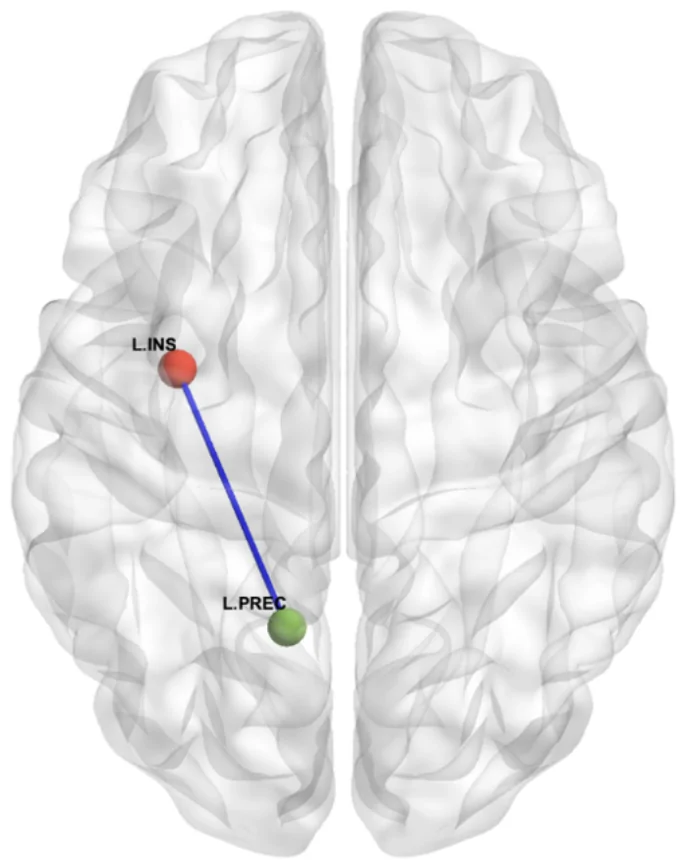
Regional-level beta-band functional connectivity changes during resting-state EEG for the chiropractic group compared with the control group (Pre vs. post-4W). The selected link shows lower connectivity between the left precuneus (L.PREC) and left insula (L.INS). PREC—precuneus; INS—insula. L— left; R—right.

Regional-level PLI analysis showed lower beta-band functional connectivity between the left precuneus (L.PREC) and left insula (L.INS) in the chiropractic group compared with the control group over the four-week follow-up period (*t* = −2.13, *p* = 0.0156). This link belonged to an 11-node component that did not retain support after correction ($p_{FWE}=0.6783$). The finding describes a localised reduction in fronto-parietal to insular connectivity at four weeks after chiropractic intervention relative to controls. It is presented in Table 9 and Figure 10.

Post vs. Post-4W (Chiropractic–Control): Beta Band

Regional-level PLI analyses identified a beta-band component from post-intervention to four weeks of follow-up in the chiropractic group compared to the control group. The largest component did not retain support after correction ($p_{FWE}=0.5345$). The links contributing to this component included reduced connectivity between the left insula (L.INS) and left caudal anterior cingulate (L.CAC; *t* = −2.12, *p* = 0.0042), between the left insula (L.INS) and right caudal anterior cingulate (R.CAC; *t* = −1.79, *p* = 0.0002), and between the left insula (L.INS) and right caudal middle frontal cortex (R.CMF; *t* = −2.11, *p* = 0.0118).

**Figure 11:**
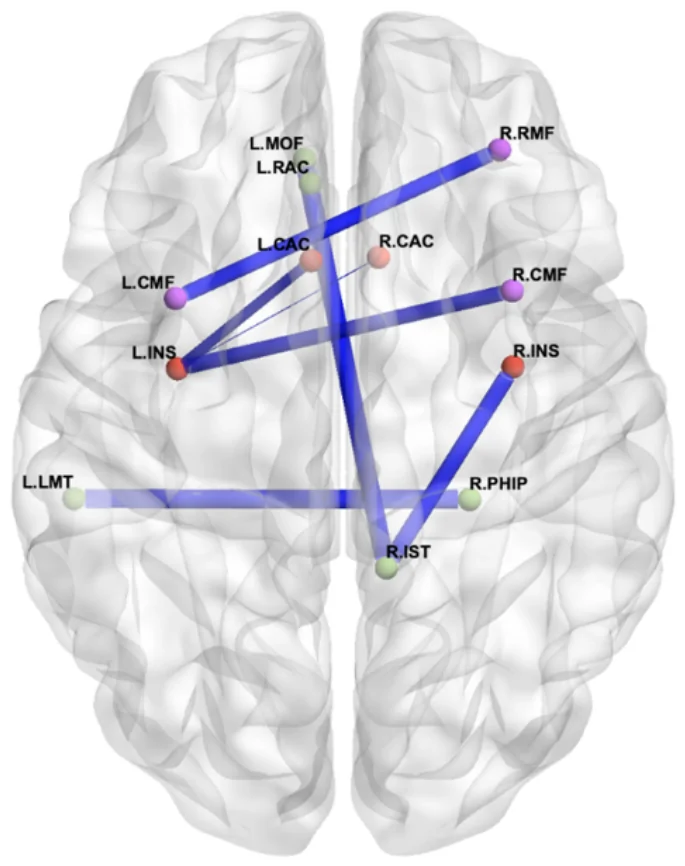
Regional-level beta-band functional connectivity changes during resting-state EEG for the chiropractic group compared with the control group (Post vs. post-4W). Nodes represent regions of interest (ROIs) from the default mode network (green), central executive network (purple), and salience network (red). Edges show the selected component links. Edge thickness reflects the strength of the change in connectivity. INS—insula; CAC—caudal anterior cingulate cortex; CMF—caudal middle frontal cortex; IST—isthmus cingulate; MOF—medial orbitofrontal cortex; PHIP—parahippocampal gyrus; MT—middle temporal gyrus; RAC—rostral anterior cingulate cortex. L—left; R—right.

Further decreases were observed between the right isthmus cingulate (R.IST) and right insula (R.INS; *t* = −2.44, *p* = 0.0106), between the left medial orbitofrontal cortex (L.MOF) and right isthmus cingulate (R.IST; *t* = −2.00, *p* = 0.0164), between the right parahippocampal gyrus (R.PHIP) and left middle temporal gyrus (L.MT; *t* = −1.98, *p* = 0.0172), as well as between the left rostral anterior cingulate (L.RAC) and right isthmus cingulate (R.IST; *t* = −1.84, *p* = 0.0116). These findings are presented in Table 10 and Figure 11.

#### 4.2.2. Somatosensory Evoked Potential (SEP) Data

**Figure 12:**
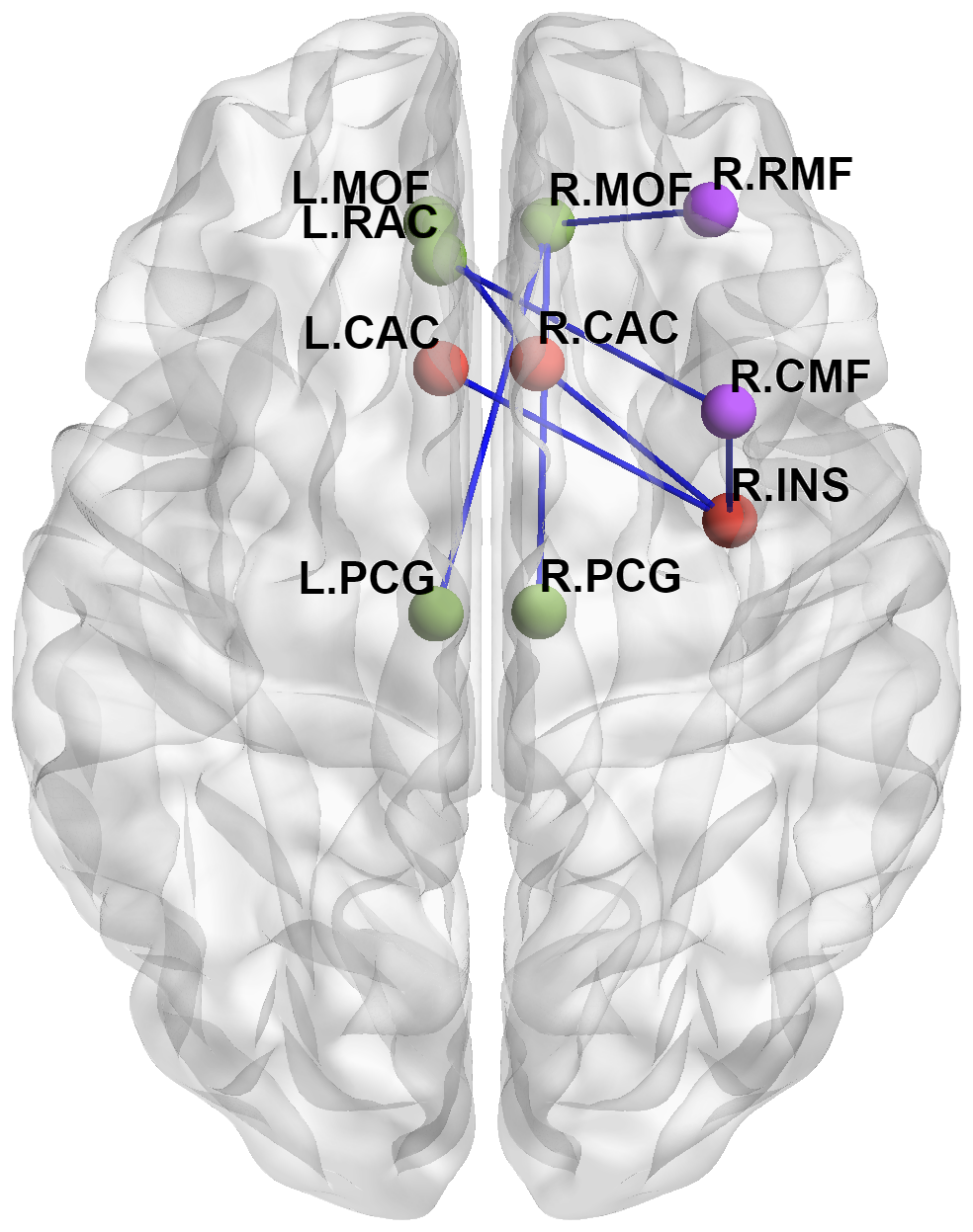
Selected links from the SEP theta-band Post versus Post-4W component. Nodes are triple-network ROIs (DMN green, CEN purple, SN red). Gold and blue show positive and negative GraphVar coefficients, and edge width reflects effect magnitude. The complete component is shown in Supplementary Figure S10.

**Figure 13:**
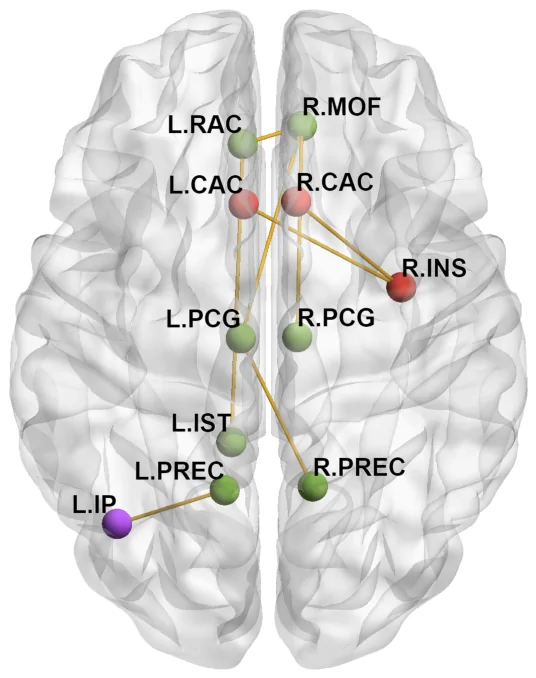
Selected links from the SEP theta-band Pre versus Post component. Node and edge conventions are as in Figure 12; the complete component is shown in Supplementary Figure S11.

**Figure 14:**
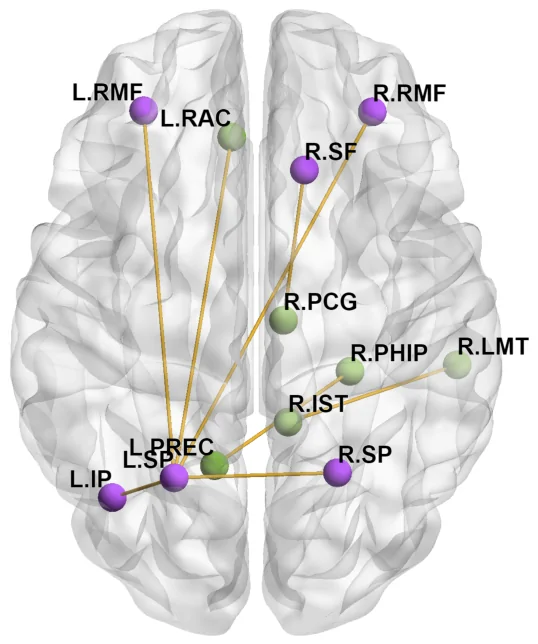
Selected links from the SEP alpha-band Pre versus Post component. Node and edge conventions are as in Figure 12; the complete component is shown in Supplementary Figure S12.

**Figure 15:**
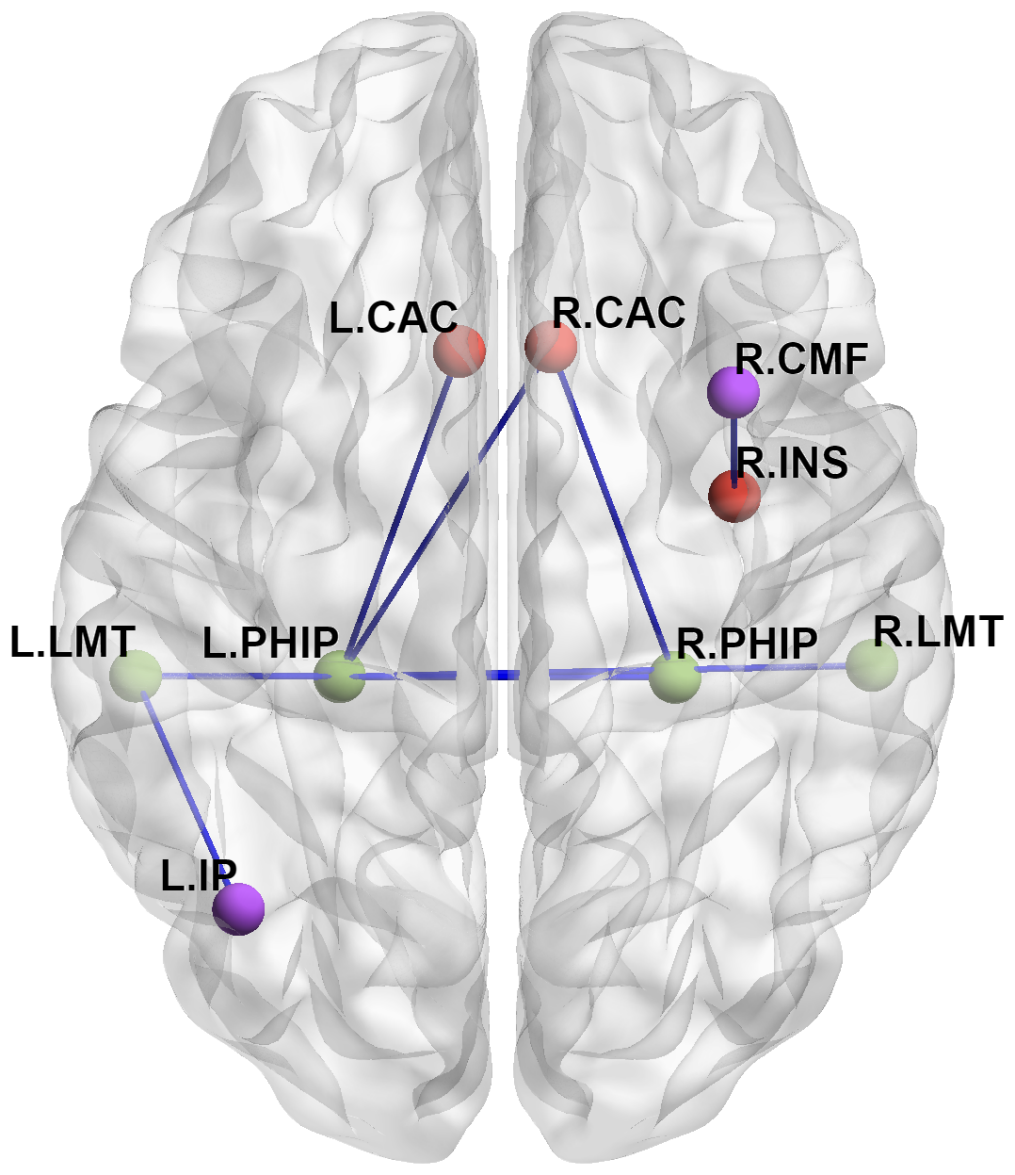
Selected links from the SEP gamma-band Pre versus Post component. Node and edge conventions are as in Figure 12; the complete component is shown in Supplementary Figure S13.

The condition-specific SEP data were analysed with the same link and component procedure used for the resting-state data. Across the 12 band-by-contrast workspaces, the largest component in four workspaces had a node-count $p_{FWE}<0.05$. Table 11, Table 12, Table 13 and Table 14 and Figure 12, Figure 13, Figure 14 and Figure 15 show eight selected links from each component. Selection was based on the smallest permutation *p*-value, then larger effect size, and then ROI-pair name. Complete member-link lists are provided in the Supplementary Material.

Post Versus Post-4W (Chiropractic–Control): Theta Band

**Table 11:** Selected links from the SEP theta-band Post versus Post-4W component (28 nodes, 86 links; node-count $p_{FWE}=0.0020$). Complete membership is provided in the Supplementary Materials.

Region 1	Region 2	*t* Value	*p* Value	Effect Size
R.MOF	R.PCG	−3.25	<0.0002	0.277
R.MOF	R.RMF	−3.91	<0.0002	0.267
L.CAC	R.INS	−3.85	<0.0002	0.266
R.CMF	R.INS	−3.81	<0.0002	0.265
R.CAC	R.INS	−3.44	0.0002	0.255
R.MOF	L.PCG	−3.08	0.0002	0.252
R.CAC	L.MOF	−4.08	0.0002	0.248
R.CMF	L.RAC	−3.24	0.0002	0.248

Pre Versus Post (Chiropractic–Control): Theta Band

**Table 12:** Selected links from the SEP theta-band Pre versus Post component (28 nodes, 88 links; node-count $p_{FWE}=0.0046$). Complete membership is provided in the Supplementary Materials.

Region 1	Region 2	*t* Value	*p* Value	Effect Size
R.MOF	R.PCG	3.95	<0.0002	0.319
L.IP	L.PREC	4.32	<0.0002	0.299
L.PCG	R.PREC	3.65	0.0004	0.296
L.CAC	R.INS	3.18	0.0004	0.287
R.CAC	R.INS	3.35	0.0004	0.282
R.MOF	L.PCG	3.48	0.0004	0.267
L.IST	L.RAC	3.32	0.0006	0.248
R.MOF	L.RAC	3.63	0.0006	0.246

Pre Versus Post (Chiropractic–Control): Alpha Band

**Table 13:** Selected links from the SEP alpha-band Pre versus Post component (27 nodes, 76 links; node-count $p_{FWE}=0.0302$). Complete membership is provided in the Supplementary Materials.

Region 1	Region 2	*t* Value	*p* Value	Effect Size
L.SP	R.SP	3.35	<0.0002	0.266
R.PCG	R.SF	3.24	0.0004	0.232
L.RMF	L.SP	3.29	0.0004	0.230
R.RMF	L.SP	3.30	0.0006	0.225
L.IP	L.SP	3.12	0.0008	0.230
L.RAC	L.SP	3.70	0.0010	0.215
R.PHIP	L.PREC	3.04	0.0012	0.221
R.IST	R.MT	3.38	0.0012	0.219

Pre Versus Post (Chiropractic–Control): Gamma Band

**Table 14:** Selected links from the SEP gamma-band Pre versus Post component (28 nodes, 70 links; node-count $p_{FWE}=0.0098$). Complete membership is provided in the Supplementary Materials.

Region 1	Region 2	*t* Value	*p* Value	Effect Size
R.MT	L.PHIP	−3.57	<0.0002	0.344
R.CMF	R.INS	−3.22	0.0004	0.295
L.IP	L.MT	−2.87	0.0006	0.289
R.CAC	R.PHIP	−3.49	0.0006	0.287
L.CAC	L.PHIP	−3.20	0.0006	0.285
R.CAC	L.PHIP	−3.22	0.0016	0.277
L.MT	R.PHIP	−3.24	0.0016	0.275
L.PHIP	R.PHIP	−2.94	0.0018	0.278

Condition-Wide Correction and Post-Hoc Sensitivity Analysis

Table 15 summarises the additional correction results for both recording conditions. The 12 resting-state comparisons produced 14 components, all from the alpha and beta bands, since no theta or gamma comparison contained a link at p<0.05. The 12 SEP comparisons produced 24 components. Holm correction was applied separately to these component families. Resting-state alpha Pre versus Post retained node-count support ($p_{FWE}=0.0030$, Holm p=0.0420). SEP theta Post versus Post-4W also retained node-count support ($p_{FWE}=0.0020$, Holm p=0.048); the other three displayed SEP components did not and are not interpreted as supported. Both supported components span 27 or 28 of the 28 nodes, so neither localises the difference to particular regions. The post-hoc edge-extent sensitivity analysis, run symmetrically in both recording conditions, is reported in Supplementary Tables S13 and S21. All region-level findings remain exploratory.

## 5. Discussion

Before interpreting the findings, it is important to distinguish between more and less robust results. Alpha-band effects, which showed large between-group differences (d > 1.0) that were robust in the resting-state condition across both post-intervention time points, represent the most replicable findings of this study. Theta, beta, and gamma effects were generally smaller (d < 0.8 at several time points), less consistent across networks, and should be treated as preliminary until replicated. All findings are from an exploratory secondary analysis and should be interpreted accordingly.

The present study found evidence consistent with frequency-specific reorganisation within large-scale brain networks following chiropractic care in individuals with chronic low back pain. At both immediate and four-week post-intervention time points, participants receiving chiropractic adjustments showed increased alpha-band functional connectivity within the DMN, alongside concurrent reductions in connectivity within the SN and CEN. Because the network-level models summarised connectivity within each network rather than coupling between them, this pattern is compatible with a rebalancing of interactions across the DMN, SN, and CEN, but that interpretation was not directly tested and remains provisional.

These findings extend our previous work, which demonstrated increased alpha activity within DMN regions and modulation of early sensorimotor processing following chiropractic care [12]. They are consistent with central nervous system involvement, although the mechanism by which chiropractic care affected network connectivity remains to be established. Rather than proving a specific causal pathway, the observed pattern is compatible with a shift away from salience-driven, hypervigilant processing toward a more balanced network configuration.

Chronic pain is increasingly conceptualised as a disorder of large-scale brain network organisation rather than a purely peripheral nociceptive phenomenon. In particular, the “triple network model” proposes that dysfunction within and between the DMN, SN, and CEN underlies many of the cognitive, emotional, and attentional disturbances observed in chronic pain populations [14,15,16,17]. Neuroimaging studies have consistently demonstrated abnormal connectivity between these networks, particularly increased coupling between the DMN and SN, which may contribute to persistent attentional bias toward pain and maladaptive self-referential processing [14,15]. Within this framework, the increased alpha connectivity observed within the default mode network following chiropractic care, together with concurrent reductions in within-network salience and executive connectivity, may reflect increased alpha–band integration between the default mode and central executive networks and reduced beta-band salience network coupling.

### 5.1. Resting State EEG Source Localisation Within and Between the DMN, CEN and SN

#### 5.1.1. Alpha Band

Resting-state alpha-band findings were among the most striking and consistent results of the present study. The observed increase in alpha-band connectivity within the DMN suggests improved cortical integration and coordination within regions involved in self-referential processing [14,22]. In addition to these DMN-specific effects, the present results demonstrate concurrent reductions in alpha connectivity within the SN and CEN, indicating a broader reorganisation of alpha-mediated functional connectivity across the triple-network system. Importantly, these changes may reflect increased alpha–band integration between the DMN and CEN. These findings extend previous analyses from this trial, which demonstrated increased alpha activity within the DMN following chiropractic care [12]. Together, the present results indicate that chiropractic care is associated not simply with a change in DMN activity, but with a broader reorganisation of alpha-mediated functional connectivity across the triple-network system. This broader network-level interpretation is important because the DMN, SN, and CEN are not pain-specific systems, but transdiagnostic networks involved in self-referential processing, salience detection, executive control, emotional regulation, and adaptive behavioural responding [19,21]. Importantly, the participants in the present study demonstrated clinically meaningful improvements not only in pain, but also in mood and fatigue, suggesting that the observed network-level changes may reflect a more general reorganisation of brain function across multiple domains rather than a pain-specific effect.

Alpha oscillations regulate cortical inhibition and the gating of sensory input, and their enhancement reflects more efficient sensory filtering and integration of internal–external states [49]. This interpretation is important because alpha activity is increasingly understood as an active mechanism of functional inhibition and sensory gating, helping to suppress task-irrelevant processing and regulate the routing of information across distributed brain networks [49]. Chronic pain disrupts these regulatory mechanisms, resulting in DMN-SN coupling that perpetuates attentional bias toward pain and emotional distress [19]. The reduction in SN connectivity observed following chiropractic care, together with enhanced alpha connectivity, supports the interpretation that regular spinal adjustments recalibrate salience attribution processes and restore adaptive network communication. Within this framework, the increased alpha connectivity observed in the DMN, together with concurrent reductions in connectivity within the CEN, may reflect a reorganisation of interactions between internally directed processing and executive control systems, while the reduced alpha connectivity in the SN indicates reduced salience attribution to pain-related or otherwise behaviourally irrelevant internal signals. In other words, the present findings are consistent with a shift away from a chronic pain-related state in which salience processing dominates cognition and self-referential processing toward a more balanced pattern of large-scale network interaction [49].

Alpha oscillations play a key role in regulating cortical excitability and gating sensory information, and alterations in alpha activity have been widely reported in chronic pain populations [50,51,52,53]. Reduced alpha power and connectivity are often associated with impaired sensory filtering and increased cortical hyperexcitability. The pattern of alpha-band connectivity observed in the present study, characterised by increased connectivity within the default mode network alongside concurrent reductions in salience and executive network connectivity, therefore likely reflects a reorganisation of coordination across cortical regions involved in self-referential processing, cognitive control, and sensorimotor integration. These findings align with previous work demonstrating that chiropractic adjustments influence cortical processing and sensorimotor integration, including changes in prefrontal cortex function and altered somatosensory processing following spinal adjustments [7,8,9,12]. Interestingly, the observed increases in alpha-band connectivity are consistent with enhanced sensory gating following chiropractic care, reflecting improved filtering of incoming sensory information and more effective suppression of irrelevant or noise-driven inputs. This account rests on the resting-state alpha findings and on prior work using dual somatosensory evoked potentials; the somatosensory-evoked connectivity measured in the present study did not itself provide converging evidence, for the reasons given in the Somatosensory Evoked Potential Connectivity subsection. One plausible route involves thalamocortical projections, but EEG cannot directly assess subcortical activity and cortico-cortical mechanisms are equally consistent with these data. This interpretation is consistent with previous studies recording dual SEPs, which have demonstrated that chiropractic care enhances early sensorimotor gating [54]. Importantly, these changes occurred alongside the observed improvements in pain in the present cohort, suggesting that enhanced sensory gating may contribute to the clinical benefits of chiropractic care. This interpretation aligns with evidence that chronic pain is associated with impaired sensory gating and excessive propagation of nociceptive and non-relevant signals to cortical processing systems [55] and therefore may reflect a shift toward more regulated and selective cortical processing. Importantly, this mechanism may also be relevant to the observed improvements in anxiety, mood, fatigue, and sleep, because inefficient sensory filtering and persistent salience-driven processing can increase cognitive and affective load, disrupt rest–wake regulation, and reduce the brain’s capacity to return to a stable baseline state [19]. Consistent with this, alterations in alpha activity and sensory gating have been reported in both anxiety and depressive disorders, where reduced inhibitory control and increased cortical excitability are associated with heightened emotional reactivity and impaired regulation of internal states [56,57]. Furthermore, dysregulation of large-scale networks, particularly involving the DMN, SN, and CEN, has been implicated in the pathophysiology of both anxiety and depression, where abnormal salience attribution and disrupted executive control contribute to persistent negative affect and maladaptive cognitive processing [19].

The present findings are also consistent with the broader literature describing EEG alterations in chronic pain populations. Alpha abnormalities have been repeatedly reported in chronic pain populations, although the direction and localisation of effects vary across studies and conditions [50,51,52,53,58,59]. Reduced alpha power, particularly in frontal and insular regions, has been associated with chronic pain intensity [60], and Ref. [61] further showed that reduced alpha is independently related to both pain intensity and sleep deficits. Systematic reviews likewise identify alpha abnormalities as a recurring feature of chronic pain while emphasising heterogeneity across chronic pain populations and recording paradigms [59,62]. Thus, the current findings should not be interpreted as evidence of a single universal alpha biomarker, but rather as support for the view that alpha-band dynamics are centrally involved in the altered sensory, affective, and cognitive processing that characterises chronic pain populations [59,60,61,63].

Importantly, these alpha abnormalities have also been linked to disruptions in large-scale brain network interactions, particularly involving the DMN and SN. In this context, the present findings are particularly relevant to previous work demonstrating pathological coupling between the DMN and pain-related regions such as the insula in chronic pain populations [14,22]. While these studies have primarily identified altered network coupling at a systems level, the present findings extend this work by demonstrating frequency-specific changes in network dynamics, characterised by reduced SN connectivity and increased stability of DMN activity. This pattern may reflect a shift away from pathological DMN–SN coupling toward a more regulated and differentiated network state.

The present results are particularly interesting because the alpha-band changes were network-specific, with distinct patterns of increased and decreased connectivity observed across the triple-network system. Increased alpha connectivity in the DMN may reflect altered self-referential processing and a reduced tendency toward maladaptive internally focused pain-related rumination, reduced alpha connectivity in the CEN may reflect decreased internally driven executive activity and a shift toward more efficient or less effortful top-down regulation and cognitive control [14,15,16,17,20]. Conversely, reduced alpha connectivity in the SN suggests that pain-related or interoceptive signals became less behaviourally dominant or less persistently prioritised following chiropractic care. This network-specific pattern is compatible with the triple-network model of chronic pain, in which abnormal interactions between the DMN, SN, and CEN contribute to persistent pain, attentional bias, affective dysregulation, and impaired cognitive control [17,20].

Peng and colleagues [53] proposed that pain-related alpha activity reflects an integration of sensory-discriminative, affective-motivational, and cognitive-modulative influences rather than a purely sensory pain signal. That framework fits the present findings well. The observed alpha redistribution across the DMN, SN, and CEN therefore reflects a broader change in how internal bodily signals are processed, evaluated, and regulated. This helps explain why the alpha changes in this cohort occurred alongside previously reported improvements not only in pain, but also in sleep, mood, fatigue, and overall quality of life [62].

#### 5.1.2. Beta Band

Changes within the beta frequency range further support the hypothesis of improved cortical regulation. Beta-band desynchronization, particularly across prefrontal and anterior cingulate regions, is commonly interpreted as reduced cortical rigidity and heightened readiness for adaptive updating of motor and cognitive processes [64,65,66]. Beta oscillations are commonly associated with the maintenance of the current sensorimotor state and top-down control of motor and cognitive processes [66]. In the present study, beta-band connectivity decreased within the default mode network and across prefrontal, cingulate, and insular regions, alongside a reduction in salience network coupling, while a transient increase in central executive network connectivity was observed immediately following the intervention. These results align with earlier research demonstrating improved prefrontal efficiency and reduced N30 amplitudes following chiropractic care, reflecting more effective sensorimotor integration and enhanced efficiency of central sensorimotor processing [7,8,9,12]. The coordinated modulation of beta and alpha activity, therefore, may reflect complementary processes of reduced cortical rigidity and enhanced functional coordination across large-scale brain networks following chiropractic spinal adjustments.

#### 5.1.3. Theta and Gamma Bands

Furthermore, changes within the theta and gamma bands indicate an additional layer of neuroplastic adaptation. Theta-band activity has been linked to cognitive control, emotional regulation, and coordination between large-scale brain networks, particularly involving the prefrontal cortex [67,68]. Theta synchronisation across the CEN may underlie enhanced cognitive control and emotion regulation [67,68]. In the current study, increased theta-band connectivity within executive networks may reflect enhanced engagement of prefrontal regulatory systems involved in sensory and emotional processing. Reduced frontocingulate theta connectivity during emotional regulation has previously been reported in major depressive disorder [68]. Accordingly, the observed increases in theta-band connectivity may be consistent with enhanced prefrontal regulatory capacity and a shift toward more effective top-down control of emotional processes. Notably, these neurophysiological changes were accompanied by a statistically significant and clinically meaningful reduction in depression and anxiety. This is clinically relevant because altered interactions among the DMN, SN, and CEN have been implicated in depression and anxiety, where disrupted balance between internally focused processing, salience attribution, and executive regulation is thought to contribute to rumination, hypervigilance, and impaired emotional control [19]. These findings are consistent with the hypothesis that chiropractic care, through improving proprioceptive accuracy and afferent signalling, influences the way the brain integrates sensory information, thereby enhancing prefrontal regulation of emotional and cognitive processes.

Gamma oscillations support local cortical binding and the integration of distributed neural information [69]. Increased gamma connectivity within posterior DMN regions observed in the present study could reflect strengthened binding and awareness of somatosensory input [69], and may indicate enhanced coordination and integration of internally generated information with incoming sensory signals. This may reflect the processing of altered proprioceptive input arising from the spine, potentially contributing to the updating of internal body representations and recalibration of sensorimotor integration processes.

The persistence of these network patterns four weeks post-intervention, together with previously reported improvements in mood, fatigue, and sleep [12], supports the interpretation that chiropractic care may facilitate durable, systems-level plasticity through modulation of afferent input and network-level processing. From a mechanistic perspective, spinal adjustments deliver a rapid mechanical stimulus to paraspinal tissues rich in mechanoreceptors and muscle spindles, generating a substantial burst of proprioceptive input to the central nervous system [1]. This afferent input is known to influence processing within spinal, cerebellar, and cortical structures involved in movement control and sensory integration [1]. Such changes in sensory input may contribute to recalibration of network dynamics across the DMN, SN, and CEN, particularly in individuals with chronic pain, where these systems are already dysregulated.

Taken together, the frequency-specific findings reported here indicate a reorganisation of connectivity within the three networks. Increased alpha-band connectivity within the DMN, alongside reduced beta-band coupling within salience-related circuits, form a pattern consistent with chiropractic care influencing both within- and between-network interactions across the triple-network system. Given that chronic pain has been associated with disrupted coordination between these networks, including increased salience dominance and pathological coupling with the DMN [14,22], the present pattern may reflect a shift toward more balanced and flexible network dynamics. Specifically, the combined modulation of alpha and beta activity is consistent with enhanced inhibitory control and sensory gating, alongside reduced cortical rigidity and diminished salience-driven processing. Importantly, these findings should not be interpreted as pain-specific only, but as consistent with a broader reorganisation of large-scale brain networks involved in affective, cognitive, autonomic, sleep-related, and energy-regulation processes, consistent with the observed improvements in pain, depression, anxiety, fatigue, sleep, and quality of life in this cohort.

### 5.2. Somatosensory Evoked Potential Connectivity

The network-level SEP analysis produced no significant Group-by-Session interaction in any of the twelve band-by-network models (Table 3). This should not be read as evidence that somatosensory processing was unaffected, nor as a failure to replicate the resting-state findings, because the two conditions do not measure the same quantity. Resting-state PLI was estimated from several minutes of ongoing activity, whereas SEP PLI was derived from the averaged evoked response across a 250 ms interval, which removes activity that is not phase-locked to the stimulus and provides far fewer independent phase observations. The SEP condition therefore had limited sensitivity to the sustained network reorganisation detected at rest, and the two conditions should not be treated as a replication test of one another.

At the SEP region level, four components had a within-comparison node-count $p_{FWE}<0.05$, and after condition-specific Holm correction only the theta-band Post-to-Post-4W component remained. That component spans all 28 nodes of the triple-network matrix, so it indicates a distributed difference rather than a regionally specific one. The SEP matrices were derived from the averaged evoked response over an interval containing fewer than two theta cycles. This result is therefore preliminary, and a target for future work on cortical processing of somatosensory input following care.

### 5.3. Regional Connectivity Patterns

The regional-level analyses describe the composition of the supported components rather than localising the network-level findings; the supported resting-state component spans 27 of the 28 nodes in the matrix. In the alpha band, the links contributing most strongly to the component involved posterior DMN nodes, particularly connections between the posterior cingulate gyrus, parahippocampal gyrus, and precuneus. These regions form the core of the DMN’s memory and self-referential processing subsystem [70]. The posterior cingulate is a central hub for self-referential processing and shows altered connectivity in chronic pain populations [22]. The increased alpha synchrony between R.PCG and R.PHIP at the post-intervention time point is consistent with previous findings from this dataset showing enhanced parahippocampal-posterior cingulate coupling following adjustments [12]; this region pair lies within the resting-state alpha-band component reported above, and statistical support applies to that component rather than to the pair.

Connections between CEN and DMN regions also showed exploratory changes within the supported resting-state component. The links contributing to this component included connections between the superior frontal gyrus and the posterior cingulate, and between the rostral middle frontal cortex and the caudal anterior cingulate. Statistical support applies to the component rather than to these links, and improved cross-network communication between executive and default mode regions remains a preliminary interpretation. In the chronic pain literature, weakened CEN-DMN coupling has been associated with impaired cognitive control over pain-related rumination [14,22]. The connectivity pattern is therefore consistent with improved coordination between executive and self-referential processing systems, but it should be interpreted as a candidate mechanism requiring direct mediation testing rather than evidence that chiropractic care shifted attentional resources away from pain focus.

The beta-band regional findings predominantly showed decreased connectivity, particularly involving connections between the insula and anterior cingulate regions, which are key nodes of the salience network. The decrease in beta connectivity between L.INS and both L.CAC and R.CAC from post-intervention to four-week follow-up is one of the links forming that component, which did not retain support after correction; heightened insular-cingulate beta connectivity has been associated with chronic pain-related hypervigilance [27]. This reduction persisted beyond the immediate post-intervention period, which may reflect a progressive normalisation of salience processing over the course of care.

The four SEP components are shown in the same link/component format as the resting-state results (Table 11, Table 12, Table 13 and Table 14; Figure 12, Figure 13, Figure 14 and Figure 15). Each spans 27 or 28 of the 28 nodes, so the links displayed in those tables and figures illustrate component composition rather than regionally specific effects. Because three of the four components did not retain support after condition-specific Holm correction, and because the short SEP epoch contains few theta cycles, the regional interpretation remains exploratory.

### 5.4. Integration with Clinical Outcomes

The triple network connectivity changes observed here occurred alongside the clinical improvements reported in the parent study [12]. In that trial, the chiropractic group showed clinically meaningful improvements in the PROMIS-29 domains of anxiety, depression, fatigue, pain intensity, and pain interference over the four-week intervention period, alongside increased light sleep duration, as measured by Fitbit actigraphy. No formal correlation or mediation analyses were conducted between connectivity changes and clinical outcomes, so the following observations are correlational and speculative.

The increased alpha DMN connectivity is consistent with the improvements in depression and anxiety, given that reduced DMN alpha coherence is a consistent finding in mood disorders [71], and the medial orbitofrontal cortex (a key DMN node that showed enhanced regional connectivity in our data) has reduced functional connectivity in depressed individuals [72]. Reduced SN connectivity is consistent with decreased salience-driven processing, as salience network hyperactivation is thought to drive the attentional amplification of nociceptive input that characterises chronic pain [14].

The improvements in fatigue and light sleep duration are consistent with the beta-band desynchronisation observed within the CEN. Sustained beta activity is metabolically costly and has been associated with cortical fatigue in chronic pain populations [61]. The reduction of beta CEN connectivity, particularly in prefrontal-sensorimotor circuits, is consistent with decreased neural effort required to maintain executive control, potentially freeing resources for restorative sleep processes. The parent study reported increased light sleep (N2) duration in the chiropractic group, and light sleep is an important stage for sleep spindle generation and memory consolidation [73]. These associations are correlational and require formal mediation analysis before causal conclusions can be drawn. Future studies should include pre-planned correlation and mediation analyses to directly test whether specific connectivity changes account for improvements in each clinical domain.

### 5.5. Molecular Evidence Supporting Cortical Plasticity Mechanisms

Recent transcriptomic data from a related chronic non-specific LBP cohort provide convergent but indirect context for the cortical reorganisation observed here. Sannes et al [74] profiled peripheral blood mononuclear cell gene expression in chronic non specific LBP patients and found that, when analysis was restricted to non-specific low back pain, the most enriched biological processes were axon guidance, regulation of trans-synaptic signalling, modulation of chemical synaptic transmission, and neuron projection guidance. KEGG pathway analysis confirmed enrichment of cholinergic synapse, glutamatergic synapse, and neuroactive ligand–receptor interaction pathways. These findings indicate that chronic spinal dysfunction may be associated with alterations in molecular pathways involved in synaptic plasticity and neural connectivity. At a systems level, such processes would be expected to influence the organisation and strength of functional interactions between large-scale brain networks, including the default mode, salience, and executive control systems examined in the present study.

These molecular observations derive from a separate cohort and cannot be directly linked to the present EEG findings. The two datasets are broadly consistent with a model in which altered afferent signalling is associated with plasticity across multiple levels of the nervous system, but this convergence is speculative. Future studies combining transcriptomic profiling with source-level EEG connectivity within the same participants will be required to directly test these cross-level relationships.

### 5.6. Comparison with Other Functional Connectivity Studies

These findings are broadly consistent with the existing functional connectivity literature across conditions involving large-scale network dysfunction. fMRI studies have repeatedly reported disrupted DMN connectivity in CLBP populations, with reduced medial prefrontal-posterior cingulate connectivity being the most replicated finding [22]. Direct comparison between fMRI and EEG connectivity metrics is limited by methodological differences, but the direction of our findings, increased alpha-DMN connectivity following intervention, is consistent with a shift towards more stable and coordinated DMN dynamics. This pattern parallels recent fMRI work in fibromyalgia showing that cognitive behavioural therapy reduced posterior cingulate cortex connectivity to salience and somatomotor regions alongside reductions in pain catastrophizing [75], suggesting that distinct non-pharmacological interventions may converge on similar DMN-salience network reconfigurations in chronic pain populations.

PLI-based EEG studies remain relatively scarce, which makes the present findings difficult to compare directly. However, Waterstone et al. [10] reported increased alpha-band DMN connectivity in stroke survivors following a single session of chiropractic care, which is consistent with both the immediate and sustained DMN alpha increases observed here.

The triple network framework applied in this study follows the model proposed by De Ridder et al. [14], who argued that chronic pain arises from aberrant interactions between the DMN, SN, and CEN. Our data are compatible with this framework: the opposing connectivity changes between the DMN (increased alpha) and SN (decreased alpha) are consistent with a rebalancing between internally directed processing and salience detection. This pattern aligns with models proposing that effective interventions modulate interactions between these networks towards more balanced and flexible dynamics [14].

Taken together, these findings suggest that chiropractic spinal adjustments are associated with frequency- and network-specific functional reorganisation across the DMN, SN, and CEN that is evident immediately after the first session and again after four weeks of care. The resting-state findings, together with the distributed component identified at the region level, are consistent with a model in which modulation of paraspinal proprioceptive input is associated with distributed cortical plasticity across large-scale networks involved in affective, cognitive, and sensorimotor processing. Future studies should use formal mediation analyses to test whether the observed connectivity changes statistically mediate the clinical improvements reported in the parent study, and whether baseline connectivity patterns predict treatment response.

### 5.7. Strengths and Future Research Directions

This study has several strengths. It draws on data from a parallel-group randomised controlled trial, which provides a controlled comparison that most prior EEG work on spinal manipulation has lacked. The analysis was source-localised rather than sensor-level, and it used the phase lag index, which reduces sensitivity to volume conduction. The main alpha-band effects were large and consistent in the resting-state condition at both post-intervention time points, and the region-level analysis identified a distributed alpha-band network in the same condition. Reporting of effect sizes and complete results, including non-significant contrasts, supports transparent interpretation.

Several directions follow from the present work. First, the study should be replicated in an adequately powered, pre-registered trial that includes an active or sham comparator, standardised and tracked usual care, and prospective recording of medication use, so that non-specific contextual effects and pharmacological confounding can be controlled. Second, dynamic, time-resolved connectivity analyses (for example, time-varying DMN-SN and CEN-SN coupling) may capture the flexible network switching proposed by the triple-network model more directly than the static, session-level connectivity reported here. We did not conduct dynamic analyses in the present study because the recordings and this secondary analysis were not designed for them. Third, pre-planned correlation and mediation analyses linking connectivity changes to clinical outcomes are needed to test whether the network changes account for the improvements reported in the parent study. These were not performed here because the analysis was exploratory and the required individual-level clinical data were outside its scope. Fourth, source reconstruction using individual MRI-based head models, together with forward-model sensitivity analyses, would reduce the geometric error introduced by the generic head model used here. Finally, future work should extend the framework to other large-scale systems, including affective, interoceptive, and autonomic regulatory networks, to better characterise the broader neural mechanisms underlying multi-domain clinical outcomes.

### 5.8. Limitations

Several limitations should be considered when interpreting these findings. First, this study represents a secondary analysis of data collected for the parent trial [12], and the triple network connectivity hypothesis was not pre-registered. While the analysis was motivated by observations in the parent study’s Supplementary File S2, which identified the need to explore salience network changes, the exploratory nature of the analysis means that the findings should be considered exploratory and hypothesis-generating.

Second, although 76 participants were randomised, the network-level models included 61 participants in the alpha band and 58 in the theta, beta, and gamma bands. The parent trial’s sample size was determined for its clinical outcomes, not for this secondary frequency-by-network analysis. Sensitivity to smaller effects may therefore be limited, particularly in the theta and gamma bands. In addition, several FDR-significant contrasts did not meet the corresponding Bonferroni threshold and should be treated as less robust. These considerations reinforce the exploratory interpretation of the network-level findings.

Third, as with all manual intervention trials, blinding of the chiropractor who provided care was not possible. Although participants were blinded to group allocation, the absence of a sham intervention control limits the ability to attribute changes specifically to specific components of the intervention rather than to non-specific effects such as therapeutic expectation or the general effects of clinical interaction. A related limitation is the imbalance in therapeutic contact between the groups. The chiropractic group attended 12 sessions over four weeks, whereas the control group received a single initial session plus usual care. By the four-week assessment, the two groups had therefore experienced very different amounts of therapeutic attention, physical contact, and repeated clinical interaction. Expectancy and Hawthorne effects are likely to have been stronger in the chiropractic group, and this makes it difficult to attribute the observed differences to the HVLA adjustments specifically rather than to these non-specific contextual effects. Both groups were asked about their perceived allocation to assess blinding [12], but the contact imbalance remains an important constraint on causal interpretation.

Fourth, usual care was not standardised or tracked during the trial period. Concurrent treatments, including medication use, physiotherapy, exercise, psychological therapy, and changes in sleep, were not systematically recorded in either group. This is an important limitation because CNS-active agents, in particular analgesics and sedatives, are known to influence EEG oscillations in the alpha and beta bands. With a modest analysed sample per group, a chance imbalance in even a few participants starting or changing a CNS-active medication during the study could have contributed to the observed EEG differences independently of chiropractic care. Because these data were not collected, we cannot rule out this source of confounding, and it should be recorded and reported in future trials.

Fifth, EEG has inherent limitations in capturing signals from deep brain structures. Activity in subcortical regions, including the cerebellum, basal ganglia, and brainstem, which are involved in sensorimotor, pain, affective, and autonomic regulation, could not be directly assessed. The limited spatial resolution and signal attenuation with depth reduce the detectability of cerebellar electrophysiological activity in scalp EEG [76]. Future studies combining EEG with neuroimaging modalities capable of resolving deeper brain structures, such as functional magnetic resonance imaging, may therefore provide further insights into the mechanisms underlying the neuroplastic effects of chiropractic care.

A further limitation concerns the source reconstruction and connectivity estimation. Source activity was reconstructed with sLORETA using a generic, non-individual head model (Colin27) rather than head models built from each participant’s own structural MRI. A generic model introduces geometric error into the forward solution, which can bias source localisation and the resulting connectivity estimates. We used the phase lag index precisely because it is insensitive to zero-lag volume conduction, but PLI reduces rather than eliminates the effects of source leakage and signal mixing, and residual leakage can still occur, particularly for nearby regions and under a generic head model. The network- and region-level connectivity values should therefore be interpreted as relative changes between groups and sessions rather than as exact anatomical connections. Individual MRI-based head models and forward-model sensitivity analyses were beyond the scope of this secondary analysis, and we identify them as a priority for confirmatory work.

A further consideration concerns the scope of the region-level analysis. Family-wise error was controlled at the level of the graph component rather than the individual link, so the analysis identifies subnetworks in which connectivity differs between groups and characterises the region pairs that constitute them. Individual link *p*-values describe component membership, whereas statistical support was evaluated for the component as a whole. Our inferences are drawn from the network-level analyses. In addition, band-specific connectivity matrices were unavailable for three control participants in the theta, beta, and gamma bands, which is why the analysed sample is 32 in the alpha band and 29 in the remaining bands. This reflects the availability of derived files from the parent trial rather than any band-specific artefact-rejection step, since band decomposition was applied to the same cleaned recordings. The intermediate records that would identify why these matrices were unavailable are no longer accessible. Future analyses should archive preprocessing logs and analysis configurations alongside the derived connectivity matrices.

Finally, SEP connectivity was estimated from the averaged evoked response across a 512-sample interval spanning −100 to +149 ms. Averaging the retained epochs before estimation removes activity that is not phase-locked to the stimulus, so the SEP matrices describe phase-lag structure within the evoked waveform rather than ongoing coupling. The interval also contains fewer than two theta cycles. Phase-based estimates in the theta band, and to a lesser extent the low alpha band, are therefore weakly determined in the SEP condition, and the SEP theta findings should be treated as preliminary. The SEP region-level results are therefore treated as preliminary throughout.

Sixth, while we report clinical outcomes from the parent study for contextual interpretation, no formal correlation or mediation analyses were conducted between the connectivity changes and clinical improvements. Whether the observed network changes directly account for the improvements in pain, mood, and sleep therefore remains to be tested. Future studies should include pre-planned correlation and mediation analyses to test these relationships and to determine whether specific network changes predict improvements across different functional domains.

Finally, although the regional analysis included links both within and between networks, the network-level models summarised each network separately and did not estimate time-resolved inter-network coupling. Future work should examine explicit between-network aggregate PLI and time-resolved DMN–SN and CEN–SN coupling, which may be more informative for understanding the triple-network dynamics proposed by De Ridder et al. (2022) [14]. In addition, given the observed improvements in mood, fatigue, and sleep in this cohort, future studies should also examine functional connectivity within other large-scale systems, including affective and autonomic/interoceptive networks, to better characterise the broader neural mechanisms underlying multi-domain clinical outcomes.

### 5.9. Conclusions

This exploratory secondary analysis found that chiropractic care is associated with frequency- and network-specific changes in functional connectivity across large-scale brain networks. Increased alpha-band connectivity within the DMN, alongside concurrent reductions in alpha-band connectivity within the SN and CEN during resting state, is consistent with a rebalancing of intrinsic brain network dynamics following spinal adjustments. These within-network changes form a pattern consistent with more coordinated interactions between networks involved in internally directed cognition, executive control, and salience detection; these between-network effects were not directly tested. These patterns were sustained over the four-week intervention period in the resting-state condition. At the region level, a distributed alpha-band network showed increased resting-state connectivity in the chiropractic group after care. No SEP network-level interaction survived multiple-comparison correction; the corresponding component-level somatosensory evoked findings were computed from epoch-averaged responses over a 250 ms interval and remain preliminary and hypothesis-generating. Given the exploratory nature of this analysis and the design limitations detailed in the Limitations subsection, these findings should be interpreted as hypothesis-generating and require replication in adequately powered, pre-registered trials.

These findings are consistent with the interpretation that spinal adjustments may influence central neural processing through modulation of afferent sensory input, particularly proprioceptive signals from the spine. The network-level changes are also consistent with the improvements in pain, mood, fatigue, and sleep reported in the parent study, but the present analysis did not test mediation, so whether the EEG changes account for those clinical outcomes remains to be determined.

Taken together, the present findings provide preliminary, exploratory evidence that chiropractic care is associated with frequency-specific reorganisation of large-scale brain network dynamics. We advance this as a hypothesis rather than a demonstrated mechanism. Whether these connectivity changes underlie the improvements in pain, mood, fatigue, sleep, or quality of life reported in the parent study remains to be established. Testing that link will require adequately powered, pre-registered trials with pre-planned mediation analyses.

## Figures and Tables

**Table 4:** Regions of interest forming the triple-network system.

Brain Regions	Abbreviations	Network
caudalanteriorcingulate (left)	L.CAC	SN
caudalanteriorcingulate (right)	R.CAC	SN
caudalmiddlefrontal (left)	L.CMF	CEN
caudalmiddlefrontal (right)	R.CMF	CEN
inferiorparietal (left)	L.IP	DMN
inferiorparietal (right)	R.IP	DMN
insula (left)	L.INS	SN
insula (right)	R.INS	SN
isthmuscingulate (left)	L.IST	DMN
isthmuscingulate (right)	R.IST	DMN
medialorbitofrontal (left)	L.MOF	DMN
medialorbitofrontal (right)	R.MOF	DMN
middletemporal (left)	L.MT	DMN
middletemporal (right)	R.MT	DMN
parahippocampal (left)	L.PHIP	DMN
parahippocampal (right)	R.PHIP	DMN
posteriorcingulate (left)	L.PCG	DMN
posteriorcingulate (right)	R.PCG	DMN
precuneus (left)	L.PREC	DMN
precuneus (right)	R.PREC	DMN
rostralanteriorcingulate (left)	L.RAC	DMN
rostralanteriorcingulate (right)	R.RAC	DMN
rostralmiddlefrontal (left)	L.RMF	CEN
rostralmiddlefrontal (right)	R.RMF	CEN
superiorfrontal (left)	L.SF	CEN
superiorfrontal (right)	R.SF	CEN
superiorparietal (left)	L.SP	CEN
superiorparietal (right)	R.SP	CEN

**Table 5:** Selected links from the resting-state alpha-band Pre versus Post component (27 nodes, 42 links; node-count $p_{FWE}=0.0030$). Complete membership is provided in the Supplementary Materials.

Region 1	Region 1	*t* Value	*p* Value	Effect Size
R.SP	R.IP	2.98	0.0008	0.10359
L.SF	L.PCG	2.77	0.0048	0.089967
L.SP	R.PHIP	2.43	0.0054	0.087839
R.PCG	R.PHIP	2.22	0.0074	0.082588
L.RMF	L.CAC	1.96	0.0086	0.08273
L.SF	R.PCG	1.98	0.0088	0.084291
R.SF	L.MOF	1.75	0.0096	0.085001
R.SF	L.RAC	1.54	0.0096	0.091103

**Table 6:** Selected links from the resting-state alpha-band Pre versus Post-4W component (16 nodes, 20 links; node-count $p_{FWE}=0.4459$). Complete membership is provided in the Supplementary Materials.

Region 1	Region 1	*t* Value	*p* Value	Effect Size
L.PCG	R.IST	1.99	0.0186	0.074358
R.PREC	L.PCG	1.74	0.0048	0.083582
R.PREC	R.PCG	1.66	0.0032	0.08869
L.RMF	R.PCG	1.61	0.0166	0.076345
L.SP	R.PCG	2.12	0.0026	0.097346
L.SP	L.RMF	2.32	0.0064	0.095927

**Table 7:** Selected links from the resting-state alpha-band Post versus Post-4W component (13 nodes, 12 links; node-count $p_{FWE}=0.6023$). Complete membership is provided in the Supplementary Materials.

Region 1	Region 1	*t* Value	*p* Value	Effect Size
R.IST	L.IST	2.44	0.0178	0.074926
L.PCG	R.MOF	−1.74	0.012	−0.07507
R.PREC	R.PCG	2.5	0.015	0.075493
R.SF	L.MOF	−1.98	0.0052	−0.08585
R.SF	R.RMF	−2.12	0.001	−0.10274
R.SP	R.IP	2.89	0.0022	0.099475

**Table 8:** Selected links from the resting-state beta-band Pre versus Post component (15 nodes, 18 links; node-count $p_{FWE}=0.4909$). Complete membership is provided in the Supplementary Materials.

Region 1	Region 1	*t* Value	*p* Value	Effect Size
R.MOF	R.CMF	−2.12	0.0106	−0.10416
R.PHIP	L.CMF	−2.09	0.0106	−0.10331
R.MOF	L.CMF	−1.98	0.0122	−0.1016
L.INS	R.CAC	−2	0.0128	−0.10175
R.PREC	R.PHIP	−1.85	0.0138	−0.10302
R.IST	R.INS	−1.89	0.0156	−0.10572
L.MOF	R.IST	−1.24	0.016	−0.10473
L.PREC	R.MOF	−1.32	0.0184	−0.10359

**Table 9:** Selected link from the resting-state beta-band Pre versus Post-4W component (11 nodes, 10 links; node-count $p_{FWE}=0.6783$). Complete membership is provided in the Supplementary Materials.

Region 1	Region 1	*t* Value	*p* Value	Effect Size
L.PREC	L.INS	−2.13	0.0156	−0.09834

**Table 10:** Selected links from the resting-state beta-band Post versus Post-4W component (14 nodes, 13 links; node-count $p_{FWE}=0.5345$). Complete membership is provided in the Supplementary Materials.

Region 1	Region 1	*t* Value	*p* Value	Effect Size
L.INS	L.CAC	−2.12	0.0042	−0.11977
L.INS	R.CAC	−1.79	0.0002	−0.14474
L.INS	R.CMF	−2.11	0.0118	−0.10841
R.IST	R.INS	−2.44	0.0106	−0.10402
L.MOF	R.IST	−2	0.0164	−0.09891
R.PHIP	L.MT	−1.98	0.0172	−0.09877
L.RAC	R.IST	−1.84	0.0116	−0.10487

**Table 15:** Condition-wide correction summary for components with node-count $p_{FWE}<0.05$. Holm values were calculated separately for the resting-state and SEP component families. The post-hoc edge-extent sensitivity analysis is reported in Supplementary Tables S13 and S21.

Condition	Band	Contrast	Nodes	Links	Node $p_{\mathrm{FWE}}$	Node Holm	Support
Resting	Alpha	Pre vs. Post	27	42	0.0030	0.0420	Retained
SEP	Theta	Post vs. Post-4W	28	86	0.0020	0.048	Retained
SEP	Theta	Pre vs. Post	28	88	0.0046	0.106	Not retained
SEP	Alpha	Pre vs. Post	27	76	0.0302	0.634	Not retained
SEP	Gamma	Pre vs. Post	28	70	0.0098	0.216	Not retained

## Data Availability

Data may be made available by the corresponding author upon reasonable request, subject to approval by the relevant ethics committee.

## Supplementary Materials

The following supporting information can be downloaded at: https://www.mdpi.com/article/10.3390/brainsci16080818/s1, S1: Resting-state: all model-estimated between-group contrasts; S2: Somatosensory evoked potentials: all model-estimated betweengroup contrasts; S3: Resting-state: all model-estimated within-group contrasts; S4: Somatosensory evoked potentials: all model-estimated withingroup contrasts; S5: Baseline resting-state network-mean PLI; S6: Baseline SEP network-mean PLI; S7: Omnibus Group-by-Session tests; S8: Model validation and sensitivity analyses; S9: Sample-size sensitivity benchmark; S10: Participant-level comparison of resting-state and SEP connectivity; S11: Region-level components (resting-state); S12: Region-level components (SEP).

## Abbreviations

The following abbreviations are used in this manuscript:

BEM	Boundary Element Method
CEN	Central Executive Network
CLBP	Chronic Low Back Pain
DMN	Default Mode Network
EEG	Electroencephalography
FDR	False Discovery Rate
fMRI	Functional Magnetic Resonance Imaging
HVLA	High-Velocity Low-Amplitude
ICA	Independent Component Analysis
LMM	Linear Mixed-Effects Model
PLI	Phase Lag Index
PROMIS-29	Patient-Reported Outcomes Measurement Information System–29 item
RCT	Randomised Controlled Trial
REML	Restricted Maximum Likelihood
ROI	Region of Interest
SD	Standard Deviation
SE	Standard Error
SEP	Somatosensory Evoked Potential
sLORETA	Standardised Low-Resolution Brain Electromagnetic Tomography
SN	Salience Network
VAS	Visual Analogue Scale
