# Supplementary material for: Chiropractic Care Is Associated with Frequency-Specific Reorganisation of Triple-Network Brain Dynamics: A Source-Localised EEG Study
Source: Brain Sci. 2026 Jul 31;16(8):818. doi: 10.3390/brainsci16080818 (PMC13510510; doi:10.3390/brainsci16080818)
Supplement: Supplementary file 1 [file brainsci-16-00818-s001.zip › brainsci-4396946-supplementary.pdf]

Supplementary Material

Complete network-level results for the manuscript *Chiropractic Care Is Associated with Frequency-Specific Reorganisation of Triple-Network Brain Dynamics*. For each recording condition, frequency band, and network, Fisher-z-transformed network-mean PLI was analysed using a linear mixed-effects model with fixed effects of Group, Session, and Group by Session and a random intercept for the unique participant identifier formed from group and participant number. Each row entering a model was one participant-session network mean; ROI pairs, epochs, and time windows were averaged before modelling and were not independent rows. Reported t-statistics, Satterthwaite degrees of freedom, and p-values come directly from the fitted LMM. The descriptive model-standardised effect d_{LMM} is the Fisher-z contrast divided by the residual standard deviation from the same model and was not used to calculate the t-statistic. Benjamini–Hochberg FDR correction was applied separately to the 12 Group-by-Session interaction tests, 36 between-group session contrasts, and 72 within-group session contrasts within each recording condition. Significance: *** < .001, ** < .01, * < .05, ns = not significant.

S1. Resting-state: all model-estimated between-group contrasts

Table S1: Resting-state EEG: all model-estimated between-group contrasts (chiropractic minus control), including non-significant comparisons. Estimates and SEs are on the Fisher-z scale. Fractional df values are Satterthwaite denominator degrees of freedom based on the repeated participant-session responses and fitted variance components in the corresponding LMM; they are not counts of independent participants.

Band	Network	Session	N	z estimate	SE	df	t	q	d_{LMM}	Sig
Theta	DMN	Pre	29/29	-0.0029	0.0035	164.8	-0.83	.522	-0.23	ns
Theta	DMN	Post	29/29	-0.0242	0.0035	164.8	-6.96	< .001	-1.93	***
Theta	DMN	Post4W	29/29	-0.0253	0.0035	164.8	-7.27	< .001	-2.01	***
Theta	SN	Pre	29/29	+0.0013	0.0050	163.4	0.26	.838	+0.07	ns
Theta	SN	Post	29/29	+0.0281	0.0050	163.4	5.58	< .001	+1.56	***
Theta	SN	Post4W	29/29	+0.0208	0.0050	163.4	4.14	< .001	+1.16	***
Theta	CEN	Pre	29/29	-0.0006	0.0039	157.8	-0.15	.884	-0.04	ns
Theta	CEN	Post	29/29	+0.0499	0.0039	157.8	12.93	< .001	+3.75	***
Theta	CEN	Post4W	29/29	+0.0351	0.0039	157.8	9.10	< .001	+2.64	***
Alpha	DMN	Pre	29/32	+0.0009	0.0034	173.6	0.28	.838	+0.08	ns
Alpha	DMN	Post	29/32	+0.1062	0.0034	173.6	31.64	< .001	+8.54	***
Alpha	DMN	Post4W	29/32	+0.0878	0.0034	173.6	26.15	< .001	+7.06	***
Alpha	SN	Pre	29/32	+0.0063	0.0051	170.6	1.25	.352	+0.34	ns
Alpha	SN	Post	29/32	-0.0302	0.0051	170.6	-5.97	< .001	-1.65	***
Alpha	SN	Post4W	29/32	-0.0127	0.0051	170.6	-2.52	.027	-0.69	*
Alpha	CEN	Pre	29/32	+0.0042	0.0037	177.0	1.13	.388	+0.29	ns
Alpha	CEN	Post	29/32	-0.0268	0.0037	177.0	-7.28	< .001	-1.88	***
Alpha	CEN	Post4W	29/32	-0.0206	0.0037	177.0	-5.59	< .001	-1.44	***
Beta	DMN	Pre	29/29	+0.0057	0.0183	149.3	0.31	.838	+0.09	ns
Beta	DMN	Post	29/29	-0.0610	0.0183	149.3	-3.33	.003	-1.01	**
Beta	DMN	Post4W	29/29	-0.0487	0.0183	149.3	-2.66	.020	-0.81	*
Beta	SN	Pre	29/29	+0.0133	0.0252	158.8	0.53	.720	+0.15	ns
Beta	SN	Post	29/29	+0.0417	0.0252	158.8	1.65	.201	+0.48	ns
Beta	SN	Post4W	29/29	+0.0255	0.0252	158.8	1.01	.452	+0.29	ns
Beta	CEN	Pre	29/29	+0.0052	0.0220	150.4	0.23	.838	+0.07	ns
Beta	CEN	Post	29/29	+0.0612	0.0220	150.4	2.79	.014	+0.84	*
Beta	CEN	Post4W	29/29	+0.0252	0.0220	150.4	1.15	.388	+0.35	ns

Band	Network	Session	N	z estimate	SE	df	t	q	d_{LMM}	Sig
Gamma	DMN	Pre	29/29	+0.0374	0.0264	167.8	1.41	.302	+0.38	ns
Gamma	DMN	Post	29/29	+0.1477	0.0264	167.8	5.59	< .001	+1.49	***
Gamma	DMN	Post4W	29/29	+0.1552	0.0264	167.8	5.87	< .001	+1.56	***
Gamma	SN	Pre	29/29	+0.0365	0.0371	165.8	0.98	.452	+0.27	ns
Gamma	SN	Post	29/29	-0.0343	0.0371	165.8	-0.93	.475	-0.25	ns
Gamma	SN	Post4W	29/29	+0.0155	0.0371	165.8	0.42	.787	+0.11	ns
Gamma	CEN	Pre	29/29	-0.0315	0.0253	167.4	-1.24	.352	-0.33	ns
Gamma	CEN	Post	29/29	+0.0198	0.0253	167.4	0.78	.539	+0.21	ns
Gamma	CEN	Post4W	29/29	+0.0342	0.0253	167.4	1.35	.321	+0.36	ns

S2. Somatosensory evoked potentials: all model-estimated between-group contrasts

Table S2: SEP EEG: all model-estimated between-group contrasts (chiropractic minus control), including non-significant comparisons. Estimates and SEs are on the Fisher-z scale. Fractional df values are Satterthwaite denominator degrees of freedom based on the repeated participant-session responses and fitted variance components in the corresponding LMM; they are not counts of independent participants.

Band	Network	Session	N	z estimate	SE	df	t	q	d_{LMM}	Sig
Theta	DMN	Pre	29/32	-0.1274	0.0808	177.0	-1.58	.419	-0.40	ns
Theta	DMN	Post	29/32	+0.1465	0.0808	177.0	1.81	.419	+0.47	ns
Theta	DMN	Post4W	29/32	-0.0196	0.0808	177.0	-0.24	.898	-0.06	ns
Theta	SN	Pre	29/32	-0.1552	0.1240	176.3	-1.25	.588	-0.33	ns
Theta	SN	Post	29/32	+0.2364	0.1240	176.3	1.91	.419	+0.50	ns
Theta	SN	Post4W	29/32	-0.1319	0.1240	176.3	-1.06	.742	-0.28	ns
Theta	CEN	Pre	29/32	-0.1427	0.0868	177.0	-1.64	.419	-0.42	ns
Theta	CEN	Post	29/32	+0.0867	0.0868	177.0	1.00	.767	+0.26	ns
Theta	CEN	Post4W	29/32	+0.0027	0.0868	177.0	0.03	.975	+0.01	ns
Alpha	DMN	Pre	29/32	-0.0633	0.0334	177.0	-1.90	.419	-0.49	ns
Alpha	DMN	Post	29/32	+0.0624	0.0334	177.0	1.87	.419	+0.48	ns
Alpha	DMN	Post4W	29/32	-0.0114	0.0334	177.0	-0.34	.898	-0.09	ns
Alpha	SN	Pre	29/32	-0.0119	0.0533	177.0	-0.22	.898	-0.06	ns
Alpha	SN	Post	29/32	+0.0160	0.0533	177.0	0.30	.898	+0.08	ns
Alpha	SN	Post4W	29/32	-0.0288	0.0533	177.0	-0.54	.884	-0.14	ns
Alpha	CEN	Pre	29/32	-0.0952	0.0378	177.0	-2.52	.419	-0.65	ns
Alpha	CEN	Post	29/32	+0.0717	0.0378	177.0	1.89	.419	+0.49	ns
Alpha	CEN	Post4W	29/32	-0.0157	0.0378	177.0	-0.41	.898	-0.11	ns
Beta	DMN	Pre	29/32	+0.0028	0.0510	133.8	0.05	.975	+0.02	ns
Beta	DMN	Post	29/32	-0.0139	0.0510	133.8	-0.27	.898	-0.09	ns
Beta	DMN	Post4W	29/32	+0.0369	0.0510	133.8	0.72	.850	+0.24	ns
Beta	SN	Pre	29/32	+0.0459	0.0990	159.0	0.46	.891	+0.14	ns
Beta	SN	Post	29/32	-0.0364	0.0990	159.0	-0.37	.898	-0.11	ns
Beta	SN	Post4W	29/32	+0.1599	0.0990	159.0	1.62	.419	+0.47	ns
Beta	CEN	Pre	29/32	-0.0085	0.0575	126.0	-0.15	.935	-0.05	ns
Beta	CEN	Post	29/32	-0.0414	0.0575	126.0	-0.72	.850	-0.25	ns
Beta	CEN	Post4W	29/32	+0.0849	0.0575	126.0	1.47	.452	+0.51	ns
Gamma	DMN	Pre	29/32	+0.0529	0.0578	165.9	0.92	.805	+0.26	ns
Gamma	DMN	Post	29/32	-0.0918	0.0578	165.9	-1.59	.419	-0.45	ns
Gamma	DMN	Post4W	29/32	-0.0452	0.0578	165.9	-0.78	.850	-0.22	ns
Gamma	SN	Pre	29/32	+0.0609	0.0892	175.4	0.68	.850	+0.18	ns

Band	Network	Session	N	z estimate	SE	df	t	q	d_{LMM}	Sig
Gamma	SN	Post	29/32	-0.0785	0.0892	175.4	-0.88	.805	-0.23	ns
Gamma	SN	Post4W	29/32	-0.0514	0.0892	175.4	-0.58	.884	-0.15	ns
Gamma	CEN	Pre	29/32	+0.0842	0.0583	165.5	1.44	.452	+0.41	ns
Gamma	CEN	Post	29/32	-0.0352	0.0583	165.5	-0.60	.884	-0.17	ns
Gamma	CEN	Post4W	29/32	-0.0277	0.0583	165.5	-0.48	.891	-0.14	ns

S3. Resting-state: all model-estimated within-group contrasts

Table S3: Resting-state EEG: all model-estimated within-group session contrasts, including non-significant comparisons. Estimates and SEs are on the Fisher-z scale. Fractional df values are Satterthwaite denominator degrees of freedom based on the repeated participant-session responses and fitted variance components in the corresponding LMM; they are not counts of independent participants.

Band	Network	Group	Contrast	N	z estimate	SE	df	t	q	d_{LMM}	Sig
Theta	DMN	Chiro	Pre vs Post	29	+0.0167	0.0033	112.0	5.06	< .001	+1.33	***
Theta	DMN	Chiro	Pre vs Post4W	29	+0.0162	0.0033	112.0	4.91	< .001	+1.29	***
Theta	DMN	Chiro	Post vs Post4W	29	-0.0005	0.0033	112.0	-0.15	.895	-0.04	ns
Theta	DMN	Control	Pre vs Post	29	-0.0046	0.0033	112.0	-1.40	.349	-0.37	ns
Theta	DMN	Control	Pre vs Post4W	29	-0.0062	0.0033	112.0	-1.88	.174	-0.49	ns
Theta	DMN	Control	Post vs Post4W	29	-0.0016	0.0033	112.0	-0.48	.760	-0.13	ns
Theta	SN	Chiro	Pre vs Post	29	-0.0325	0.0047	112.0	-6.88	< .001	-1.81	***
Theta	SN	Chiro	Pre vs Post4W	29	-0.0237	0.0047	112.0	-5.02	< .001	-1.32	***
Theta	SN	Chiro	Post vs Post4W	29	+0.0088	0.0047	112.0	1.86	.174	+0.49	ns
Theta	SN	Control	Pre vs Post	29	-0.0057	0.0047	112.0	-1.21	.435	-0.32	ns
Theta	SN	Control	Pre vs Post4W	29	-0.0042	0.0047	112.0	-0.89	.605	-0.23	ns
Theta	SN	Control	Post vs Post4W	29	+0.0015	0.0047	112.0	0.33	.811	+0.09	ns
Theta	CEN	Chiro	Pre vs Post	29	-0.0533	0.0035	112.0	-15.26	< .001	-4.01	***
Theta	CEN	Chiro	Pre vs Post4W	29	-0.0399	0.0035	112.0	-11.42	< .001	-3.00	***
Theta	CEN	Chiro	Post vs Post4W	29	+0.0134	0.0035	112.0	3.83	< .001	+1.01	***
Theta	CEN	Control	Pre vs Post	29	-0.0028	0.0035	112.0	-0.81	.620	-0.21	ns
Theta	CEN	Control	Pre vs Post4W	29	-0.0042	0.0035	112.0	-1.21	.435	-0.32	ns
Theta	CEN	Control	Post vs Post4W	29	-0.0014	0.0035	112.0	-0.40	.786	-0.10	ns
Alpha	DMN	Chiro	Pre vs Post	29	-0.1071	0.0033	118.0	-32.80	< .001	-8.61	***
Alpha	DMN	Chiro	Pre vs Post4W	29	-0.0879	0.0033	118.0	-26.93	< .001	-7.07	***
Alpha	DMN	Chiro	Post vs Post4W	29	+0.0191	0.0033	118.0	5.86	< .001	+1.54	***
Alpha	DMN	Control	Pre vs Post	32	-0.0018	0.0031	118.0	-0.58	.707	-0.14	ns
Alpha	DMN	Control	Pre vs Post4W	32	-0.0011	0.0031	118.0	-0.35	.806	-0.09	ns
Alpha	DMN	Control	Post vs Post4W	32	+0.0007	0.0031	118.0	0.23	.866	+0.06	ns
Alpha	SN	Chiro	Pre vs Post	29	+0.0277	0.0048	118.0	5.75	< .001	+1.51	***
Alpha	SN	Chiro	Pre vs Post4W	29	+0.0171	0.0048	118.0	3.55	.002	+0.93	**
Alpha	SN	Chiro	Post vs Post4W	29	-0.0106	0.0048	118.0	-2.20	.094	-0.58	ns
Alpha	SN	Control	Pre vs Post	32	-0.0088	0.0046	118.0	-1.93	.163	-0.48	ns
Alpha	SN	Control	Pre vs Post4W	32	-0.0019	0.0046	118.0	-0.42	.784	-0.11	ns
Alpha	SN	Control	Post vs Post4W	32	+0.0069	0.0046	118.0	1.50	.316	+0.38	ns
Alpha	CEN	Chiro	Pre vs Post	29	+0.0140	0.0037	118.0	3.73	.001	+0.98	**
Alpha	CEN	Chiro	Pre vs Post4W	29	+0.0113	0.0037	118.0	3.02	.011	+0.79	*
Alpha	CEN	Chiro	Post vs Post4W	29	-0.0027	0.0037	118.0	-0.71	.661	-0.19	ns
Alpha	CEN	Control	Pre vs Post	32	-0.0169	0.0036	118.0	-4.75	< .001	-1.19	***
Alpha	CEN	Control	Pre vs Post4W	32	-0.0134	0.0036	118.0	-3.76	.001	-0.94	**
Alpha	CEN	Control	Post vs Post4W	32	+0.0035	0.0036	118.0	0.99	.529	+0.25	ns
Beta	DMN	Chiro	Pre vs Post	29	+0.0690	0.0159	112.0	4.35	< .001	+1.14	***
Beta	DMN	Chiro	Pre vs Post4W	29	+0.0796	0.0159	112.0	5.02	< .001	+1.32	***
Beta	DMN	Chiro	Post vs Post4W	29	+0.0106	0.0159	112.0	0.67	.667	+0.17	ns
Beta	DMN	Control	Pre vs Post	29	+0.0024	0.0159	112.0	0.15	.895	+0.04	ns
Beta	DMN	Control	Pre vs Post4W	29	+0.0252	0.0159	112.0	1.59	.276	+0.42	ns

Band	Network	Group	Contrast	N	z estimate	SE	df	t	q	d_{LMM}	Sig
Beta	DMN	Control	Post vs Post4W	29	+0.0228	0.0159	112.0	1.44	.344	+0.38	ns
Beta	SN	Chiro	Pre vs Post	29	-0.0047	0.0230	112.0	-0.20	.875	-0.05	ns
Beta	SN	Chiro	Pre vs Post4W	29	+0.0243	0.0230	112.0	1.06	.521	+0.28	ns
Beta	SN	Chiro	Post vs Post4W	29	+0.0290	0.0230	112.0	1.26	.421	+0.33	ns
Beta	SN	Control	Pre vs Post	29	+0.0237	0.0230	112.0	1.03	.521	+0.27	ns
Beta	SN	Control	Pre vs Post4W	29	+0.0365	0.0230	112.0	1.59	.276	+0.42	ns
Beta	SN	Control	Post vs Post4W	29	+0.0128	0.0230	112.0	0.56	.707	+0.15	ns
Beta	CEN	Chiro	Pre vs Post	29	-0.0481	0.0191	112.0	-2.51	.046	-0.66	*
Beta	CEN	Chiro	Pre vs Post4W	29	-0.0046	0.0191	112.0	-0.24	.866	-0.06	ns
Beta	CEN	Chiro	Post vs Post4W	29	+0.0435	0.0191	112.0	2.27	.082	+0.60	ns
Beta	CEN	Control	Pre vs Post	29	+0.0080	0.0191	112.0	0.42	.784	+0.11	ns
Beta	CEN	Control	Pre vs Post4W	29	+0.0155	0.0191	112.0	0.81	.620	+0.21	ns
Beta	CEN	Control	Post vs Post4W	29	+0.0074	0.0191	112.0	0.39	.786	+0.10	ns
Gamma	DMN	Chiro	Pre vs Post	29	-0.1640	0.0261	112.0	-6.28	$< .001$	-1.65	***
Gamma	DMN	Chiro	Pre vs Post4W	29	-0.1442	0.0261	112.0	-5.52	$< .001$	-1.45	***
Gamma	DMN	Chiro	Post vs Post4W	29	+0.0199	0.0261	112.0	0.76	.645	+0.20	ns
Gamma	DMN	Control	Pre vs Post	29	-0.0537	0.0261	112.0	-2.06	.126	-0.54	ns
Gamma	DMN	Control	Pre vs Post4W	29	-0.0263	0.0261	112.0	-1.01	.528	-0.26	ns
Gamma	DMN	Control	Post vs Post4W	29	+0.0274	0.0261	112.0	1.05	.521	+0.28	ns
Gamma	SN	Chiro	Pre vs Post	29	+0.0259	0.0356	112.0	0.73	.660	+0.19	ns
Gamma	SN	Chiro	Pre vs Post4W	29	-0.0028	0.0356	112.0	-0.08	.938	-0.02	ns
Gamma	SN	Chiro	Post vs Post4W	29	-0.0287	0.0356	112.0	-0.81	.620	-0.21	ns
Gamma	SN	Control	Pre vs Post	29	-0.0449	0.0356	112.0	-1.26	.421	-0.33	ns
Gamma	SN	Control	Pre vs Post4W	29	-0.0238	0.0356	112.0	-0.67	.667	-0.18	ns
Gamma	SN	Control	Post vs Post4W	29	+0.0211	0.0356	112.0	0.59	.707	+0.16	ns
Gamma	CEN	Chiro	Pre vs Post	29	-0.0350	0.0248	112.0	-1.41	.349	-0.37	ns
Gamma	CEN	Chiro	Pre vs Post4W	29	-0.0211	0.0248	112.0	-0.85	.620	-0.22	ns
Gamma	CEN	Chiro	Post vs Post4W	29	+0.0139	0.0248	112.0	0.56	.707	+0.15	ns
Gamma	CEN	Control	Pre vs Post	29	+0.0164	0.0248	112.0	0.66	.667	+0.17	ns
Gamma	CEN	Control	Pre vs Post4W	29	+0.0446	0.0248	112.0	1.80	.191	+0.47	ns
Gamma	CEN	Control	Post vs Post4W	29	+0.0282	0.0248	112.0	1.14	.473	+0.30	ns

S4. Somatosensory evoked potentials: all model-estimated within-group contrasts

Table S4: SEP EEG: all model-estimated within-group session contrasts, including non-significant comparisons. Estimates and SEs are on the Fisher-z scale. Fractional df values are Satterthwaite denominator degrees of freedom based on the repeated participant-session responses and fitted variance components in the corresponding LMM; they are not counts of independent participants.

Band	Network	Group	Contrast	N	z estimate	SE	df	t	q	d_{LMM}	Sig
Theta	DMN	Chiropractic	Post–Pre	29	+0.1296	0.0827	177.0	1.57	.505	+0.41	ns
Theta	DMN	Chiropractic	Post4W–Pre	29	+0.0054	0.0827	177.0	0.06	.984	+0.02	ns
Theta	DMN	Chiropractic	Post4W–Post	29	-0.1242	0.0827	177.0	-1.50	.512	-0.39	ns
Theta	DMN	Control	Post–Pre	32	-0.1444	0.0788	177.0	-1.83	.413	-0.46	ns
Theta	DMN	Control	Post4W–Pre	32	-0.1024	0.0788	177.0	-1.30	.585	-0.33	ns
Theta	DMN	Control	Post4W–Post	32	+0.0419	0.0788	177.0	0.53	.877	+0.13	ns
Theta	SN	Chiropractic	Post–Pre	29	+0.1731	0.1241	118.0	1.40	.558	+0.37	ns
Theta	SN	Chiropractic	Post4W–Pre	29	+0.0154	0.1241	118.0	0.12	.979	+0.03	ns
Theta	SN	Chiropractic	Post4W–Post	29	-0.1577	0.1241	118.0	-1.27	.594	-0.33	ns
Theta	SN	Control	Post–Pre	32	-0.2185	0.1181	118.0	-1.85	.413	-0.46	ns
Theta	SN	Control	Post4W–Pre	32	-0.0079	0.1181	118.0	-0.07	.984	-0.02	ns
Theta	SN	Control	Post4W–Post	32	+0.2107	0.1181	118.0	1.78	.427	+0.45	ns
Theta	CEN	Chiropractic	Post–Pre	29	+0.0604	0.0889	177.0	0.68	.810	+0.18	ns
Theta	CEN	Chiropractic	Post4W–Pre	29	+0.0011	0.0889	177.0	0.01	.990	+0.00	ns

Band	Network	Group	Contrast	N	z estimate	SE	df	t	q	d_{LMM}	Sig
Theta	CEN	Chiropractic	Post4W–Post	29	-0.0593	0.0889	177.0	-0.67	.810	-0.18	ns
Theta	CEN	Control	Post–Pre	32	-0.1690	0.0847	177.0	-2.00	.413	-0.50	ns
Theta	CEN	Control	Post4W–Pre	32	-0.1443	0.0847	177.0	-1.70	.432	-0.43	ns
Theta	CEN	Control	Post4W–Post	32	+0.0247	0.0847	177.0	0.29	.914	+0.07	ns
Alpha	DMN	Chiropractic	Post–Pre	29	+0.0758	0.0342	177.0	2.22	.413	+0.58	ns
Alpha	DMN	Chiropractic	Post4W–Pre	29	+0.0628	0.0342	177.0	1.84	.413	+0.48	ns
Alpha	DMN	Chiropractic	Post4W–Post	29	-0.0130	0.0342	177.0	-0.38	.914	-0.10	ns
Alpha	DMN	Control	Post–Pre	32	-0.0499	0.0325	177.0	-1.53	.509	-0.38	ns
Alpha	DMN	Control	Post4W–Pre	32	+0.0109	0.0325	177.0	0.34	.914	+0.08	ns
Alpha	DMN	Control	Post4W–Post	32	+0.0608	0.0325	177.0	1.87	.413	+0.47	ns
Alpha	SN	Chiropractic	Post–Pre	29	+0.0414	0.0546	177.0	0.76	.810	+0.20	ns
Alpha	SN	Chiropractic	Post4W–Pre	29	-0.0158	0.0546	177.0	-0.29	.914	-0.08	ns
Alpha	SN	Chiropractic	Post4W–Post	29	-0.0572	0.0546	177.0	-1.05	.683	-0.28	ns
Alpha	SN	Control	Post–Pre	32	+0.0134	0.0520	177.0	0.26	.914	+0.06	ns
Alpha	SN	Control	Post4W–Pre	32	+0.0011	0.0520	177.0	0.02	.990	+0.01	ns
Alpha	SN	Control	Post4W–Post	32	-0.0123	0.0520	177.0	-0.24	.914	-0.06	ns
Alpha	CEN	Chiropractic	Post–Pre	29	+0.0969	0.0388	177.0	2.50	.413	+0.66	ns
Alpha	CEN	Chiropractic	Post4W–Pre	29	+0.0436	0.0388	177.0	1.12	.674	+0.30	ns
Alpha	CEN	Chiropractic	Post4W–Post	29	-0.0533	0.0388	177.0	-1.38	.558	-0.36	ns
Alpha	CEN	Control	Post–Pre	32	-0.0700	0.0369	177.0	-1.90	.413	-0.47	ns
Alpha	CEN	Control	Post4W–Pre	32	-0.0360	0.0369	177.0	-0.98	.700	-0.24	ns
Alpha	CEN	Control	Post4W–Post	32	+0.0340	0.0369	177.0	0.92	.715	+0.23	ns
Beta	DMN	Chiropractic	Post–Pre	29	+0.0096	0.0404	118.0	0.24	.914	+0.06	ns
Beta	DMN	Chiropractic	Post4W–Pre	29	+0.0409	0.0404	118.0	1.01	.683	+0.27	ns
Beta	DMN	Chiropractic	Post4W–Post	29	+0.0313	0.0404	118.0	0.77	.810	+0.20	ns
Beta	DMN	Control	Post–Pre	32	+0.0263	0.0385	118.0	0.68	.810	+0.17	ns
Beta	DMN	Control	Post4W–Pre	32	+0.0068	0.0385	118.0	0.18	.952	+0.04	ns
Beta	DMN	Control	Post4W–Post	32	-0.0194	0.0385	118.0	-0.51	.879	-0.13	ns
Beta	SN	Chiropractic	Post–Pre	29	-0.0350	0.0885	118.0	-0.40	.914	-0.10	ns
Beta	SN	Chiropractic	Post4W–Pre	29	+0.0905	0.0885	118.0	1.02	.683	+0.27	ns
Beta	SN	Chiropractic	Post4W–Post	29	+0.1255	0.0885	118.0	1.42	.558	+0.37	ns
Beta	SN	Control	Post–Pre	32	+0.0473	0.0843	118.0	0.56	.877	+0.14	ns
Beta	SN	Control	Post4W–Pre	32	-0.0235	0.0843	118.0	-0.28	.914	-0.07	ns
Beta	SN	Control	Post4W–Post	32	-0.0708	0.0843	118.0	-0.84	.783	-0.21	ns
Beta	CEN	Chiropractic	Post–Pre	29	-0.0049	0.0437	118.0	-0.11	.979	-0.03	ns
Beta	CEN	Chiropractic	Post4W–Pre	29	+0.0450	0.0437	118.0	1.03	.683	+0.27	ns
Beta	CEN	Chiropractic	Post4W–Post	29	+0.0499	0.0437	118.0	1.14	.674	+0.30	ns
Beta	CEN	Control	Post–Pre	32	+0.0281	0.0416	118.0	0.67	.810	+0.17	ns
Beta	CEN	Control	Post4W–Pre	32	-0.0484	0.0416	118.0	-1.16	.674	-0.29	ns
Beta	CEN	Control	Post4W–Post	32	-0.0764	0.0416	118.0	-1.84	.413	-0.46	ns
Gamma	DMN	Chiropractic	Post–Pre	29	-0.0382	0.0535	118.0	-0.71	.810	-0.19	ns
Gamma	DMN	Chiropractic	Post4W–Pre	29	-0.0164	0.0535	118.0	-0.31	.914	-0.08	ns
Gamma	DMN	Chiropractic	Post4W–Post	29	+0.0218	0.0535	118.0	0.41	.914	+0.11	ns
Gamma	DMN	Control	Post–Pre	32	+0.1066	0.0509	118.0	2.09	.413	+0.52	ns
Gamma	DMN	Control	Post4W–Pre	32	+0.0817	0.0509	118.0	1.60	.501	+0.40	ns
Gamma	DMN	Control	Post4W–Post	32	-0.0249	0.0509	118.0	-0.49	.879	-0.12	ns
Gamma	SN	Chiropractic	Post–Pre	29	-0.0902	0.0883	118.0	-1.02	.683	-0.27	ns
Gamma	SN	Chiropractic	Post4W–Pre	29	-0.1738	0.0883	118.0	-1.97	.413	-0.52	ns
Gamma	SN	Chiropractic	Post4W–Post	29	-0.0836	0.0883	118.0	-0.95	.712	-0.25	ns
Gamma	SN	Control	Post–Pre	32	+0.0492	0.0841	118.0	0.59	.875	+0.15	ns
Gamma	SN	Control	Post4W–Pre	32	-0.0615	0.0841	118.0	-0.73	.810	-0.18	ns
Gamma	SN	Control	Post4W–Post	32	-0.1107	0.0841	118.0	-1.32	.585	-0.33	ns
Gamma	CEN	Chiropractic	Post–Pre	29	-0.0922	0.0539	118.0	-1.71	.432	-0.45	ns
Gamma	CEN	Chiropractic	Post4W–Pre	29	-0.1091	0.0539	118.0	-2.03	.413	-0.53	ns
Gamma	CEN	Chiropractic	Post4W–Post	29	-0.0169	0.0539	118.0	-0.31	.914	-0.08	ns
Gamma	CEN	Control	Post–Pre	32	+0.0272	0.0513	118.0	0.53	.877	+0.13	ns
Gamma	CEN	Control	Post4W–Pre	32	+0.0028	0.0513	118.0	0.05	.984	+0.01	ns
Gamma	CEN	Control	Post4W–Post	32	-0.0244	0.0513	118.0	-0.48	.879	-0.12	ns

S5. Baseline resting-state network-mean PLI

Table S5: Baseline raw resting-state network-mean PLI by group.

Band	Network	Group	N	Mean PLI	SD
Theta	DMN	Chiro	29	0.3553	0.0099
Theta	DMN	Control	29	0.3578	0.0137
Theta	SN	Chiro	29	0.3598	0.0151
Theta	SN	Control	29	0.3587	0.0149
Theta	CEN	Chiro	29	0.3553	0.0101
Theta	CEN	Control	29	0.3557	0.0132
Alpha	DMN	Chiro	29	0.3844	0.0131
Alpha	DMN	Control	32	0.3836	0.0113
Alpha	SN	Chiro	29	0.3870	0.0173
Alpha	SN	Control	32	0.3817	0.0164
Alpha	CEN	Chiro	29	0.3855	0.0129
Alpha	CEN	Control	32	0.3819	0.0137
Beta	DMN	Chiro	29	0.5892	0.0450
Beta	DMN	Control	29	0.5856	0.0432
Beta	SN	Chiro	29	0.6007	0.0558
Beta	SN	Control	29	0.5918	0.0586
Beta	CEN	Chiro	29	0.5916	0.0498
Beta	CEN	Control	29	0.5882	0.0521
Gamma	DMN	Chiro	29	0.8818	0.0183
Gamma	DMN	Control	29	0.8728	0.0210
Gamma	SN	Chiro	29	0.8846	0.0288
Gamma	SN	Control	29	0.8764	0.0305
Gamma	CEN	Chiro	29	0.8791	0.0207
Gamma	CEN	Control	29	0.8859	0.0214

S6. Baseline SEP network-mean PLI

Table S6: Baseline raw SEP network-mean PLI by group.

Band	Network	Group	N	Mean PLI	SD
Theta	DMN	Chiropractic	29	0.6843	0.1615
Theta	DMN	Control	32	0.7216	0.1038
Theta	SN	Chiropractic	29	0.6568	0.2299
Theta	SN	Control	32	0.7364	0.1545
Theta	CEN	Chiropractic	29	0.6873	0.1590
Theta	CEN	Control	32	0.7281	0.1239
Alpha	DMN	Chiropractic	29	0.6233	0.0954
Alpha	DMN	Control	32	0.6600	0.0773
Alpha	SN	Chiropractic	29	0.6501	0.1238
Alpha	SN	Control	32	0.6570	0.1176
Alpha	CEN	Chiropractic	29	0.6267	0.0936
Alpha	CEN	Control	32	0.6778	0.0887
Beta	DMN	Chiropractic	29	0.3746	0.1603
Beta	DMN	Control	32	0.3701	0.1519
Beta	SN	Chiropractic	29	0.4138	0.2423

Band	Network	Group	N	Mean PLI	SD
Beta	SN	Control	32	0.3829	0.2136
Beta	CEN	Chiropractic	29	0.3938	0.1639
Beta	CEN	Control	32	0.3928	0.1861
Gamma	DMN	Chiropractic	29	0.6119	0.1274
Gamma	DMN	Control	32	0.5773	0.1537
Gamma	SN	Chiropractic	29	0.6286	0.2039
Gamma	SN	Control	32	0.5885	0.2199
Gamma	CEN	Chiropractic	29	0.6572	0.1381
Gamma	CEN	Control	32	0.6098	0.1475

S7. Omnibus Group-by-Session tests

Table S7: Type III Group-by-Session tests from every Fisher-z LMM. df1 is the numerator degree of freedom and df2 is the Satterthwaite denominator degree of freedom based on the repeated participant-session responses and fitted variance components; df2 is not a count of independent participants. q-values are FDR-corrected across the 12 interactions within each recording condition.

Condition	Band	Network	df1	df2	F	q	Sig
Resting-state	Theta	DMN	2	112.0	14.63	$< .001$	***
Resting-state	Theta	SN	2	112.0	8.60	$< .001$	***
Resting-state	Theta	CEN	2	112.0	55.15	$< .001$	***
Resting-state	Alpha	DMN	2	118.0	311.16	$< .001$	***
Resting-state	Alpha	SN	2	118.0	15.09	$< .001$	***
Resting-state	Alpha	CEN	2	118.0	20.03	$< .001$	***
Resting-state	Beta	DMN	2	112.0	5.00	.012	*
Resting-state	Beta	SN	2	112.0	0.38	.682	ns
Resting-state	Beta	CEN	2	112.0	2.21	.153	ns
Resting-state	Gamma	DMN	2	112.0	6.38	.004	**
Resting-state	Gamma	SN	2	112.0	1.05	.387	ns
Resting-state	Gamma	CEN	2	112.0	1.95	.177	ns
SEP	Theta	DMN	2	177.0	2.92	.170	ns
SEP	Theta	SN	2	118.0	3.29	.163	ns
SEP	Theta	CEN	2	177.0	1.79	.292	ns
SEP	Alpha	DMN	2	177.0	3.58	.163	ns
SEP	Alpha	SN	2	177.0	0.18	.835	ns
SEP	Alpha	CEN	2	177.0	4.87	.105	ns
SEP	Beta	DMN	2	118.0	0.43	.711	ns
SEP	Beta	SN	2	118.0	1.30	.368	ns
SEP	Beta	CEN	2	118.0	2.36	.238	ns
SEP	Gamma	DMN	2	118.0	2.00	.280	ns
SEP	Gamma	SN	2	118.0	0.74	.578	ns
SEP	Gamma	CEN	2	118.0	1.62	.304	ns

S8. Model validation and sensitivity analyses

Data provenance and model structure

- Resting-state: source workbook `PLI_Table-UP3_TEST.xlsm`, 2,115 rows.

- SEP: every SEP connectivity matrix was derived from the condition-specific stimulus-locked sensor recordings, with no resting-state input at any stage. All SEP tables (S2, S4, S6, and the SEP rows of S7) and SEP figures report these condition-specific values.

The SEP baseline values (Table S6) differ substantially from the resting-state baseline values (Table S5) in every band, network, and group, and the participant-level comparison in Section S10 confirms the two conditions are numerically distinct. Participant numbers 1–29 occurred in both groups, so the clustering variable was defined as the interaction of group and participant number. Participant was the independent experimental unit for the group comparison; participant-session was the repeated observational unit within a network-by-band model. Satterthwaite degrees of freedom therefore reflect the information in the repeated participant-session responses after accounting for participant clustering and are not a count of independent participants.

Table S8: Convergence, optimizer, and outlier-diagnostic summary. Raw MAD flags used a threshold of 3.5 scaled median absolute deviations within each Group by Session by Frequency by Network cell. Residual flags used an absolute standardized residual above 3. For the SEP condition, five models returned a boundary (singular) fit with the participant variance estimated at zero; fixed effects agreed across all successful optimizer configurations. Residual-versus-fitted and Q–Q diagnostics are shown in Figures S6 and S7.

Condition	Models	Singular	allFit success	allFit singular	MAD flags	Residual flags
Resting-state	12	0	84/84	0	27	13
SEP	12	5	79/84	30	n/a	n/a

The five boundary-singular SEP models were theta DMN, theta CEN, alpha DMN, alpha SN, and alpha CEN. In each the estimated random-intercept standard deviation and the corresponding intraclass correlation were zero, so the Satterthwaite denominator degrees of freedom for those models equal the residual degrees of freedom. The five unsuccessful allFit runs were the `nmkbw` optimizer applied to those same five models.

Bonferroni sensitivity

In the resting-state condition (retained), seven of eight FDR-significant omnibus interactions survived Bonferroni correction (the resting beta-DMN interaction, $q = .012$, did not); 14 of 17 FDR-significant between-group contrasts and 19 of 21 within-group contrasts survived. Resting-state contrasts that did not meet the Bonferroni threshold are retained in the complete tables but interpreted cautiously. All fixed-effect estimates agreed across the successful optimizer configurations to within 4×10^{-15} in the resting-state condition and 8×10^{-16} in the SEP condition. No observations were deleted or modified for the primary analysis. The MAD-winsorized analysis was conducted only as a sensitivity analysis by capping flagged values at the corresponding cell median plus or minus 3.5 scaled MADs.

Table S9: Alpha-DMN contrast t-statistics before and after the MAD-winsorized sensitivity analysis. Positive percentage changes indicate a larger absolute t-statistic after winsorization. Only the retained resting-state alpha-DMN contrasts are shown.

Condition	Session	Raw-data t	Winsorized t	Change in $\|t\|$
Resting-state	Post	31.64	33.28	+5.2%
Resting-state	Post4W	26.15	27.63	+5.7%

Table S10: Leave-one-participant-out sensitivity for alpha-DMN. Each LMM was refitted after omitting one unique participant. Only the retained resting-state alpha-DMN contrasts are shown.

Condition	Session	Full t	LOO $\|t\|$ range	Maximum change	Any singular
Resting-state	Post	31.64	31.05–32.39	2.4%	No
Resting-state	Post4W	26.15	25.65–27.14	3.8%	No

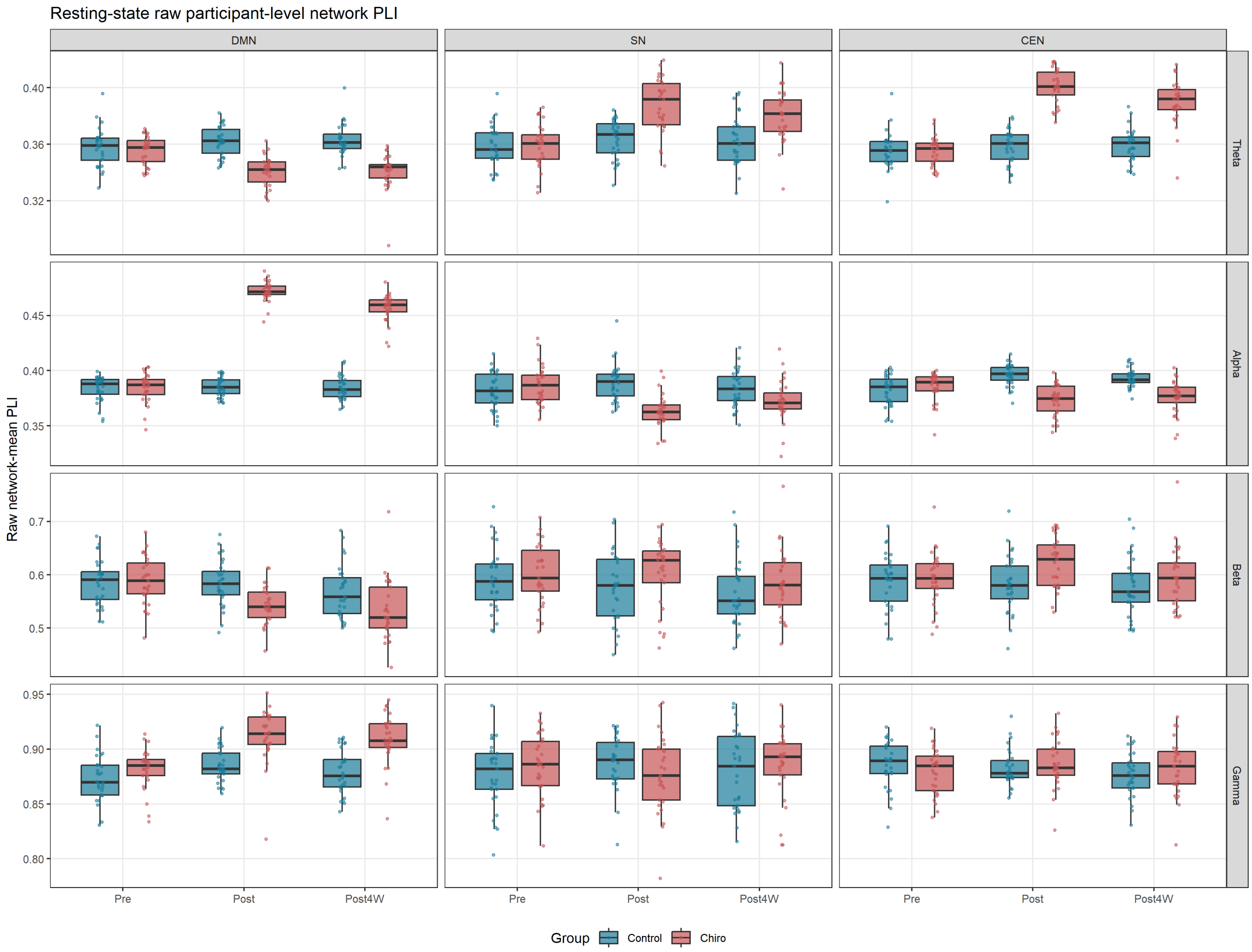

Figure S1: Raw participant-level resting-state network-mean PLI. Every point is one participant-session network mean; boxplots are descriptive and were not the inferential model.

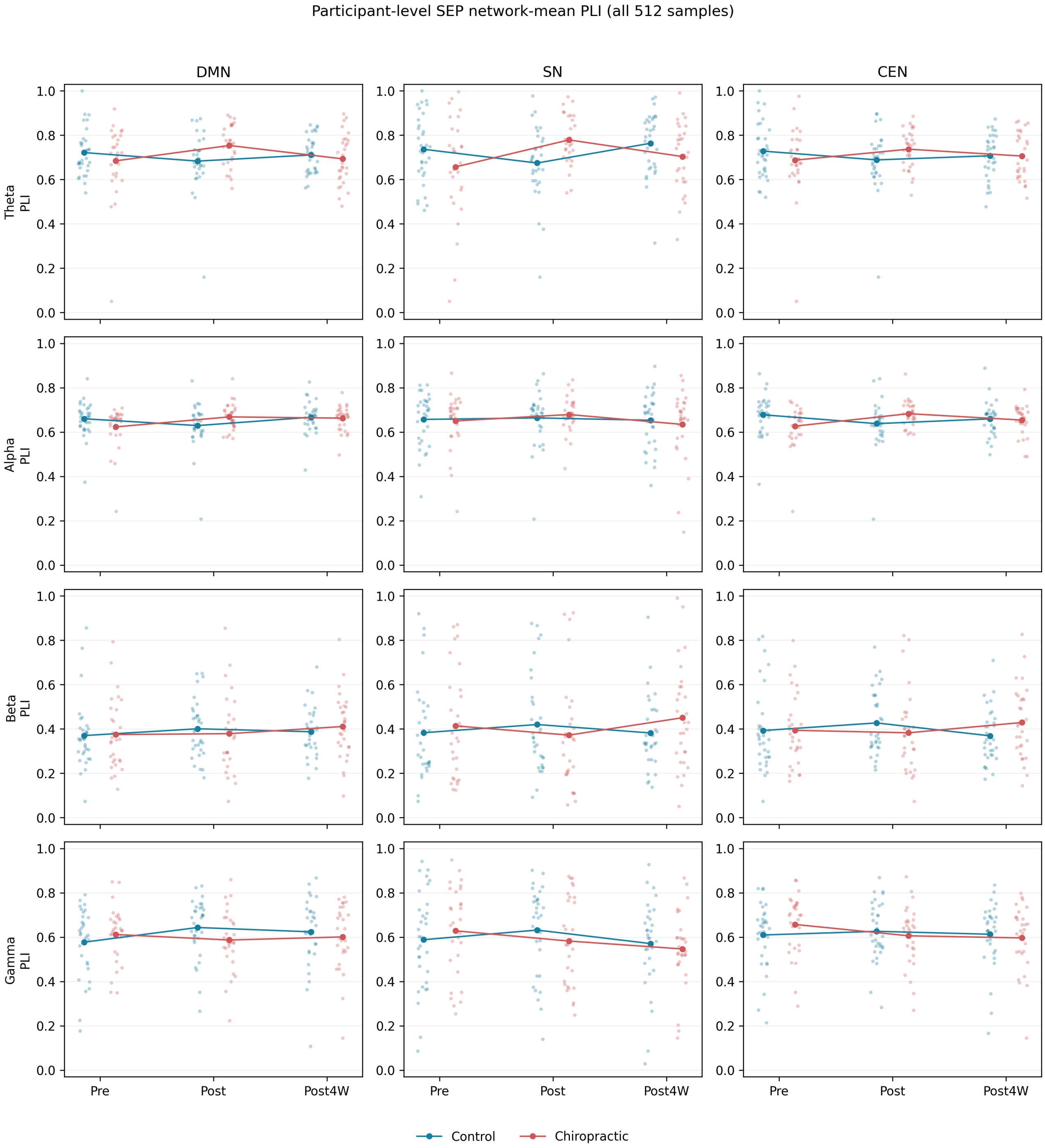

Figure S2: Raw participant-level SEP network-mean PLI. Every point is one participant-session network mean; boxplots are descriptive and were not the inferential model.

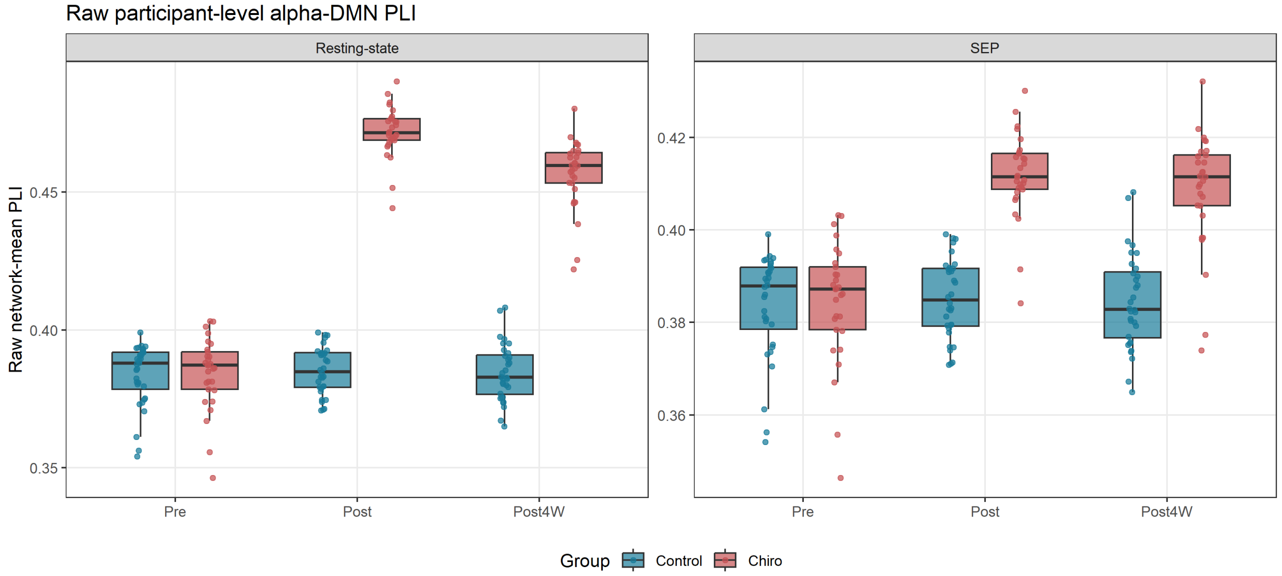

Figure S3: Raw participant-level alpha-DMN PLI for both recording conditions. The post-treatment group separation in the resting-state condition is distributed across participants rather than created by one isolated observation. The condition-specific SEP raw distributions are shown in Figure S2 and Table S6.

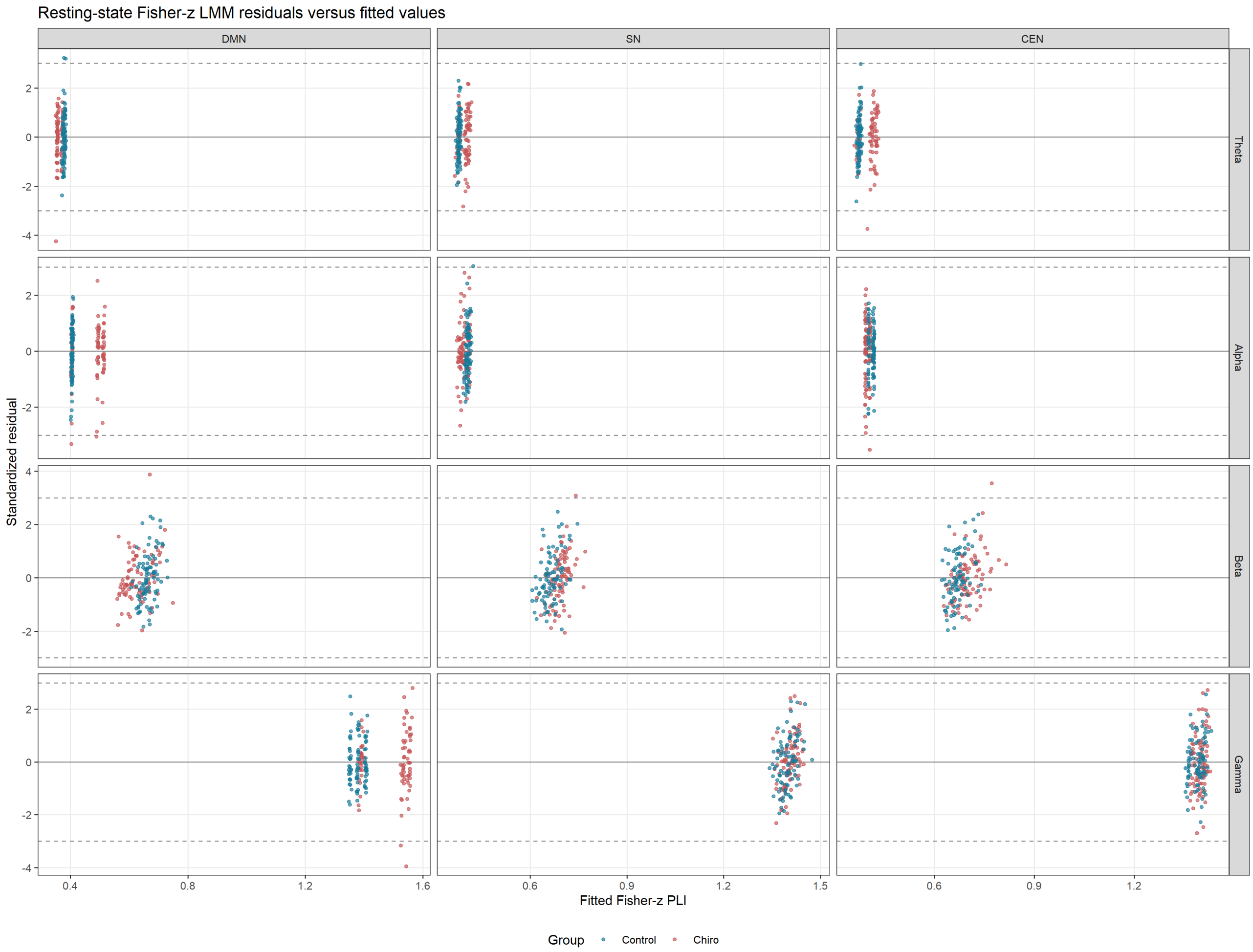

Figure S4: Resting-state Fisher-z LMM standardized residuals versus fitted values. Dashed lines mark standardized residuals of ± 3.

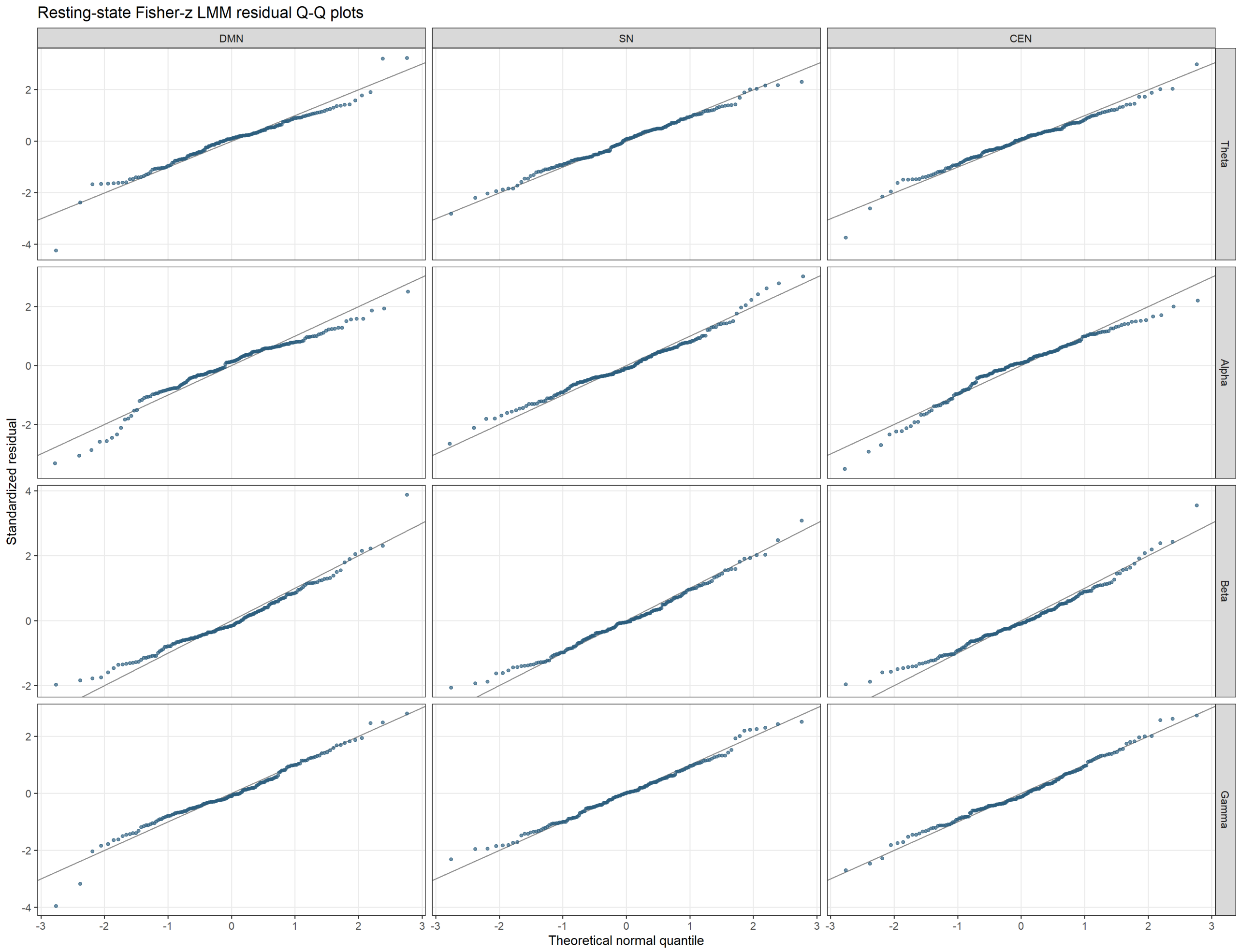

Figure S5: Resting-state Fisher-z LMM residual Q-Q plots.

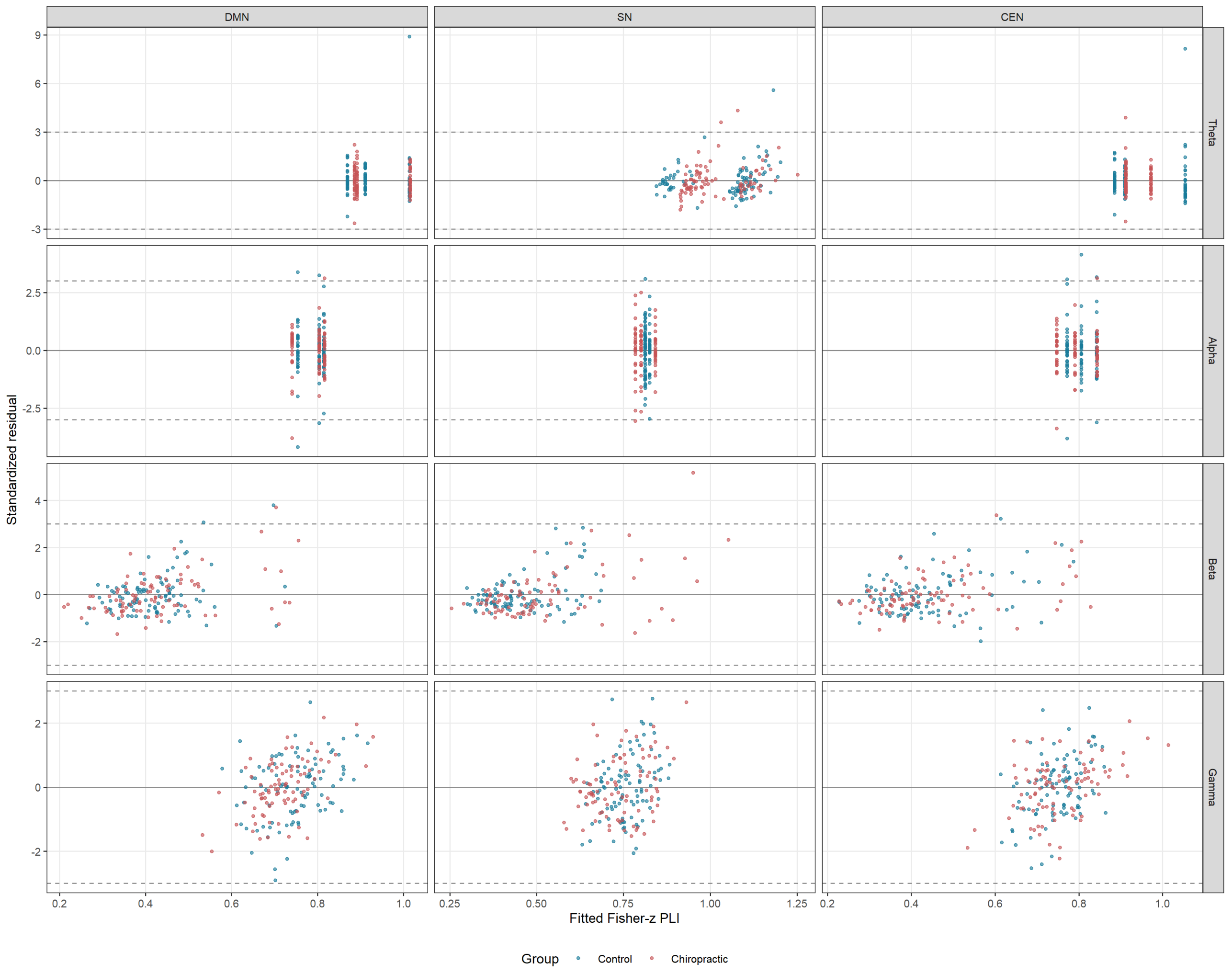

Figure S6: SEP Fisher-z LMM standardized residuals versus fitted values. Dashed lines mark standardized residuals of ±3.

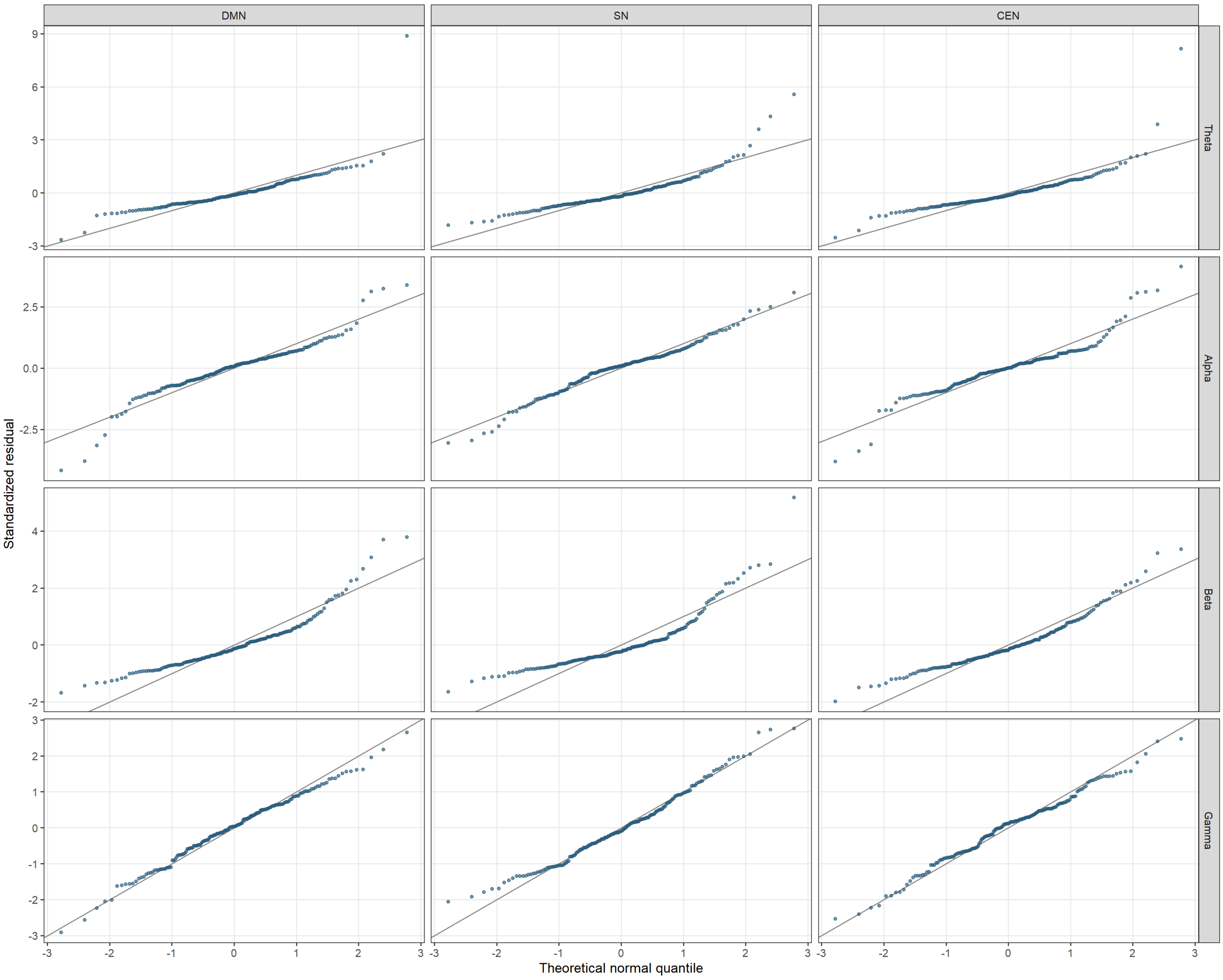

Figure S7: SEP Fisher-z LMM residual Q-Q plots.

S9. Sample-size sensitivity benchmark

Because this exploratory secondary analysis used all eligible connectivity matrices from the parent trial, no prospective network-level power calculation determined the sample size. As an approximate independent-groups sensitivity benchmark, 29 chiropractic and 29 to 32 control participants would provide 80% power to detect a standardized mean difference of approximately 0.73 to 0.75 and 90% power to detect approximately 0.85 to 0.87, using a two-sided test at $\alpha = .05$. This benchmark is not a power calculation for the repeated-measures LMM; the primary inference comes from the Fisher-z LMMs and their model-estimated contrasts.

S10. Participant-level comparison of resting-state and SEP connectivity

To confirm the two recording conditions are numerically distinct, resting-state and SEP network-mean PLI values were paired at the participant × session × band × network × group level. Of 2,115 matched values, **none was exactly equal** across conditions (81 further SEP-only cells had no resting-state counterpart), and cell-level Pearson correlations ranged from −.382 **to** .443. Table S11 gives, per cell, the resting-state and SEP means, their difference (SEP minus resting), the within-cell correlation, and the number of exact matches; Figures S8 and S9 show the scatter and Bland–Altman comparison.

Table S11: Participant-level comparison of resting-state and SEP network-mean PLI. "Corr" is the within-cell Pearson correlation between paired resting-state and SEP values; "Exact" is the number of exactly equal paired values.

**Band**	**Net**	**Group**	**Session**	**Rest**	**SEP**	**Diff**	**Corr**	**Exact**
Theta	DMN	Chiropractic	Pre	0.3553	0.6843	+0.3289	-0.04	0
Theta	DMN	Chiropractic	Post	0.3407	0.7535	+0.4128	0.19	0
Theta	DMN	Chiropractic	Post4W	0.3411	0.6931	+0.3520	0.15	0
Theta	DMN	Control	Pre	0.3578	0.7135	+0.3557	0.07	0
Theta	DMN	Control	Post	0.3619	0.6702	+0.3083	-0.02	0
Theta	DMN	Control	Post4W	0.3632	0.6984	+0.3352	0.31	0
Theta	SN	Chiropractic	Pre	0.3598	0.6568	+0.2970	-0.06	0
Theta	SN	Chiropractic	Post	0.3877	0.7786	+0.3909	0.15	0
Theta	SN	Chiropractic	Post4W	0.3802	0.7036	+0.3234	0.12	0
Theta	SN	Control	Pre	0.3587	0.7338	+0.3751	0.08	0
Theta	SN	Control	Post	0.3637	0.6624	+0.2988	0.19	0
Theta	SN	Control	Post4W	0.3623	0.7491	+0.3869	0.16	0
Theta	CEN	Chiropractic	Pre	0.3553	0.6873	+0.3320	-0.23	0
Theta	CEN	Chiropractic	Post	0.4009	0.7369	+0.3360	-0.02	0
Theta	CEN	Chiropractic	Post4W	0.3896	0.7059	+0.3164	0.35	0
Theta	CEN	Control	Pre	0.3557	0.7168	+0.3611	0.19	0
Theta	CEN	Control	Post	0.3582	0.6826	+0.3244	-0.09	0
Theta	CEN	Control	Post4W	0.3594	0.6930	+0.3336	0.20	0
Alpha	DMN	Chiropractic	Pre	0.3844	0.6233	+0.2389	0.11	0
Alpha	DMN	Chiropractic	Post	0.4717	0.6684	+0.1967	-0.16	0
Alpha	DMN	Chiropractic	Post4W	0.4567	0.6628	+0.2061	0.04	0
Alpha	DMN	Control	Pre	0.3836	0.6600	+0.2764	0.07	0
Alpha	DMN	Control	Post	0.3852	0.6292	+0.2440	0.35	0
Alpha	DMN	Control	Post4W	0.3845	0.6669	+0.2823	0.06	0
Alpha	SN	Chiropractic	Pre	0.3870	0.6501	+0.2631	0.06	0
Alpha	SN	Chiropractic	Post	0.3633	0.6790	+0.3157	-0.01	0
Alpha	SN	Chiropractic	Post4W	0.3724	0.6345	+0.2621	-0.21	0
Alpha	SN	Control	Pre	0.3817	0.6570	+0.2753	0.14	0
Alpha	SN	Control	Post	0.3892	0.6639	+0.2747	0.34	0
Alpha	SN	Control	Post4W	0.3833	0.6533	+0.2699	0.21	0
Alpha	CEN	Chiropractic	Pre	0.3855	0.6267	+0.2413	-0.11	0
Alpha	CEN	Chiropractic	Post	0.3735	0.6830	+0.3095	0.03	0
Alpha	CEN	Chiropractic	Post4W	0.3758	0.6541	+0.2783	-0.38	0
Alpha	CEN	Control	Pre	0.3819	0.6778	+0.2959	0.27	0
Alpha	CEN	Control	Post	0.3963	0.6380	+0.2417	0.36	0
Alpha	CEN	Control	Post4W	0.3934	0.6595	+0.2661	-0.20	0
Beta	DMN	Chiropractic	Pre	0.5892	0.3746	-0.2146	-0.29	0
Beta	DMN	Chiropractic	Post	0.5433	0.3787	-0.1646	-0.25	0
Beta	DMN	Chiropractic	Post4W	0.5341	0.4109	-0.1232	-0.18	0
Beta	DMN	Control	Pre	0.5856	0.3722	-0.2134	-0.06	0
Beta	DMN	Control	Post	0.5841	0.4147	-0.1694	-0.04	0
Beta	DMN	Control	Post4W	0.5684	0.3905	-0.1779	0.04	0
Beta	SN	Chiropractic	Pre	0.6007	0.4138	-0.1868	-0.09	0
Beta	SN	Chiropractic	Post	0.6030	0.3721	-0.2309	-0.35	0

Band	Net	Group	Session	Rest	SEP	Diff	Corr	Exact
Beta	SN	Chiropractic	Post4W	0.5842	0.4505	-0.1337	0.02	0
Beta	SN	Control	Pre	0.5918	0.3843	-0.2076	-0.23	0
Beta	SN	Control	Post	0.5759	0.4385	-0.1374	-0.19	0
Beta	SN	Control	Post4W	0.5675	0.3803	-0.1871	-0.05	0
Beta	CEN	Chiropractic	Pre	0.5916	0.3938	-0.1978	-0.24	0
Beta	CEN	Chiropractic	Post	0.6219	0.3825	-0.2394	-0.38	0
Beta	CEN	Chiropractic	Post4W	0.5936	0.4290	-0.1646	-0.14	0
Beta	CEN	Control	Pre	0.5882	0.3873	-0.2009	-0.19	0
Beta	CEN	Control	Post	0.5827	0.4388	-0.1439	-0.26	0
Beta	CEN	Control	Post4W	0.5776	0.3657	-0.2120	-0.33	0
Gamma	DMN	Chiropractic	Pre	0.8818	0.6119	-0.2699	0.22	0
Gamma	DMN	Chiropractic	Post	0.9118	0.5871	-0.3247	0.08	0
Gamma	DMN	Chiropractic	Post4W	0.9086	0.6010	-0.3077	-0.17	0
Gamma	DMN	Control	Pre	0.8728	0.5697	-0.3032	0.18	0
Gamma	DMN	Control	Post	0.8856	0.6578	-0.2278	0.44	0
Gamma	DMN	Control	Post4W	0.8790	0.6441	-0.2349	0.23	0
Gamma	SN	Chiropractic	Pre	0.8846	0.6286	-0.2560	-0.16	0
Gamma	SN	Chiropractic	Post	0.8776	0.5823	-0.2952	-0.13	0
Gamma	SN	Chiropractic	Post4W	0.8849	0.5467	-0.3382	-0.03	0
Gamma	SN	Control	Pre	0.8764	0.5929	-0.2835	0.06	0
Gamma	SN	Control	Post	0.8874	0.6533	-0.2340	-0.08	0
Gamma	SN	Control	Post4W	0.8800	0.5832	-0.2968	0.11	0
Gamma	CEN	Chiropractic	Pre	0.8791	0.6572	-0.2219	0.27	0
Gamma	CEN	Chiropractic	Post	0.8864	0.6061	-0.2804	0.03	0
Gamma	CEN	Chiropractic	Post4W	0.8830	0.5964	-0.2866	-0.14	0
Gamma	CEN	Control	Pre	0.8859	0.6076	-0.2783	0.32	0
Gamma	CEN	Control	Post	0.8828	0.6432	-0.2396	0.20	0
Gamma	CEN	Control	Post4W	0.8763	0.6280	-0.2483	0.16	0

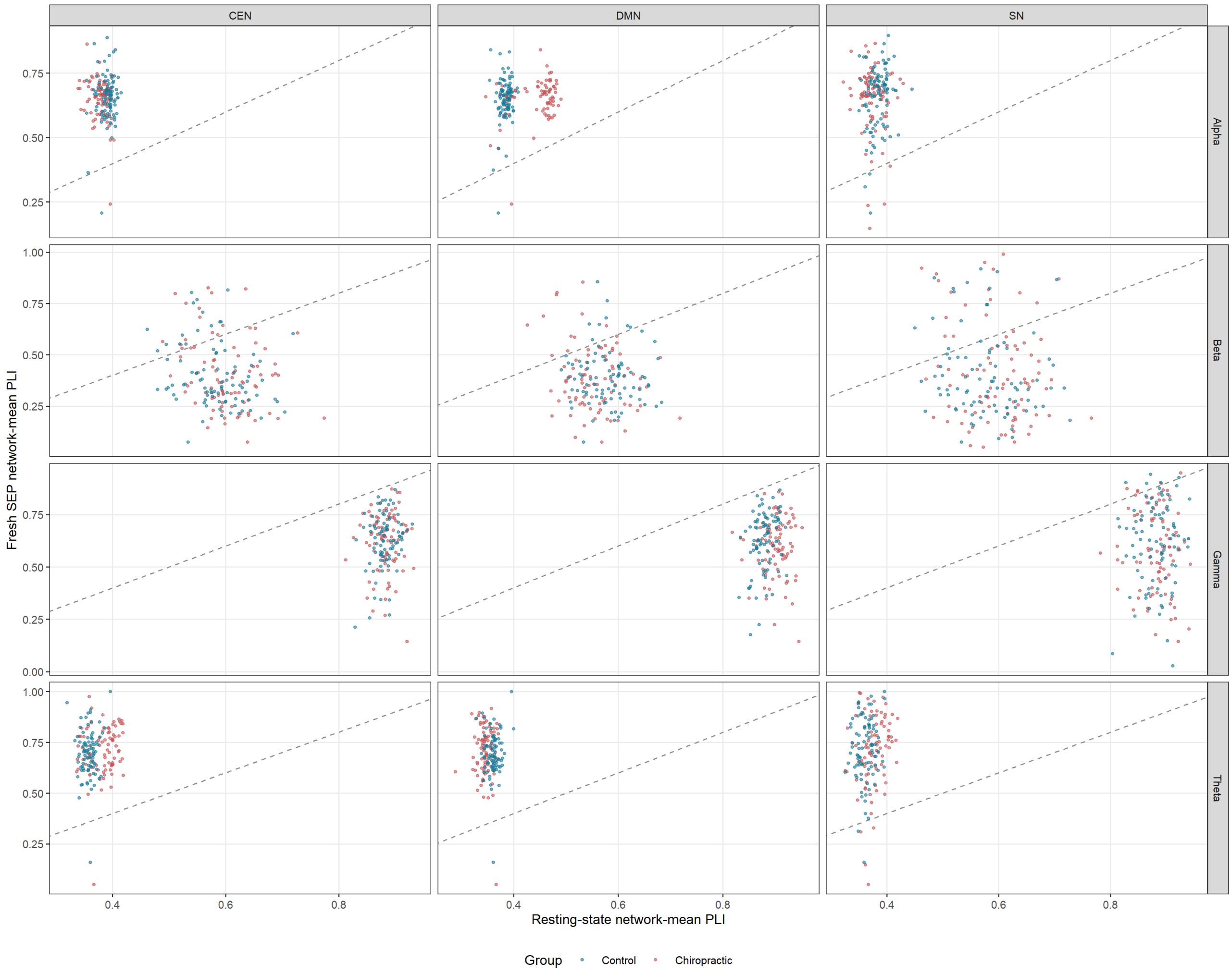

Figure S8: Participant-level resting-state versus SEP network-mean PLI, with the identity line shown. Points do not fall on the identity line, confirming the two conditions are numerically distinct.

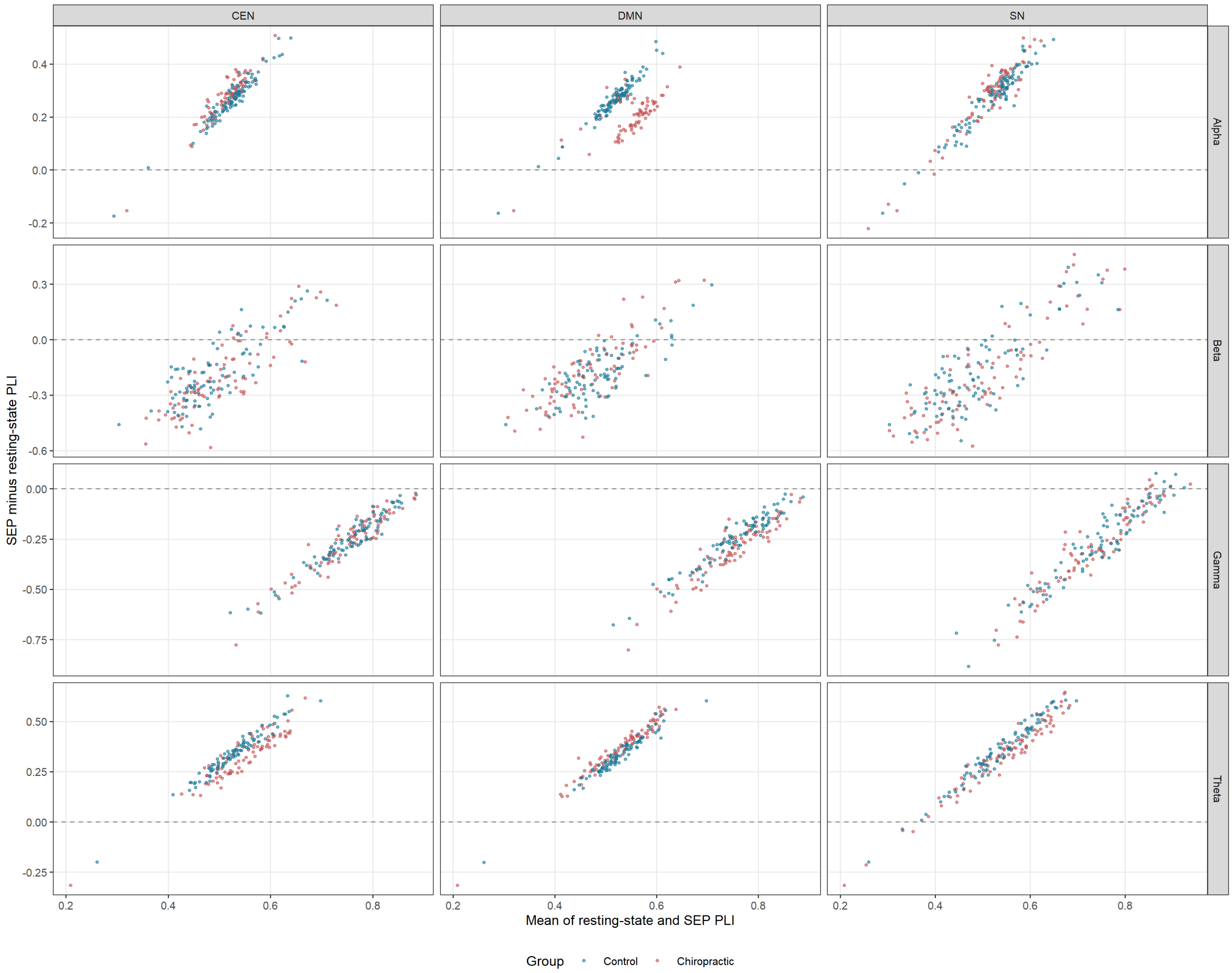

Figure S9: Bland–Altman comparison of participant-level SEP and resting-state network-mean PLI (differences are SEP minus resting).

S11. Region-level components (resting-state)

The resting-state analysis considered the same 12 band-by-contrast comparisons used for SEP. No theta- or gamma-band link reached the $p < .05$ component-forming threshold, so those six comparisons produced no connected component. The six alpha- and beta-band comparisons produced 14 components. Holm correction was applied across those 14 GraphVar node-count p_{FWE} values. Edge extent, defined as the number of suprathreshold links in a component, was calculated from the same saved GraphVar permutation masks and evaluated in a separate post-hoc sensitivity family. The node-count analysis is the prespecified statistic and the sole basis for component-level inference in the main text; the edge-extent analysis is reported here only and does not confer support on any component that did not retain node-count support. The p-value listed for each ROI pair is its link-level GraphVar permutation p-value, obtained by comparing the observed non-directional F statistic with the null distribution for that link across 5,000 permutations; $p < .05$ determined component membership.

Table S12: Resting-state node-count analysis. The table shows the largest component from each comparison; Holm correction was applied across all 14 extracted components.

**Band**	**Contrast**	**Nodes**	**Links**	**Node p_{FWE}**	**Holm p**	**Support**
Theta	Pre vs Post	–	–	–	–	No component
Theta	Pre vs Post-4W	–	–	–	–	No component
Theta	Post vs Post-4W	–	–	–	–	No component
Alpha	Pre vs Post	27	42	.0030	.0420	Yes
Alpha	Pre vs Post-4W	16	20	.4459	1.000	–
Alpha	Post vs Post-4W	13	12	.6023	1.000	–
Beta	Pre vs Post	15	18	.4909	1.000	–
Beta	Pre vs Post-4W	11	10	.6783	1.000	–
Beta	Post vs Post-4W	14	13	.5345	1.000	–
Gamma	Pre vs Post	–	–	–	–	No component
Gamma	Pre vs Post-4W	–	–	–	–	No component
Gamma	Post vs Post-4W	–	–	–	–	No component

Table S13: Resting-state edge-extent sensitivity analysis. The table shows the largest component from each comparison; Holm correction was applied separately across all 14 extracted components.

**Band**	**Contrast**	**Nodes**	**Links**	**Extent p_{FWE}**	**Holm p**	**Extent flag**
Theta	Pre vs Post	–	–	–	–	No component
Theta	Pre vs Post-4W	–	–	–	–	No component
Theta	Post vs Post-4W	–	–	–	–	No component
Alpha	Pre vs Post	27	42	.0002	.0028	Flagged
Alpha	Pre vs Post-4W	16	20	.2939	1.000	–
Alpha	Post vs Post-4W	13	12	.6397	1.000	–
Beta	Pre vs Post	15	18	.3721	1.000	–
Beta	Pre vs Post-4W	11	10	.7035	1.000	–
Beta	Post vs Post-4W	14	13	.5861	1.000	–
Gamma	Pre vs Post	–	–	–	–	No component
Gamma	Pre vs Post-4W	–	–	–	–	No component
Gamma	Post vs Post-4W	–	–	–	–	No component

The tables below give the complete membership of the largest component from each alpha- and beta-band comparison.

Table S14: Full membership of the resting-state alpha-band Pre versus Post component (27 nodes, 42 links; node-count $p_{FWE} = .0030$).

Region 1	Region 2	t	p value
R.CMF	R.MOF	+2.01	.0314
R.INS	L.PCG	+2.22	.0142
R.MOF	L.PCG	+2.05	.0292
R.PHIP	L.PCG	+2.77	.0256
R.MT	R.PCG	+2.15	.0328
R.PHIP	R.PCG	+2.88	.0074
L.PREC	R.PREC	+1.88	.0326
R.MOF	R.RAC	+1.79	.0478
L.PCG	R.RAC	+2.40	.0194
L.CAC	L.RMF	-2.61	.0086
R.CAC	L.RMF	-2.50	.0104
R.IST	L.RMF	+1.95	.0132
R.MOF	L.RMF	+3.47	<.001
R.RAC	L.RMF	+2.20	.0406
L.INS	R.RMF	-1.94	.0276
R.IST	R.RMF	+1.63	.0428
L.MT	L.SF	+2.04	.0432
L.PHIP	L.SF	+2.46	.0152
L.PCG	L.SF	+2.64	.0048
R.PCG	L.SF	+2.58	.0088
L.PREC	L.SF	+2.99	.0212
L.CAC	R.SF	+1.98	.0342
L.INS	R.SF	+2.27	.0120
L.MOF	R.SF	+1.96	.0096
R.MT	R.SF	+2.80	.0102
R.PHIP	R.SF	+2.06	.0290
R.PCG	R.SF	+2.29	.0162
L.RAC	R.SF	+2.44	.0096
L.RMF	R.SF	+2.42	.0108
R.RMF	R.SF	+2.30	.0204
R.INS	L.SP	+2.17	.0134
L.IST	L.SP	+2.01	.0368
R.MOF	L.SP	+2.21	.0176
L.MT	L.SP	+2.14	.0186
R.MT	L.SP	+2.15	.0264
L.PHIP	L.SP	+2.01	.0442
R.PHIP	L.SP	+2.87	.0054
R.PCG	L.SP	+1.94	.0456
R.SF	L.SP	+2.07	.0396
L.IP	R.SP	+2.27	.0358
R.IP	R.SP	-3.05	.0008
L.MOF	R.SP	-2.32	.0262

Table S15: Full membership of the largest resting-state alpha-band Pre versus Post-4W component (16 nodes, 20 links; node-count $p_{FWE} = .4459$).

Region 1	Region 2	t	p value
R.MT	L.PHIP	+2.19	.0446
R.IST	L.PCG	+2.40	.0186
R.IP	L.PREC	+2.34	.0304
L.IST	L.PREC	+1.94	.0488
R.PCG	L.PREC	+1.84	.0462
L.PCG	R.PREC	+2.93	.0048
R.PCG	R.PREC	+2.50	.0032

Region 1	Region 2	t	p value
R.IST	R.RAC	+1.78	.0434
R.IST	L.RMF	+1.89	.0344
R.MOF	L.RMF	+2.18	.0226
R.PHIP	L.RMF	+2.11	.0242
L.PCG	L.RMF	+2.05	.0328
R.PCG	L.RMF	+2.36	.0166
L.PREC	L.RMF	+2.11	.0402
L.PHIP	L.SF	+2.50	.0300
L.PHIP	R.SF	+1.91	.0462
R.MT	L.SP	+1.89	.0444
L.PHIP	L.SP	+2.45	.0338
R.PCG	L.SP	+2.74	.0026
L.RMF	L.SP	+2.43	.0064

Table S16: Full membership of the largest resting-state alpha-band Post versus Post-4W component (13 nodes, 12 links; node-count $p_{FWE} = .6023$).

Region 1	Region 2	t	p value
R.CAC	L.IST	+1.98	.0418
L.IST	R.IST	+2.23	.0178
R.INS	L.PCG	-2.05	.0280
R.MOF	L.PCG	-2.44	.0120
R.MOF	R.PCG	-2.03	.0364
R.IST	L.PREC	+1.96	.0368
R.PCG	R.PREC	+2.08	.0150
L.PREC	R.PREC	-1.83	.0410
L.CAC	L.RMF	+1.86	.0470
R.CAC	L.RMF	+1.84	.0378
R.IP	R.SP	+3.04	.0022
R.PCG	R.SP	+2.29	.0274

Table S17: Full membership of the largest resting-state beta-band Pre versus Post component (15 nodes, 18 links; node-count $p_{FWE} = .4909$).

Region 1	Region 2
L.CMF	L.INS
L.CMF	L.PREC
L.CMF	R.MOF
L.CMF	R.PHIP
L.INS	R.MOF
L.INS	R.SF
L.MT	R.SF
L.PHIP	R.RAC
R.CAC	L.INS
R.CAC	L.PHIP
R.CAC	R.MOF
R.CMF	R.MOF
R.INS	R.IST
R.INS	R.PHIP
R.INS	R.PREC
R.IST	L.MOF
R.MOF	L.PREC
R.PHIP	R.PREC

Table S18: Full membership of the largest resting-state beta-band Pre versus Post-4W component (11 nodes, 10 links; node-count $p_{FWE} = .6783$).

Region 1	Region 2	t	p value
L.IP	R.INS	+1.90	.0340
L.CMF	R.MOF	+2.10	.0422
L.MOF	L.PHIP	+2.04	.0448
R.MOF	L.PHIP	+2.50	.0222
R.INS	R.PHIP	+1.81	.0398
L.MOF	R.PHIP	+2.11	.0340
L.MOF	L.PCG	+2.04	.0454
R.PHIP	R.PREC	+2.15	.0298
L.PHIP	L.RAC	+2.31	.0338
L.PHIP	R.RAC	+2.06	.0384

Table S19: Full membership of the largest resting-state beta-band Post versus Post-4W component (14 nodes, 13 links; node-count $p_{FWE} = .5345$).

Region 1	Region 2	t	p value
L.CAC	L.INS	-2.77	.0042
R.CAC	L.INS	-3.65	.0002
R.CMF	L.INS	-2.61	.0118
R.INS	L.IST	-2.01	.0406
R.INS	R.IST	-2.31	.0106
R.IST	L.MOF	-2.35	.0164
L.INS	R.MOF	-2.05	.0490
L.IP	R.PCG	-2.19	.0434
L.IST	R.PCG	+1.81	.0406
R.MOF	R.PCG	-2.18	.0240
L.IP	R.PREC	-2.25	.0382
R.IST	L.RAC	-2.52	.0116
L.IST	R.RAC	-2.30	.0204

S12. Region-level components (SEP)

The SEP region-level analysis used the same procedure as the resting-state analysis for the same 12 band-by-contrast comparisons. These comparisons produced 24 components. Holm correction was applied across the 24 GraphVar node-count p_{FWE} values (Table S20). Edge extent, defined as the number of suprathreshold links in a component, was calculated from the same saved GraphVar permutation masks and evaluated in a separate post-hoc sensitivity family (Table S21). The node-count analysis is the prespecified statistic and the sole basis for component-level inference in the main text; the edge-extent analysis is reported here only and does not confer support on any component that did not retain node-count support. The largest component in four comparisons had a node-count $p_{FWE} < .05$. The p-value listed for each ROI pair is its link-level GraphVar permutation p-value, obtained by comparing the observed non-directional F statistic with the null distribution for that link across 5,000 permutations; $p < .05$ determined component membership.

Table S20: SEP node-count analysis. The table shows the largest component from each comparison; Holm correction was applied across all 24 extracted components.

Band	Contrast	Nodes	Links	Node p_{FWE}	Holm p	Support
Theta	Post vs Post-4W	28	86	.0020	.048	Yes
Theta	Pre vs Post	28	88	.0046	.106	–
Gamma	Pre vs Post	28	70	.0098	.216	–
Alpha	Pre vs Post	27	76	.0302	.634	–
Beta	Post vs Post-4W	25	33	.090	1.000	–
Beta	Pre vs Post	21	36	.196	1.000	–
Gamma	Pre vs Post-4W	21	23	.273	1.000	–
Alpha	Post vs Post-4W	18	27	.329	1.000	–
Alpha	Pre vs Post-4W	18	19	.348	1.000	–
Beta	Pre vs Post-4W	14	16	.529	1.000	–
Theta	Pre vs Post-4W	4	4	.877	1.000	–
Gamma	Post vs Post-4W	5	4	.924	1.000	–

Table S21: SEP edge-extent sensitivity analysis. The table shows the largest component from each comparison; Holm correction was applied separately across all 24 extracted components.

Band	Contrast	Nodes	Links	Extent p_{FWE}	Holm p	Extent flag
Theta	Post vs Post-4W	28	86	.0002	.0048	Flagged
Theta	Pre vs Post	28	88	.0002	.0048	Flagged
Gamma	Pre vs Post	28	70	.0002	.0048	Flagged
Alpha	Pre vs Post	27	76	.0002	.0048	Flagged
Beta	Post vs Post-4W	25	33	.100	1.000	–
Beta	Pre vs Post	21	36	.064	1.000	–
Gamma	Pre vs Post-4W	21	23	.285	1.000	–
Alpha	Post vs Post-4W	18	27	.196	1.000	–
Alpha	Pre vs Post-4W	18	19	.352	1.000	–
Beta	Pre vs Post-4W	14	16	.453	1.000	–
Theta	Pre vs Post-4W	4	4	.825	1.000	–
Gamma	Post vs Post-4W	5	4	.927	1.000	–

Figures S10–S13 show the complete components corresponding to the four node-count $p_{FWE} < .05$ results. Gold and blue edges indicate positive and negative GraphVar coefficients, respectively; edge width reflects effect magnitude.

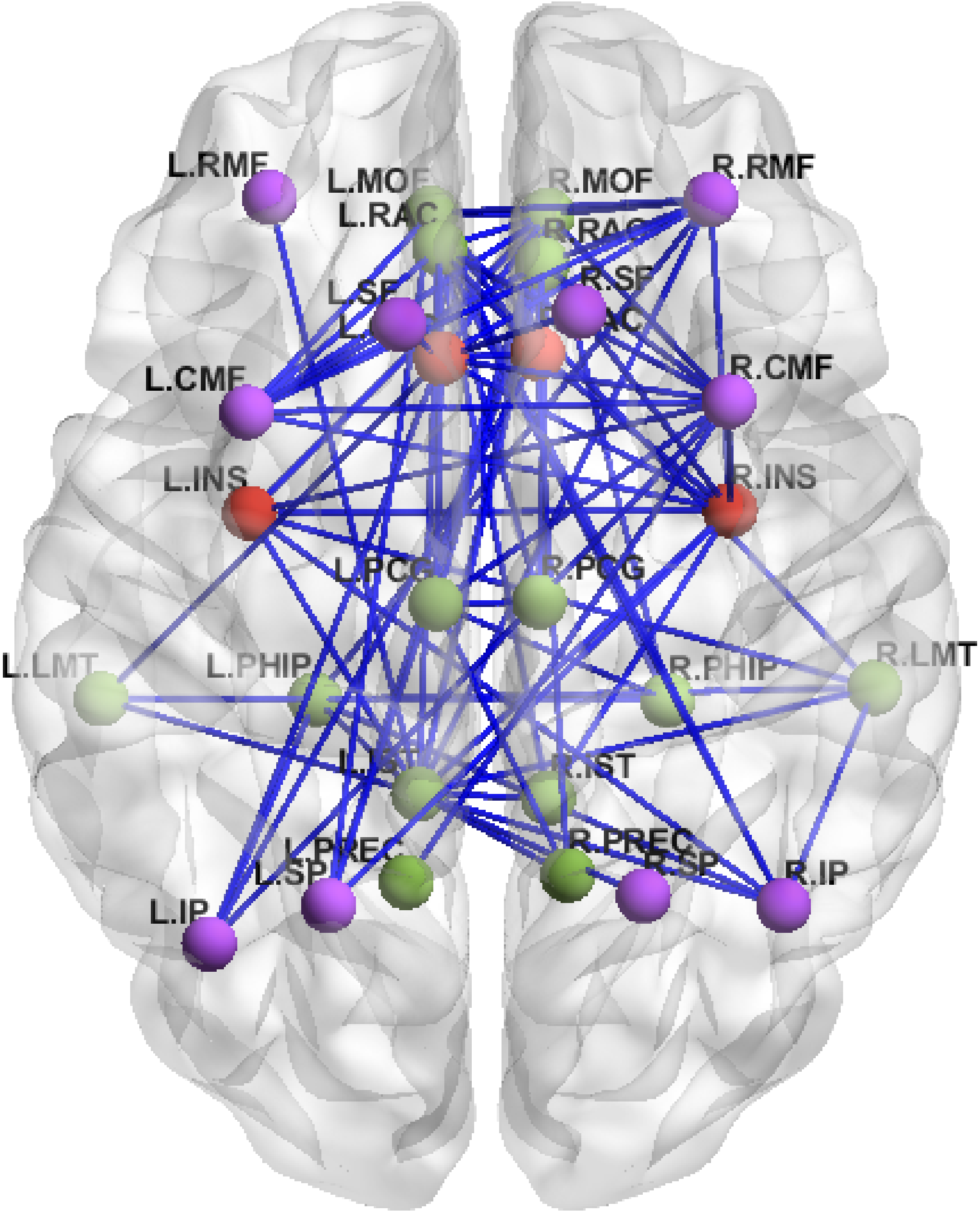

Figure S10: Complete SEP theta-band Post versus Post-4W component (28 nodes, 86 links). Correction results are reported in Tables S20 and S21.

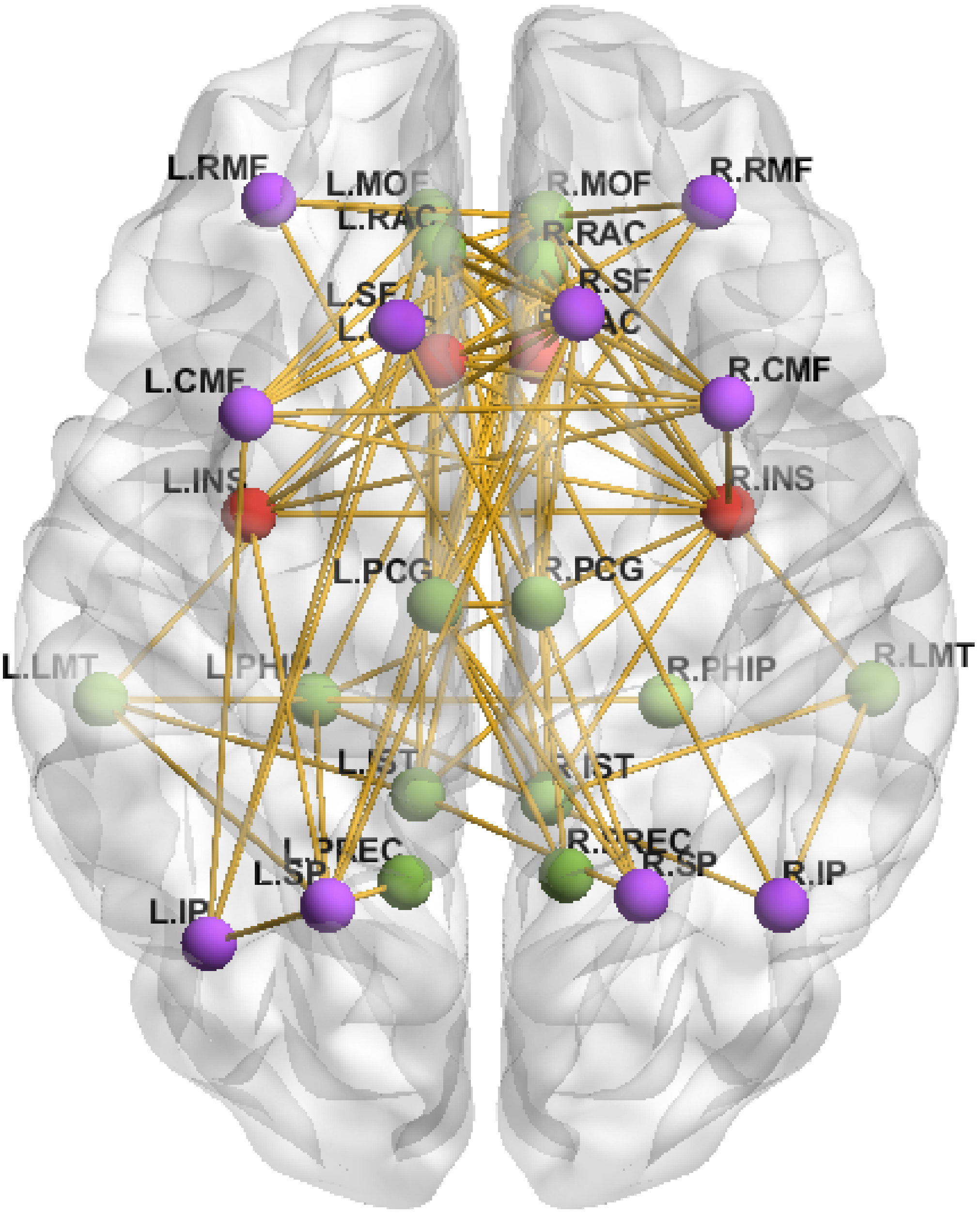

Figure S11: Complete SEP theta-band Pre versus Post component (28 nodes, 88 links). Correction results are reported in Tables S20 and S21.

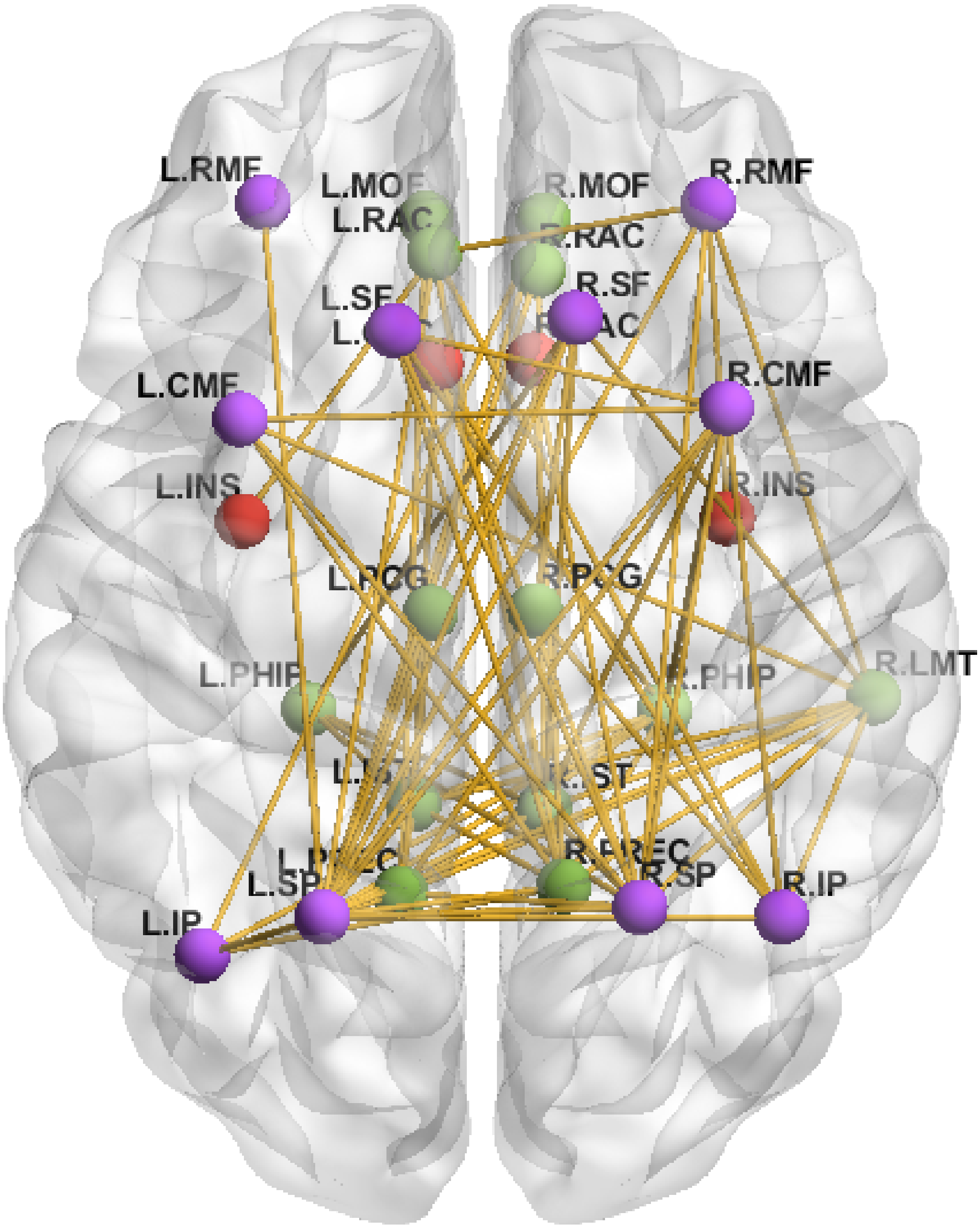

Figure S12: Complete SEP alpha-band Pre versus Post component (27 nodes, 76 links). Correction results are reported in Tables S20 and S21.

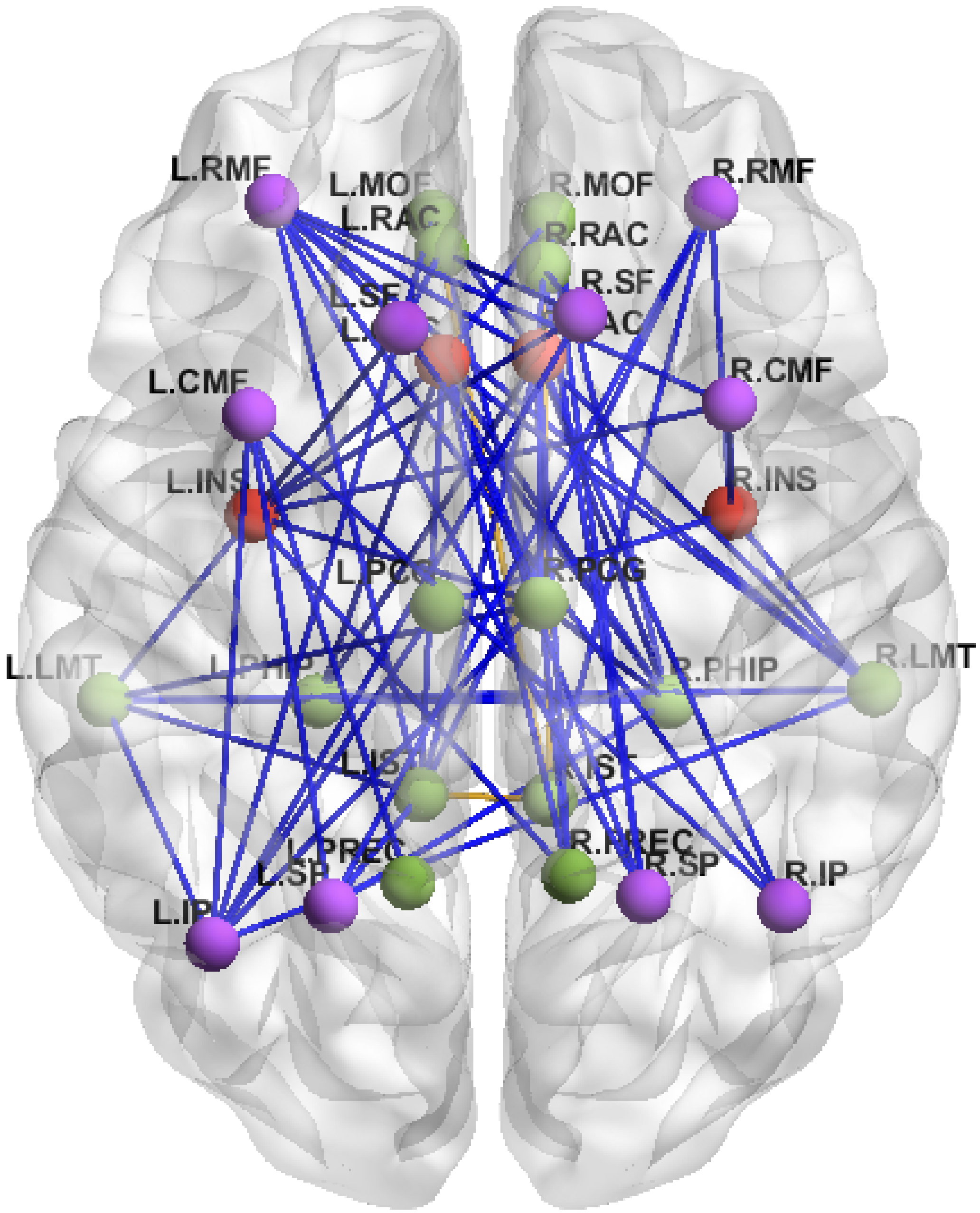

Figure S13: Complete SEP gamma-band Pre versus Post component (28 nodes, 70 links). Correction results are reported in Tables S20 and S21.

Table S22: Full membership of the SEP theta-band Post versus Post-4W component (28 nodes, 86 links). Direction is the sign of the GraphVar coefficient.

Region 1	Region 2	F	p value	Dir.
L.CAC	R.CAC	4.01	.046	–
L.CAC	R.CMF	4.14	.026	–
L.CMF	R.CMF	8.16	.0004	–
L.CAC	L.IP	5.79	.021	–
R.CAC	L.IP	6.63	.013	–
R.CMF	L.IP	7.74	.0032	–
R.CMF	L.INS	4.54	.038	–
R.IP	L.INS	5.77	.011	–
L.CAC	R.INS	14.81	$< .0002$	–
R.CAC	R.INS	11.86	.0002	–
L.CMF	R.INS	8.40	.0050	–
R.CMF	R.INS	14.54	$< .0002$	–
L.INS	R.INS	4.67	.045	–
R.IP	L.IST	4.80	.026	–
L.INS	L.IST	5.17	.012	–
R.INS	L.IST	9.25	.0050	–
R.INS	R.IST	5.71	.015	–
L.IST	R.IST	3.19	.047	–
L.CAC	L.MOF	12.27	.0012	–
R.CAC	L.MOF	16.68	.0002	–
L.CMF	L.MOF	3.95	.015	–
R.IP	L.MOF	5.05	.0066	–
R.IST	L.MOF	6.44	.010	–
L.CAC	R.MOF	6.72	.011	–
R.CAC	R.MOF	4.60	.024	–
L.CMF	R.MOF	8.53	.0030	–
R.CMF	R.MOF	8.80	.0026	–
L.INS	R.MOF	6.94	.0036	–
L.IST	R.MOF	11.06	.0010	–
L.MOF	R.MOF	8.30	.0022	–
L.CAC	L.MT	3.42	.034	–
L.IST	L.MT	3.95	.041	–
R.IP	R.MT	3.94	.019	–
L.INS	R.MT	6.80	.0036	–
L.IST	R.MT	3.26	.046	–
L.MOF	R.MT	6.21	.0074	–
L.IP	L.PHIP	3.97	.032	–
R.IP	L.PHIP	2.90	.045	–
L.IST	L.PHIP	4.25	.041	–
L.MT	L.PHIP	5.08	.020	–
R.MT	L.PHIP	3.31	.023	–
L.MOF	R.PHIP	4.73	.038	–
R.MOF	R.PHIP	2.79	.042	–
L.MT	R.PHIP	6.95	.0048	–
L.CAC	L.PCG	5.41	.013	–
R.CMF	L.PCG	4.88	.041	–
R.MOF	L.PCG	9.50	.0002	–
R.PHIP	L.PCG	5.32	.025	–
L.MOF	R.PCG	4.62	.029	–
R.MOF	R.PCG	10.59	$< .0002$	–
L.PCG	R.PCG	4.93	.029	–
R.CMF	L.PREC	5.56	.0094	–
R.CAC	R.PREC	3.94	.020	–
L.IST	R.PREC	5.20	.014	–
L.PCG	R.PREC	4.27	.026	–
L.CAC	L.RAC	7.11	.0036	–

Region 1	Region 2	F	p value	Dir.
R.CAC	L.RAC	6.15	.0070	−
L.CMF	L.RAC	9.54	.0018	−
R.CMF	L.RAC	10.50	.0002	−
R.IP	L.RAC	4.99	.021	−
L.IST	L.RAC	5.62	.025	−
L.MOF	L.RAC	5.94	.017	−
R.MOF	L.RAC	10.15	.0012	−
R.CAC	R.RAC	3.72	.043	−
L.CMF	R.RAC	7.29	.015	−
R.CMF	R.RAC	5.96	.0034	−
L.IST	R.RAC	11.17	.0002	−
R.MOF	R.RAC	6.56	.012	−
L.IST	L.RMF	6.76	.0070	−
L.CAC	R.RMF	9.37	.0036	−
L.CMF	R.RMF	3.40	.040	−
R.INS	R.RMF	4.11	.037	−
L.IST	R.RMF	3.11	.033	−
L.MOF	R.RMF	7.66	.018	−
R.MOF	R.RMF	15.27	$< .0002$	−
L.PHIP	R.RMF	7.17	.0082	−
R.RAC	R.RMF	4.18	.033	−
L.CAC	L.SF	5.14	.028	−
L.CMF	L.SF	4.46	.034	−
L.CAC	R.SF	4.40	.031	−
R.INS	R.SF	6.05	.0064	−
R.INS	L.SP	4.34	.043	−
L.MOF	L.SP	7.90	.0044	−
R.MOF	L.SP	4.35	.026	−
R.RAC	L.SP	9.26	.0026	−
L.IST	R.SP	4.21	.024	−

Table S23: Full membership of the SEP theta-band Pre versus Post component (28 nodes, 88 links). Direction is the sign of the GraphVar coefficient.

Region 1	Region 2	F	p value	Dir.
L.CAC	R.CAC	6.95	.012	+
L.CAC	R.CMF	5.24	.0078	+
L.CMF	R.CMF	11.99	.0006	+
L.CMF	L.IP	4.86	.017	+
R.CMF	L.INS	4.52	.016	+
L.CAC	R.INS	10.12	.0004	+
R.CAC	R.INS	11.22	.0004	+
L.CMF	R.INS	5.62	.026	+
R.CMF	R.INS	5.29	.0080	+
L.INS	R.INS	9.57	.0024	+
R.INS	L.IST	9.04	.017	+
R.INS	R.IST	4.59	.030	+
L.CMF	L.MOF	5.34	.022	+
R.CMF	L.MOF	4.85	.018	+
R.IP	L.MOF	4.87	.020	+
R.IST	L.MOF	4.21	.045	+
R.CAC	R.MOF	3.89	.028	+
L.CMF	R.MOF	5.45	.022	+
R.CMF	R.MOF	5.27	.016	+
L.INS	R.MOF	7.17	.0044	+
R.INS	R.MOF	5.19	.035	+
L.IST	R.MOF	6.49	.013	+

Region 1	Region 2	F	p value	Dir.
R.IST	R.MOF	3.65	.042	+
L.IST	L.MT	4.33	.026	+
R.MOF	L.MT	7.79	.0046	+
R.IP	R.MT	4.34	.049	+
R.INS	R.MT	4.25	.033	+
R.IST	R.MT	4.08	.023	+
R.IP	L.PHIP	2.94	.048	+
R.INS	L.PHIP	8.10	.0010	+
R.MOF	L.PHIP	6.16	.0072	+
L.MT	R.PHIP	4.94	.049	+
R.IST	L.PCG	6.28	.017	+
L.MOF	L.PCG	6.25	.020	+
R.MOF	L.PCG	12.09	.0004	+
L.MOF	R.PCG	6.95	.0022	+
R.MOF	R.PCG	15.62	$< .0002$	+
L.PCG	R.PCG	5.96	.014	+
L.IP	L.PREC	18.67	$< .0002$	+
L.PCG	R.PREC	13.29	.0004	+
R.PCG	R.PREC	6.72	.0034	+
L.CAC	L.RAC	4.16	.031	+
R.CAC	L.RAC	5.32	.013	+
L.CMF	L.RAC	11.72	.0018	+
R.CMF	L.RAC	4.45	.020	+
L.IP	L.RAC	5.63	.014	+
R.INS	L.RAC	5.77	.015	+
L.IST	L.RAC	10.99	.0006	+
R.IST	L.RAC	9.19	.0022	+
R.MOF	L.RAC	13.16	.0006	+
L.PCG	L.RAC	4.92	.018	+
R.CAC	R.RAC	5.20	.026	+
L.CMF	R.RAC	11.43	.0022	+
L.IST	R.RAC	4.97	.013	+
R.MOF	R.RAC	6.38	.014	+
L.PCG	R.RAC	5.70	.018	+
R.PCG	R.RAC	5.86	.014	+
L.RAC	R.RAC	5.30	.022	+
L.MOF	L.RMF	8.05	.0082	+
R.MOF	L.RMF	6.22	.0078	+
L.CAC	R.RMF	7.51	.0014	+
R.MOF	R.RMF	8.34	.0044	+
L.PCG	R.RMF	9.19	.0008	+
L.IP	L.SF	5.96	.0062	+
L.MOF	L.SF	5.04	.044	+
R.PCG	L.SF	6.41	.0092	+
L.CAC	R.SF	8.88	.0028	+
R.CAC	R.SF	10.16	.0018	+
L.INS	R.SF	3.10	.042	+
R.INS	R.SF	6.81	.0028	+
L.MOF	R.SF	3.62	.039	+
R.MOF	R.SF	6.26	.0022	+
L.PCG	R.SF	4.11	.039	+
R.PCG	R.SF	4.70	.032	+
L.RAC	R.SF	4.62	.048	+
R.RAC	R.SF	6.37	.0022	+
L.IP	L.SP	4.46	.0080	+
L.INS	L.SP	5.61	.021	+
R.MOF	L.SP	8.57	.0032	+
L.MT	L.SP	3.32	.048	+
L.PHIP	L.SP	4.41	.029	+

Region 1	Region 2	F	p value	Dir.
L.PREC	L.SP	4.46	.034	+
R.RAC	L.SP	5.91	.011	+
R.SF	L.SP	4.54	.018	+
L.IST	R.SP	4.58	.033	+
L.PCG	R.SP	4.19	.044	+
R.PCG	R.SP	5.72	.0048	+
L.RMF	R.SP	3.90	.040	+

Table S24: Full membership of the SEP alpha-band Pre versus Post component (27 nodes, 76 links). Direction is the sign of the GraphVar coefficient.

Region 1	Region 2	F	p value	Dir.
L.CMF	R.CMF	5.09	.020	+
L.CAC	R.IP	7.15	.014	+
R.CMF	R.IP	10.28	.0028	+
L.CAC	L.IST	5.15	.037	+
R.CMF	R.IST	5.23	.028	+
R.IST	L.MOF	5.96	.018	+
L.IP	R.MOF	5.76	.018	+
L.CMF	R.MT	8.23	.0082	+
R.IP	R.MT	7.68	.014	+
L.IST	R.MT	5.21	.023	+
R.IST	R.MT	11.42	.0012	+
L.IST	L.PHIP	7.98	.011	+
R.IST	L.PHIP	5.56	.042	+
R.CMF	R.PHIP	5.30	.027	+
L.IP	R.PHIP	4.04	.035	+
L.IST	R.PHIP	5.59	.012	+
L.IST	L.PCG	8.45	.015	+
R.IP	R.PCG	8.28	.0028	+
R.CMF	L.PREC	8.10	.013	+
L.IP	L.PREC	12.57	.0028	+
L.IST	L.PREC	11.09	.0016	+
L.MOF	L.PREC	4.57	.049	+
R.MT	L.PREC	5.51	.049	+
R.PHIP	L.PREC	9.25	.0012	+
L.PCG	L.PREC	7.33	.014	+
R.CAC	R.PREC	8.85	.0074	+
L.CMF	R.PREC	11.07	.0030	+
L.IP	R.PREC	6.66	.013	+
L.IST	R.PREC	2.62	.038	+
L.MOF	R.PREC	5.67	.013	+
R.MT	R.PREC	7.22	.019	+
R.PHIP	R.PREC	5.44	.014	+
R.IP	L.RAC	7.26	.0040	+
L.INS	L.RAC	7.06	.015	+
R.IST	R.RAC	4.67	.032	+
R.INS	R.RMF	5.24	.022	+
R.MT	R.RMF	4.67	.049	+
L.RAC	R.RMF	5.15	.034	+
R.CMF	L.SF	3.87	.047	+
R.PHIP	L.SF	2.82	.047	+
R.PREC	L.SF	5.44	.013	+
R.IST	R.SF	4.75	.023	+
R.MT	R.SF	5.47	.029	+
L.PHIP	R.SF	5.47	.019	+
L.PCG	R.SF	9.98	.0082	+

Region 1	Region 2	F	p value	Dir.
R.PCG	R.SF	10.49	.0004	+
R.CMF	L.SP	5.24	.037	+
L.IP	L.SP	9.74	.0008	+
R.IP	L.SP	5.19	.013	+
L.IST	L.SP	8.62	.0036	+
R.IST	L.SP	4.39	.049	+
R.MOF	L.SP	4.63	.033	+
R.MT	L.SP	8.70	.0030	+
L.PHIP	L.SP	5.43	.023	+
R.PHIP	L.SP	6.79	.0034	+
L.PCG	L.SP	8.83	.0032	+
R.PCG	L.SP	7.67	.0070	+
L.PREC	L.SP	4.78	.015	+
R.PREC	L.SP	6.34	.0026	+
L.RAC	L.SP	13.67	.0010	+
R.RAC	L.SP	8.04	.0094	+
L.RMF	L.SP	10.85	.0004	+
R.RMF	L.SP	10.92	.0006	+
R.SF	L.SP	3.37	.034	+
L.CAC	R.SP	5.35	.010	+
R.CAC	R.SP	6.33	.018	+
L.CMF	R.SP	9.53	.0056	+
R.CMF	R.SP	4.06	.030	+
R.IST	R.SP	3.34	.037	+
R.MT	R.SP	9.13	.0030	+
L.PHIP	R.SP	6.33	.0048	+
R.PHIP	R.SP	3.79	.020	+
L.PREC	R.SP	6.14	.021	+
R.RMF	R.SP	7.12	.016	+
L.SF	R.SP	5.78	.0080	+
L.SP	R.SP	11.24	$< .0002$	+

Table S25: Full membership of the SEP gamma-band Pre versus Post component (28 nodes, 70 links). Direction is the sign of the GraphVar coefficient.

Region 1	Region 2	F	p value	Dir.
L.CMF	L.IP	9.40	.0046	−
R.CAC	R.IP	4.46	.045	−
L.CMF	R.IP	6.97	.0098	−
L.CAC	L.INS	2.85	.047	−
R.CMF	L.INS	3.66	.050	−
R.CMF	R.INS	10.35	.0004	−
L.CAC	L.IST	4.96	.017	−
R.CAC	L.IST	3.97	.035	−
L.IST	R.IST	3.29	.031	+
R.CAC	L.MOF	4.29	.032	−
L.CAC	R.MOF	3.43	.044	−
L.IP	L.MT	8.26	.0006	−
L.INS	L.MT	6.14	.0058	−
L.IST	L.MT	4.78	.016	−
L.CAC	R.MT	7.69	.0032	−
R.CAC	R.MT	5.24	.018	−
L.IP	R.MT	5.11	.013	−
R.INS	R.MT	4.73	.011	−
L.MT	R.MT	4.14	.033	−
L.CAC	L.PHIP	10.23	.0006	−
R.CAC	L.PHIP	10.34	.0016	−

Region 1	Region 2	F	p value	Dir.
R.MT	L.PHIP	12.71	$< .0002$	−
L.CAC	R.PHIP	8.75	.0032	−
R.CAC	R.PHIP	12.21	.0006	−
R.MOF	R.PHIP	4.35	.014	−
L.MT	R.PHIP	10.49	.0016	−
L.PHIP	R.PHIP	8.66	.0018	−
L.IP	L.PCG	5.14	.026	−
R.INS	L.PCG	3.02	.049	−
L.CAC	R.PCG	7.27	.0066	−
R.CAC	R.PCG	6.43	.019	−
L.IP	R.PCG	7.50	.010	−
L.INS	R.PCG	5.89	.0078	−
L.MOF	R.PCG	3.63	.033	−
L.MT	R.PCG	7.02	.0036	−
R.PHIP	R.PCG	7.66	.0088	−
L.CMF	L.PREC	8.10	.013	−
L.CAC	R.PREC	5.71	.032	−
R.CAC	R.PREC	7.53	.024	−
L.INS	R.PREC	4.82	.013	−
R.PCG	R.PREC	4.01	.025	−
R.IST	L.RAC	5.18	.022	+
R.CAC	R.RAC	8.06	.0044	−
R.IST	R.RAC	5.28	.017	+
L.CAC	L.RMF	3.74	.044	−
R.CAC	L.RMF	5.31	.023	−
R.CMF	L.RMF	3.88	.041	−
R.IP	L.RMF	4.55	.036	−
L.IST	L.RMF	6.70	.0066	−
R.MT	L.RMF	3.53	.019	−
R.INS	R.RMF	3.40	.037	−
L.IST	R.RMF	5.04	.019	−
R.IST	R.RMF	4.36	.023	−
L.IP	L.SF	7.11	.0086	−
L.INS	L.SF	4.99	.014	−
L.MOF	L.SF	7.34	.0040	−
R.PHIP	L.SF	4.03	.039	−
L.RAC	L.SF	2.54	.049	−
L.IP	R.SF	5.26	.020	−
L.INS	R.SF	5.91	.026	−
L.RAC	R.SF	6.37	.0062	−
R.RAC	R.SF	5.39	.0072	−
L.CMF	L.SP	7.31	.042	−
L.IST	L.SP	4.46	.013	−
R.PHIP	L.SP	6.86	.0070	−
R.RMF	L.SP	7.00	.013	−
L.CAC	R.SP	6.81	.023	−
R.MOF	R.SP	4.55	.019	−
R.RAC	R.SP	2.57	.049	−
L.RMF	R.SP	5.54	.013	−
